# Supplementary material for: Preventing CpG hypermethylation in oocytes safeguards mouse development
Source: Dev Cell. 2025 Dec 1;60(23):3285–3303.e9. doi: 10.1016/j.devcel.2025.08.005 (PMC12687725; doi:10.1016/j.devcel.2025.08.005)
Supplement: Document S1. Figures S1–S7 [file mmc1.pdf]

Developmental Cell, Volume *60*

Supplemental information

Preventing CpG hypermethylation in oocytes safeguards mouse development

Yumiko K. Kawamura, Evgeniy A. Ozonov, Panagiotis Papasaikas, Takashi Kondo, Nhuong V. Nguyen, Michael B. Stadler, Sebastien A. Smallwood, Haruhiko Koseki, and Antoine H.F.M. Peters

Supplemental Information

Preventing CpG hypermethylation in oocytes safeguards mouse development

Yumiko K. Kawamura, Evgeniy A. Ozonov, Panagiotis Papasaikas, Takashi Kondo, Nhuong V. Nguyen, Michael B. Stadler, Sebastien A. Smallwood, Haruhiko Koseki and Antoine H.F.M Peters.

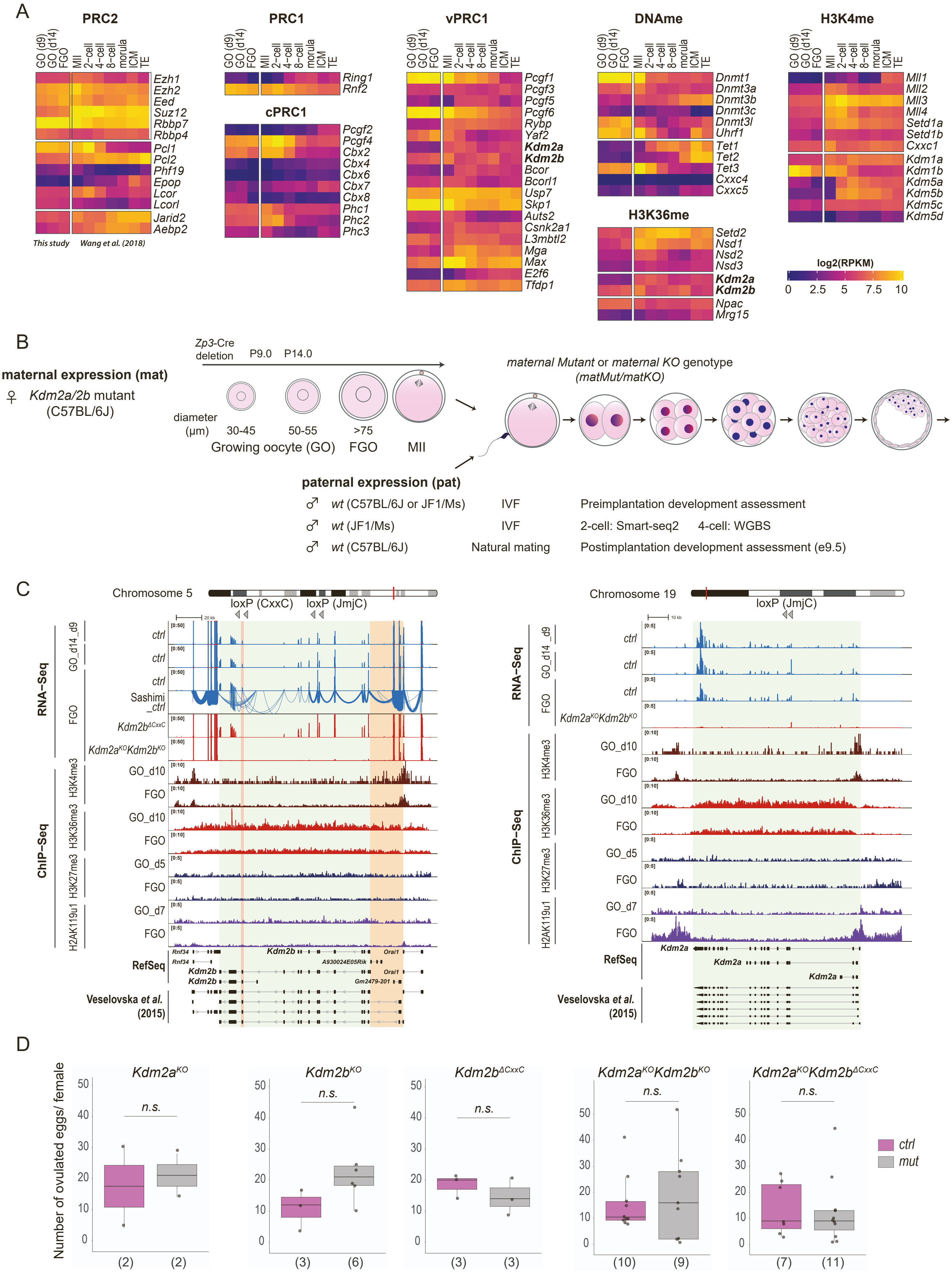

A
PRC2
GO (d9)
GO (d14)
FGO
MII
2-cell
4-cell
8-cell
morula
ICM
TE
Ezh1
Ezh2
Eed
Suz12
Rbbp7
Rbbp4
Pcl1
Pcl2
Phf19
Epop
Lcor
Lcorl
Jarid2
Aebp2
This study
Wang et al. (2018)
PRC1
Ring1
Rnf2
cPRC1
Pcgf2
Pcgf4
Cbx2
Cbx4
Cbx6
Cbx7
Cbx8
Phc1
Phc2
Phc3
vPRC1
Pcgf1
Pcgf3
Pcgf5
Pcgf6
Rybp
Yaf2
Kdm2a
Kdm2b
Bcor
Bcorl1
Usp7
Skp1
Auts2
Csnk2a1
L3mbtl2
Mga
Max
E2f6
Tfdp1
DNAme
Dnmt1
Dnmt3a
Dnmt3b
Dnmt3c
Dnmt3l
Uhrf1
Tet1
Tet2
Tet3
Cxxc4
Cxxc5
H3K36me
Setd2
Nsd1
Nsd2
Nsd3
Kdm2a
Kdm2b
Npac
Mrg15
H3K4me
Mll1
Mll2
Mll3
Mll4
Setd1a
Setd1b
Cxxc1
Kdm1a
Kdm1b
Kdm5a
Kdm5b
Kdm5c
Kdm5d
log2(RPKM)
0
2.5
5
7.5
10
B
maternal expression (mat)
♀ Kdm2a/2b mutant
(C57BL/6J)
Zp3-Cre
deletion
P9.0
P14.0
diameter
(μm)
30-45
50-55
>75
Growing oocyte (GO)
FGO
MII
maternal Mutant or maternal KO genotype
(matMut/matKO)
paternal expression (pat)
♂ wt (C57BL/6J or JF1/Ms)
IVF
Preimplantation development assessment
♂ wt (JF1/Ms)
IVF
2-cell: Smart-seq2
4-cell: WGBS
♂ wt (C57BL/6J)
Natural mating
Postimplantation development assessment (e9.5)
C
Chromosome 5
loxP (CxxC)
loxP (JmjC)
20 kb
RNA-Seq
GO_d14_d9
ctrl
ctrl
ctrl
FGO
Sashimi
_ctrl
Kdm2bΔCxxC
Kdm2aKOKdm2bKO
ChIP-Seq
H3K4me3
GO_d10
FGO
H3K36me3
GO_d10
FGO
H3K27me3
GO_d5
FGO
H2AK119u1
GO_d7
FGO
RefSeq
Rnf34
Kdm2b
Orai1
A930024E05Rik
Gm2479-201
Veselovska et al.
(2015)
Chromosome 19
loxP (JmjC)
10 kb
RNA-Seq
GO_d14_d9
FGO
ctrl
ctrl
ctrl
Kdm2aKOKdm2bKO
ChIP-Seq
H3K4me3
H3K36me3
H3K27me3
H2AK119u1
GO_d10
FGO
GO_d10
FGO
GO_d5
FGO
GO_d7
FGO
RefSeq
Kdm2a
Veselovska et al.
(2015)
D
Number of ovulated eggs/ female
Kdm2aKO
n.s.
(2)
(2)
Kdm2bKO
n.s.
(3)
(6)
Kdm2bΔCxxC
n.s.
(3)
(3)
Kdm2aKOKdm2bKO
n.s.
(10)
(9)
Kdm2aKOKdm2bΔCxxC
n.s.
(7)
(11)
ctrl
mut
0
10
20
30
40
50

Figure S1: Deficiency of *Kdm2a* and/or *Kdm2b* does not impair oogenesis, related to Figure 1.

- **A.** RNA expression of multiple genes in GOs (at day 9, day 14), FGOs and pre-implantation embryos at different stages of development [S1].
- **B.** Scheme to generate oocyte specific deficiency and maternally deficient embryos (*matKO*) by crossing conditional *Kdm2a*^{KO}, *Kdm2b*^{KO}, *Kdm2b*$^{\Delta CxxC}$, double or compound mutant females (on C57BL/6J genetic background) with *wt* males (JF1 or C57BL/6J genetic background) or by performing *in vitro* fertilization (IVF) followed by *in vitro* culture for several days, as indicated. Oocyte specific deletion was mediated by deletion of floxed alleles by CRE-recombinase, expressed from a *Zona pellucida 3*-cre (*Zp3*-cre) transgene initiated in primary GOs [S2]. Deletion of the JmjC domain encoding exons of *Kdm2a*$^{fl\text{-}JmjC}$ and *Kdm2b*$^{fl\text{-}JmjC}$ [S3] results in a translational frame shift, decay of mRNA transcripts and greatly reduced expression. Deletion of the CGI-binding Zinc Finger Domain “CxxC” encoding exon of *Kdm2b*$^{fl\text{-}CxxC}$ [S4] causes an in-frame excision leading to expression of a slightly smaller KDM2B protein unable to be recruited to CpG islands (see also Figure S1C) [S3, S4].
- **C.** (Left) RNA expression and chromatin status along the RefSeq-annotated *Kdm2b* locus (in reverse orientation; highlighted in green) in *ctrl* and various *Kdm2b* conditionally mutant oocytes. *Kdm2b* and neighboring *Rnf34* and *Orai1* genes are highly expressed throughout oogenesis and are associated with high H3K4me3 occupancy at gene promoters, widespread H3K36me3 enrichment along gene bodies and absence of repressive H3K27me3 and H2AK119u1. Chromatin and splice-junction analysis revealed that *Kdm2b* transcription in oocytes initiates from an alternative promoter, encoding a protein that is 54 amino acids longer than the canonical form [S4, S5]. The promoter also drives the expression of the short *Gm2479-201* transcript. The region encompassing the alternative transcriptional start site (TSS) as well as the exon encoding the CXXC domain of *Kdm2b* are highlighted in orange. The positions of LoxP sites in the floxed *JmjC* (referred to as *KO*) and *CxxC* conditional alleles are indicated.

  (Right) RNA expression and chromatin status along the RefSeq-annotated *Kdm2a* locus (in reverse orientation; highlighted in green) in *ctrl* and *Kdm2a* conditionally deficient oocytes. *Kdm2a* is expressed throughout oogenesis and is marked by H3K4me3 at its promoter and by H3K36me3 along its gene body. While H3K27me3 is absent, H2AK119u1 levels are increased upon oocyte growth in FGOs.
- **D.** Numbers of ovulated eggs of single and double conditionally mutant females upon hormonal superovulation treatment. Numbers of analyzed females are indicated. P-values according to two-sided student’s *t*-test.

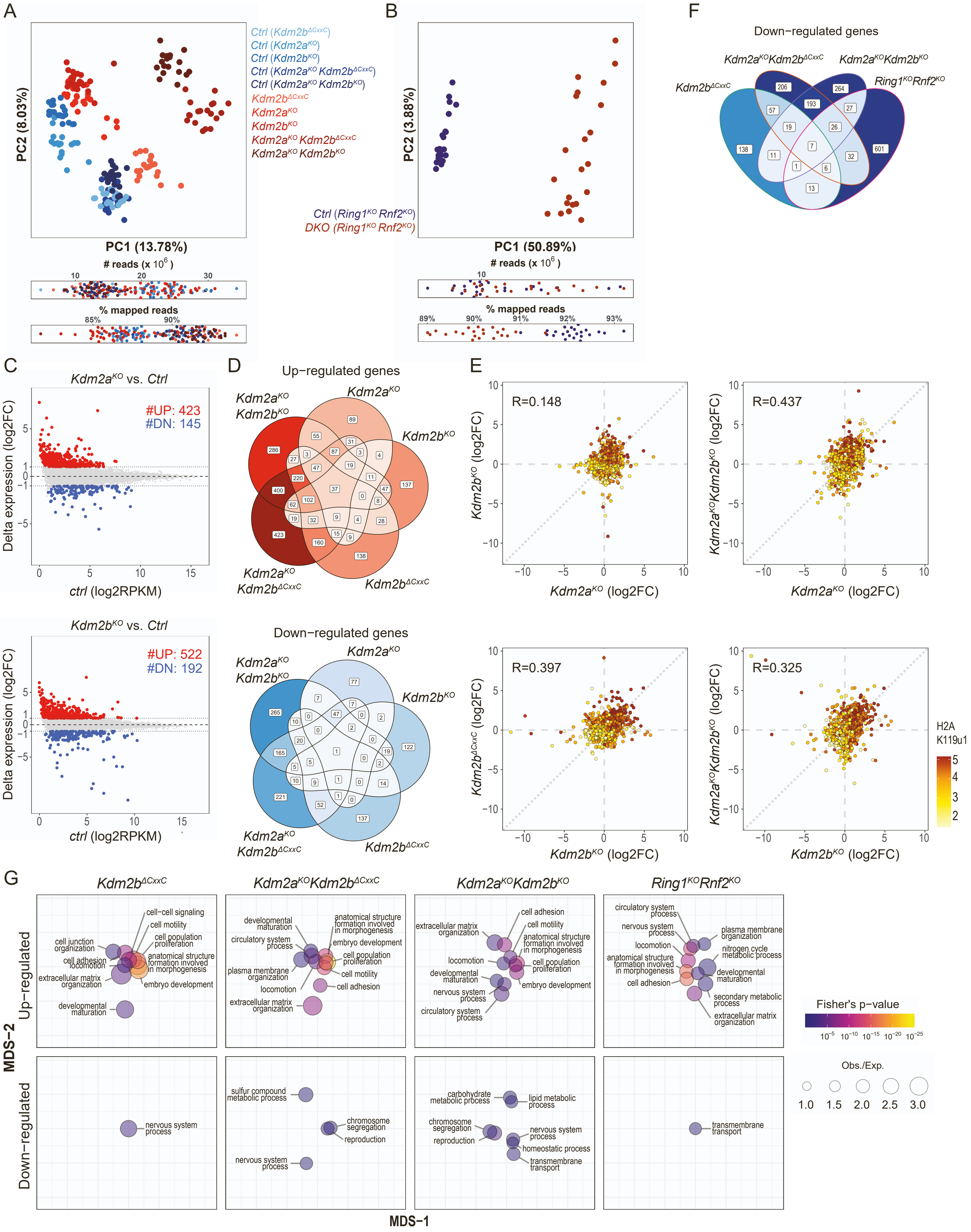

A
Ctrl (Kdm2b^ΔCxxC)
Ctrl (Kdm2a^KO)
Ctrl (Kdm2b^KO)
Ctrl (Kdm2a^KO Kdm2b^ΔCxxC)
Ctrl (Kdm2a^KO Kdm2b^KO)
Kdm2b^ΔCxxC
Kdm2a^KO
Kdm2b^KO
Kdm2a^KO Kdm2b^ΔCxxC
Kdm2a^KO Kdm2b^KO
PC2 (8.03%)
PC1 (13.78%)
reads (x 10^6)
10 20 30
% mapped reads
85% 90%
B
Ctrl (Ring1^KO Rnf2^KO)
DKO (Ring1^KO Rnf2^KO)
PC2 (3.88%)
PC1 (50.89%)
reads (x 10^6)
10
% mapped reads
89% 90% 91% 92% 93%
F
Down−regulated genes
Kdm2a^KO Kdm2b^ΔCxxC
Kdm2a^KO Kdm2b^KO
Kdm2b^ΔCxxC
Ring1^KO Rnf2^KO
206 264 57 193 27 19 26 7 138 11 32 601 1 6 13
C
Kdm2a^KO vs. Ctrl
#UP: 423
#DN: 145
Delta expression (log2FC)
ctrl (log2RPKM)
Kdm2b^KO vs. Ctrl
#UP: 522
#DN: 192
D
Up−regulated genes
Kdm2a^KO
Kdm2b^KO
Kdm2b^ΔCxxC
Kdm2a^KO Kdm2b^KO
Kdm2a^KO Kdm2b^ΔCxxC
89 55 286 31 3 3 87 4 27 47 19 220 11 37 47 137 400 0 8 102 62 19 32 9 4 28 423 15 9 160 138
Down−regulated genes
77 7 7 265 0 47 0 2 10 0 2 20 0 122 165 1 19 5 5 0 2 10 9 1 0 14 221 1 0 52 137
E
R=0.148
R=0.437
R=0.397
R=0.325
Kdm2b^KO (log2FC)
Kdm2a^KO Kdm2b^KO (log2FC)
Kdm2b^ΔCxxC (log2FC)
Kdm2a^KO (log2FC)
Kdm2b^KO (log2FC)
H2A K119u1
G
Kdm2b^ΔCxxC
Kdm2a^KO Kdm2b^ΔCxxC
Kdm2a^KO Kdm2b^KO
Ring1^KO Rnf2^KO
Up−regulated
Down−regulated
MDS−2
MDS−1
cell−cell signaling
cell motility
cell population proliferation
cell junction organization
cell adhesion
locomotion
anatomical structure formation involved in morphogenesis
extracellular matrix organization
embryo development
developmental maturation
circulatory system process
plasma membrane organization
nervous system process
nitrogen cycle metabolic process
secondary metabolic process
sulfur compound metabolic process
chromosome segregation
reproduction
carbohydrate metabolic process
lipid metabolic process
homeostatic process
transmembrane transport
Fisher's p-value
10^-5 10^-10 10^-15 10^-20 10^-25
Obs./Exp.
1.0 1.5 2.0 2.5 3.0

Figure S2: Quality control analyses of RNA-seq and CUT&RUN-seq data, related to Figure 2.

A. PCA plot illustrating the variance in RNA-seq expression data between single FGOs of indicated genotypes (*Kdm2a*^{KO}, *Kdm2b*^{KO}, *Kdm2b*$^{\Delta CxxC}$, *Kdm2a*^{KO}*Kdm2b*$^{\Delta CxxC}$, *Kdm2a*^{KO}*Kdm2b*^{KO} and respective *ctrl* FGOs). Total and mapped RNA-seq read counts of individual FGOs are indicated in panels below.

B. PCA plot illustrating the variance in RNA-seq expression data between single c*trl* and *Ring1*^{KO}*Rnf2*^{KO} FGOs. Total and mapped RNA-seq read counts of individual FGOs are indicated in panels below.

C. MA-plots showing differential expression of *Kdm2a*^{KO} or *Kdm2b*^{KO} FGOs over respective *ctrl* FGOs (log2 fold change (log2FC)) as a function of expression in respective *ctrl* FGOs (log2RPKM). #UP and #DN refer to numbers of genes more highly or lowly expressed in mutant versus *ctrl* FGOs (log2FC > 1.0; adj P-value < 0.05).

D. Venn diagrams showing numbers of genes up- or down-regulated in *Kdm2a*^{KO}, *Kdm2b*^{KO}, *Kdm2b*$^{\Delta CxxC}$, *Kdm2a*^{KO}*Kdm2b*$^{\Delta CxxC}$ and/or *Kdm2a*^{KO}*Kdm2b*^{KO} FGOs.

E. Scatter plots showing log2FC in expression of indicated mutant FGOs over respective *ctrl* FGOs versus indicated mutant FGOs over respective *ctrl* FGOs. H2AK119u1 occupancy (log2) at promoters (-1500/+500 bps of TSS) is indicated by color scale[37]. R indicates Pearson's correlation coefficient.

F. Venn diagram showing numbers of genes down-regulated in *Kdm2b*$^{\Delta CxxC}$, *Kdm2a*^{KO}*Kdm2b*$^{\Delta CxxC}$, *Kdm2a*^{KO}*Kdm2b*^{KO} and/or *Ring1*^{KO}*Rnf2*^{KO} FGOs.

G. MDS plots showing enrichments of top Gene Ontology terms for genes up- or down-regulated in *Kdm2a*^{KO}*Kdm2b*^{KO}, *Kdm2a*^{KO}*Kdm2b*$^{\Delta CxxC}$, *Kdm2b*$^{\Delta CxxC}$ and *Ring1*^{KO}*Rnf2*^{KO} FGOs over *ctrl* FGOs. Bubbles representing GO terms are scaled according to enrichments, colored according to statistical significance and positioned relative to one another to reflect similarities between significantly affected genes with corresponding GO terms.

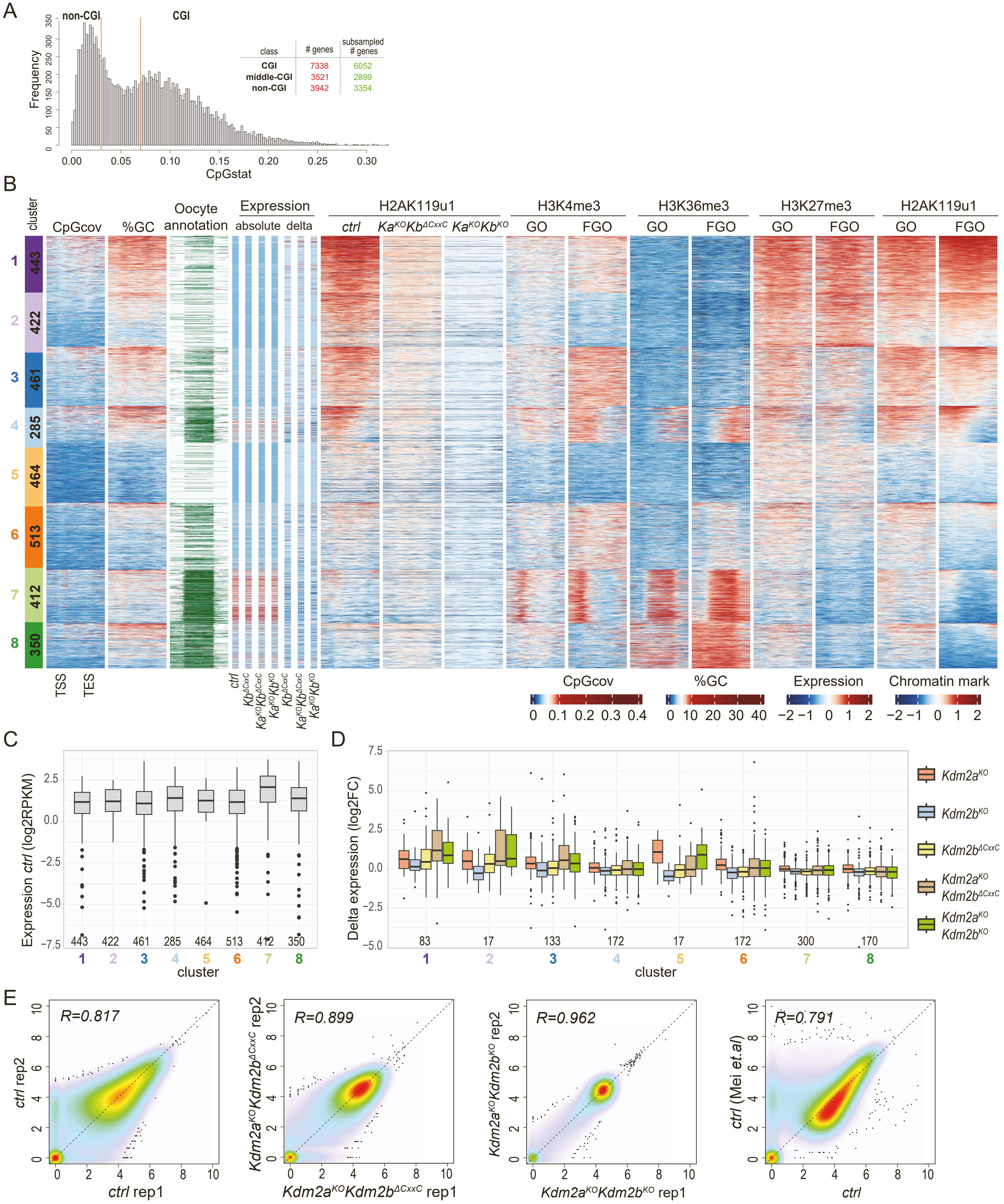

A
non-CGI
CGI
Frequency
CpGstat
class | # genes | subsampled # genes
CGI | 7338 | 6052
middle-CGI | 3521 | 2899
non-CGI | 3942 | 3354
B
cluster
CpGcov
%GC
Oocyte annotation
Expression
absolute delta
H2AK119u1
ctrl
$Ka^{KO}Kb^{\Delta CxxC}$
$Ka^{KO}Kb^{KO}$
H3K4me3
H3K36me3
H3K27me3
H2AK119u1
GO
FGO
1 443
2 422
3 461
4 285
5 464
6 513
7 412
8 350
TSS
TES
ctrl
$Kb^{\Delta CxxC}$
$Ka^{KO}Kb^{\Delta CxxC}$
$Ka^{KO}Kb^{KO}$
$Kb^{\Delta CxxC}$
$Ka^{KO}Kb^{\Delta CxxC}$
$Ka^{KO}Kb^{KO}$
CpGcov
0 0.1 0.2 0.3 0.4
%GC
0 10 20 30 40
Expression
−2 −1 0 1 2
Chromatin mark
−2 −1 0 1 2
C
Expression ctrl (log2RPKM)
443 422 461 285 464 513 412 350
cluster
D
Delta expression (log2FC)
83 17 133 172 17 172 300 170
cluster
$Kdm2a^{KO}$
$Kdm2b^{KO}$
$Kdm2b^{\Delta CxxC}$
$Kdm2a^{KO}$ $Kdm2b^{\Delta CxxC}$
$Kdm2a^{KO}$ $Kdm2b^{KO}$
E
R=0.817
ctrl rep2
ctrl rep1
R=0.899
$Kdm2a^{KO}Kdm2b^{\Delta CxxC}$ rep2
$Kdm2a^{KO}Kdm2b^{\Delta CxxC}$ rep1
R=0.962
$Kdm2a^{KO}Kdm2b^{KO}$ rep2
$Kdm2a^{KO}Kdm2b^{KO}$ rep1
R=0.791
ctrl (Mei et.al)
ctrl

Figure S3: KDM2A/KDM2B regulate H2AK119u1 deposition and gene expression during oogenesis, related to Figure 2.

- **A.** Distribution of genes according to the CpG density within the 500 bp region upstream of their transcriptional start site defined by UCSC. Genes with high and low CpG density are referred to as CGI- and nonCGI-promoter genes and have been further sub-selected for those lacking antisense expression during oogenesis. Sub-selected genes have been analyzed in subsequent figures.
- **B.** Heatmap displaying sequence composition, transcriptional and chromatin variables within nonCGI promoter genes (5kb upstream, TSS, gene body, TES, and 5 kb downstream) grouped into 8 gene clusters by k-means clustering. From left to right: gene numbers per cluster, CpG coverage; GC percentage; oocyte specific sense (green) and antisense (red) transcripts [S5]; absolute RNA (scaled RPKM) in *ctrl*, $Kdm2b^{\Delta CxxC}$, $Kdm2a^{KO}Kdm2b^{\Delta CxxC}$ and $Kdm2a^{KO}Kdm2b^{KO}$ FGOs; log2FC expression in mutant vs *ctrl* FGOs (delta); H2AK119u1 occupancy in FGOs of indicated genotypes; H3K4me3, H3K36me3, H3K27me3 and H2AK119u1 occupancies in *wt* GOs and FGOs [S6-S8]. All chromatin data are shown as Z-scores. Expression correlates with H3K4me3 promoter occupancy and H3K36me3 gene body occupancy while repression with broad H3K27me3 and H2AK119u1 occupancy in GOs.
- **C.** Boxplot presenting RNA expression levels of nonCGI promoter genes (in log2RPKM) per gene cluster in *ctrl* FGOs.
- **D.** Boxplot presenting log2FC in expression of nonCGI promoter genes measured in various mutant FGOs relative to respective *ctrl* FGOs, indicated per gene cluster.
- **E.** Reproducibility between replicates of H2AK119u1 CUT&RUN data of *ctrl*, $Kdm2a^{KO}Kdm2b^{\Delta CxxC}$ and $Kdm2a^{KO}Kdm2b^{KO}$ FGOs produced in this study. Likewise, between CUT&RUN data in *ctrl* FGOs of this study (replicates pooled) and publicly available data [S8]. Depicted are log2 transformed, library normalized counts over all 5kbp genomic tiles.

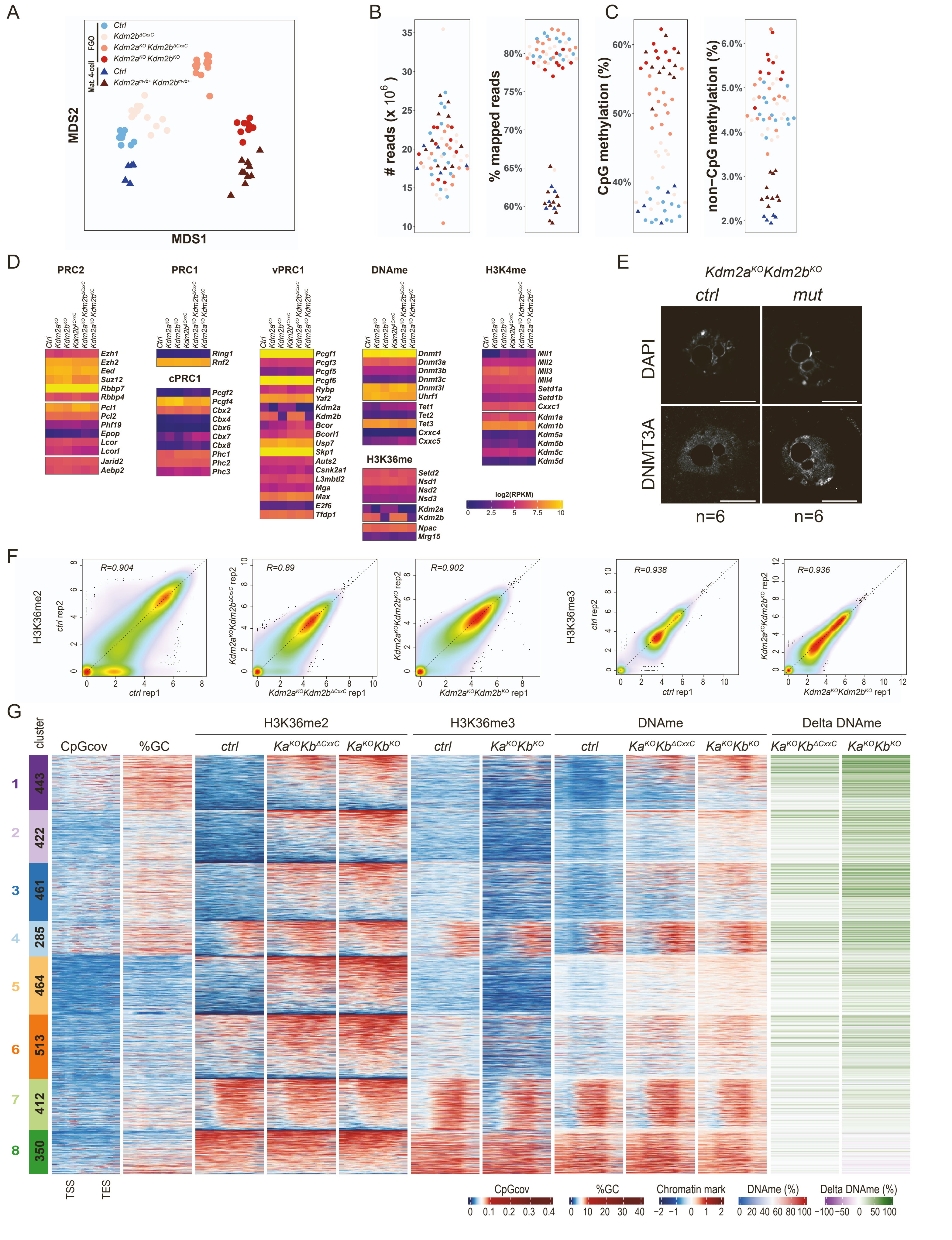

A
B
C
D
E
F
G
Ctrl
FGO
Mat. 4-cell
MDS1
MDS2
reads (x 10^6)
% mapped reads
CpG methylation (%)
non-CpG methylation (%)
PRC2
PRC1
cPRC1
vPRC1
DNAme
H3K36me
H3K4me
log2(RPKM)
Kdm2a^KO Kdm2b^KO
ctrl
mut
DAPI
DNMT3A
n=6
n=6
H3K36me2
H3K36me3
ctrl rep1
ctrl rep2
R=0.904
R=0.89
R=0.902
R=0.938
R=0.936
cluster
CpGcov
%GC
H3K36me2
H3K36me3
DNAme
Delta DNAme
TSS
TES
Chromatin mark
DNAme (%)
Delta DNAme (%)

Figure S4: Quality control analyses of WGBS and CUT&RUN-seq data, related to Figure 3.

A. MDS plot illustrating variance in WGBS data between multiple libraries generated for indicated genotypes (*Kdm2b*$^{\Delta CxxC}$, *Kdm2a*^{KO}*Kdm2b*$^{\Delta CxxC}$, *Kdm2a*^{KO}*Kdm2b*^{KO} and *ctrl* FGOs).

B. Total and mapped WGBS-seq read counts of individual libraries indicated in panel S4A.

C. Percentage CpG and non-CpG (CHG, CHH) methylation of individual libraries indicated in panel S4A.

D. RNA expression of multiple genes in *ctrl* and *Kdm2a*^{KO}*, Kdm2b*^{KO}*, Kdm2b*$^{\Delta CxxC}$, *Kdm2a*^{KO}*Kdm2b*$^{\Delta CxxC}$, *Kdm2a*^{KO}*Kdm2b*^{KO} mutant FGOs. All RNA expression data is based on polyA-primed RNA capture and Smart-seq2 library generation.

E. Representative immunofluorescence images of DNMT3A localization in *ctrl* and *Kdm2a*^{KO}*Kdm2b*^{KO} FGOs. Numbers of analyzed oocytes are indicated. Scale bars, 10 µm.

F. Reproducibility between replicates of H3K36me2 and H3K36me3 CUT&RUN data of *ctrl, Kdm2a*^{KO}*Kdm2b*$^{\Delta CxxC}$ and *Kdm2a*^{KO}*Kdm2b*^{KO} FGOs produced in this study. Depicted are log2 transformed, library normalized counts over all 5kbp genomic tiles.

G. Heatmap displaying sequence composition and chromatin variables within 8 nonCGI promoter gene clusters in FGOs, as described in Figure S3B. From left to right: gene numbers per cluster, CpG coverage; GC percentage; H3K36me2, H3K36me3 and DNAme in FGOs of indicated genotypes; differential (Delta) DNAme at nonCGI-promoters in *Kdm2a*^{KO}*Kdm2b*^{KO} and *Kdm2a*^{KO}*Kdm2b*$^{\Delta CxxC}$ FGOs.

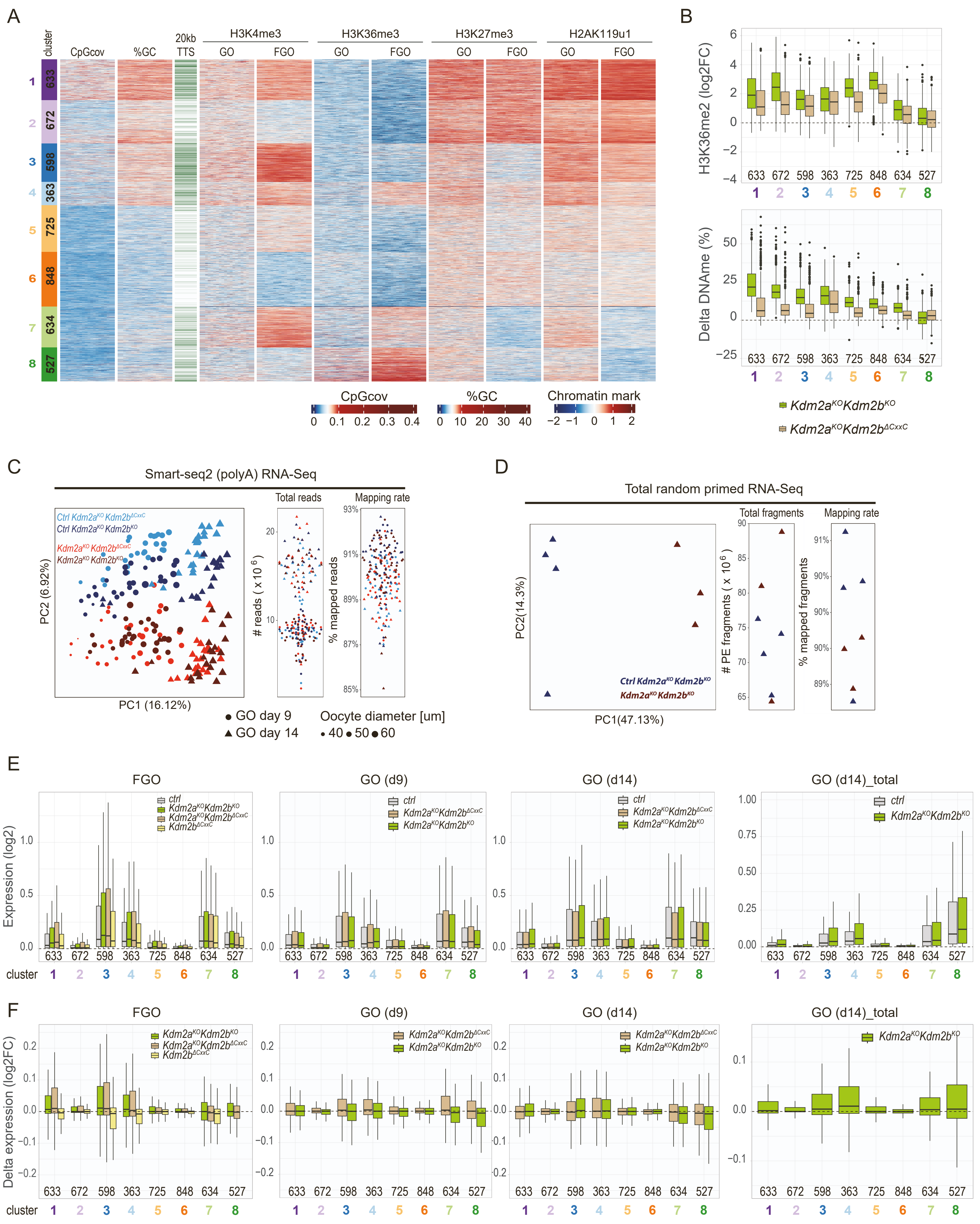

A
B
C
D
E
F
cluster
CpGcov
%GC
20kb TTS
H3K4me3
H3K36me3
H3K27me3
H2AK119u1
GO
FGO
633
672
598
363
725
848
634
527
CpGcov
%GC
Chromatin mark
H3K36me2 (log2FC)
Delta DNAme (%)
Kdm2a^KO Kdm2b^KO
Kdm2a^KO Kdm2b^ΔCxxC
Smart-seq2 (polyA) RNA-Seq
Total reads
Mapping rate
Ctrl Kdm2a^KO Kdm2b^ΔCxxC
Ctrl Kdm2a^KO Kdm2b^KO
Kdm2a^KO Kdm2b^ΔCxxC
Kdm2a^KO Kdm2b^KO
PC2 (6.92%)
PC1 (16.12%)
reads (x10^6)
% mapped reads
GO day 9
GO day 14
Oocyte diameter [um]
40 50 60
Total random primed RNA-Seq
Total fragments
Mapping rate
PC2(14.3%)
PC1(47.13%)
# PE fragments (x 10^6)
% mapped fragments
FGO
GO (d9)
GO (d14)
GO (d14)_total
ctrl
Kdm2b^ΔCxxC
Expression (log2)
Delta expression (log2FC)

Figure S5: H3K36me2 and DNAme accumulate throughout the genome of *Kdm2a*/*Kdm2b* mutant oocytes, independently of transcription, related to Figure 4.

A. Heatmap displaying sequence composition and chromatin variables within 8 clusters of 10 kb intergenic regions (20 neighboring 500 bp bins) in oocytes. From left to right: number of regions per cluster, CpG coverage; GC percentage; presence of annotated TTS in 20 kb flanking regions, that could be compatible with run-through transcription through the window; H3K4me3, H3K36me3, H3K27me3 and H2AK119u1 occupancy in *wt* GOs and FGOs [S6-S9].

B. Boxplots displaying differential H3K36me2 and DNAme for 10 kb intergenic regions in 8 clusters in $Kdm2a^{KO}Kdm2b^{KO}$ or $Kdm2a^{KO}Kdm2b^{\Delta CxxC}$ FGOs relative to *ctrl* FGOs, as indicated. Numbers of regions per cluster are indicated.

C. PCA plot illustrating variance in smart-seq2 polyA-based RNA-seq expression data between single GOs isolated at day 9 and 14 of indicated genotypes ($Kdm2a^{KO}Kdm2b^{\Delta CxxC}$, $Kdm2a^{KO}Kdm2b^{KO}$ and respective *ctrl* FGOs). Oocyte diameter, total and mapped RNA-seq read counts of individual GOs are also indicated.

D. PCA plot illustrating variance in total random-primed RNA-seq expression data between libraries prepared of pooled $Kdm2a^{KO}Kdm2b^{KO}$ and *ctrl* GOs isolated at day 14 of development. Total and mapped RNA-seq fragment counts per library are indicated as well.

E. Boxplots displaying absolute expression for 10 kb intergenic regions in 8 clusters in *ctrl* and $Kdm2b^{\Delta CxxC}$, $Kdm2a^{KO}Kdm2b^{\Delta CxxC}$ and $Kdm2a^{KO}Kdm2b^{KO}$ mutant GOs isolated at day9 and day14, and in FGOs. Numbers of regions per cluster are indicated. RNA expression data are based on polyA-primed RNA capture and Smart-seq2 library generation of single oocytes or on random primed (total) RNA capture of pools of day14 GOs as indicated.

F. Boxplots displaying log2FC expression for 10 kb intergenic regions in 8 clusters in $Kdm2b^{\Delta CxxC}$, $Kdm2a^{KO}Kdm2b^{\Delta CxxC}$ and $Kdm2a^{KO}Kdm2b^{KO}$ samples relative to *ctrl* samples as shown in panel S5E.

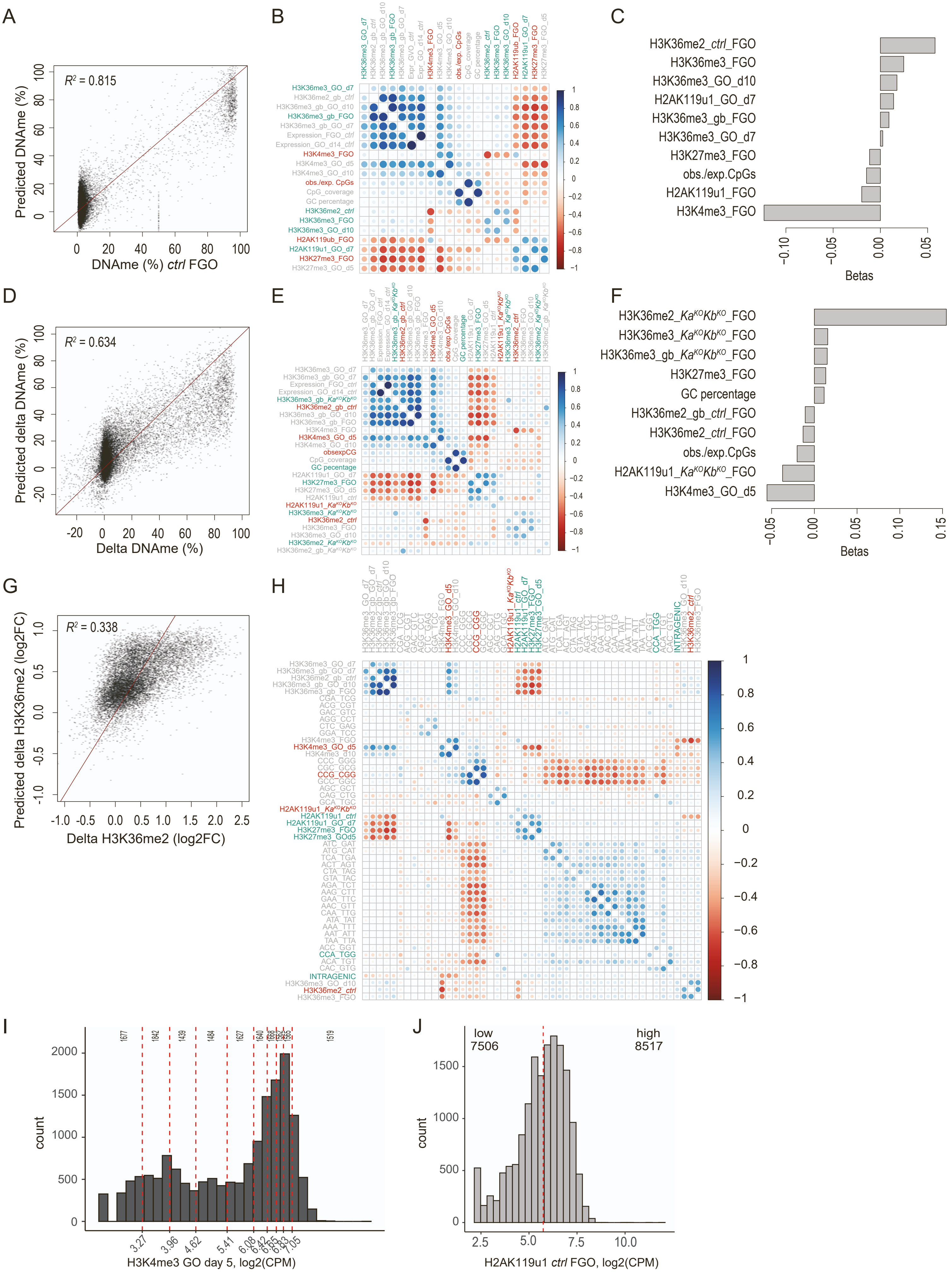

A
R^2 = 0.815
Predicted DNAme (%)
DNAme (%) ctrl FGO
B
C
H3K36me2_ctrl_FGO
H3K36me3_FGO
H3K36me3_GO_d10
H2AK119u1_GO_d7
H3K36me3_gb_FGO
H3K36me3_GO_d7
H3K27me3_FGO
obs./exp.CpGs
H2AK119u1_FGO
H3K4me3_FGO
Betas
D
R^2 = 0.634
Predicted delta DNAme (%)
Delta DNAme (%)
E
F
H3K36me2_KaKOKbKO_FGO
H3K36me3_KaKOKbKO_FGO
H3K36me3_gb_KaKOKbKO_FGO
H3K27me3_FGO
GC percentage
H3K36me2_gb_ctrl_FGO
H3K36me2_ctrl_FGO
obs./exp.CpGs
H2AK119u1_KaKOKbKO_FGO
H3K4me3_GO_d5
Betas
G
R^2 = 0.338
Predicted delta H3K36me2 (log2FC)
Delta H3K36me2 (log2FC)
H
I
count
H3K4me3 GO day 5, log2(CPM)
J
low 7506
high 8517
count
H2AK119u1 ctrl FGO, log2(CPM)

Figure S6: Identifying chromatin and sequence features underlying aberrant H3K36me2 and DNAme acquisition in *Kdm2a*/*Kdm2b* mutant oocytes by regularized linear regression analysis, related to Figure 5.

A. Scatter plot showing DNAme (%) at promoter and intragenic CGIs measured in *ctrl* FGOs versus DNAme (%) at CGIs as predicted as predicted by regularized linear regression analysis using chromatin states in *ctrl* oocytes. R^2 = 0.815. Line represents diagonal.

B. Dotplot diagram presenting the correlations between sequence, expression and chromatin features at CGIs measured in GOs and FGOs as indicated by the blue to red color gradient. Green and red labeled parameters contribute positively or negatively to the DNAme predictions as described in Figure S6A. Abbreviations: gb = signal in gene body; obs./exp. CpGs = observed over expected CpGs in CGIs; d7, d10: postnatal day 7 or 10.

C. Barplot showing the *beta* coefficients of the top 10 variables contributing to the prediction of DNAme status at CGIs in *ctrl* FGOs as described in Figure S6A.

D. Scatter plot showing the difference in DNAme (%) at promoter and intragenic CGIs in $Kdm2a^{KO}Kdm2b^{KO}$ over *ctrl* FGOs versus the difference in DNAme (%) between genotypes as predicted by regularized linear regression analysis using chromatin states in *ctrl* and $Kdm2a^{KO}Kdm2b^{KO}$ oocytes. R^2 = 0.634. Line represents diagonal.

E. Dotplot diagram presenting the correlations between sequence, expression and chromatin features at CGIs measured in GOs and FGOs as indicated by the blue to red color gradient. Green and red labeled parameters contribute positively or negatively to the DNAme predictions as described in Figure S6D. Abbreviations: gb = signal in gene body; obs./expt. CpGs = observed over expected CpGs in CGIs; d5: postnatal day 5.

F. Barplot showing the *beta* coefficients of the top 10 variables contributing to the prediction of differential DNAme at CGIs in $Kdm2a^{KO}Kdm2b^{KO}$ over *ctrl* FGOs as described in Figure S6D.

G. Scatter plot showing the difference in H3K36me2 occupancy at promoter and intragenic CGIs in $Kdm2a^{KO}Kdm2b^{KO}$ FGOs over *ctrl* FGOs versus the difference in H3K36me2 occupancy between genotypes as predicted by regularized linear regression analysis using chromatin states in *ctrl* and $Kdm2a^{KO}Kdm2b^{KO}$ oocytes and trinucleotide sequences. R^2 = 0.338. Line represents diagonal.

H. Dotplot diagram presenting the correlations between sequence, expression and chromatin features at CGIs measured in growing and FGOs as indicated by the blue to red color gradient. Green and red labeled parameters contribute positively or negatively to the H3K36me2 predictions as described in Figure S6G. Abbreviations: gb = signal in gene body; obs./expt. CpGs = observed over expected CpGs in CGIs; Trinucleotide frequencies variables are encoded as FFF_RRR pairs where RRR is the reverse complement of FFF.

I. Histogram displaying distribution of H3K4me3 occupancy levels at 533 bp-regions surrounding 16’023 CGIs in GOs. Classification of CGIs into 10 bins with approximately equal numbers of CGIs is indicated.

J. Histogram displaying distribution of H2AK119u1 occupancy levels at 533 bp-regions surrounding 16’023 CGIs in GOs. CGIs classified as having low or high H2K119u1 levels are indicated.

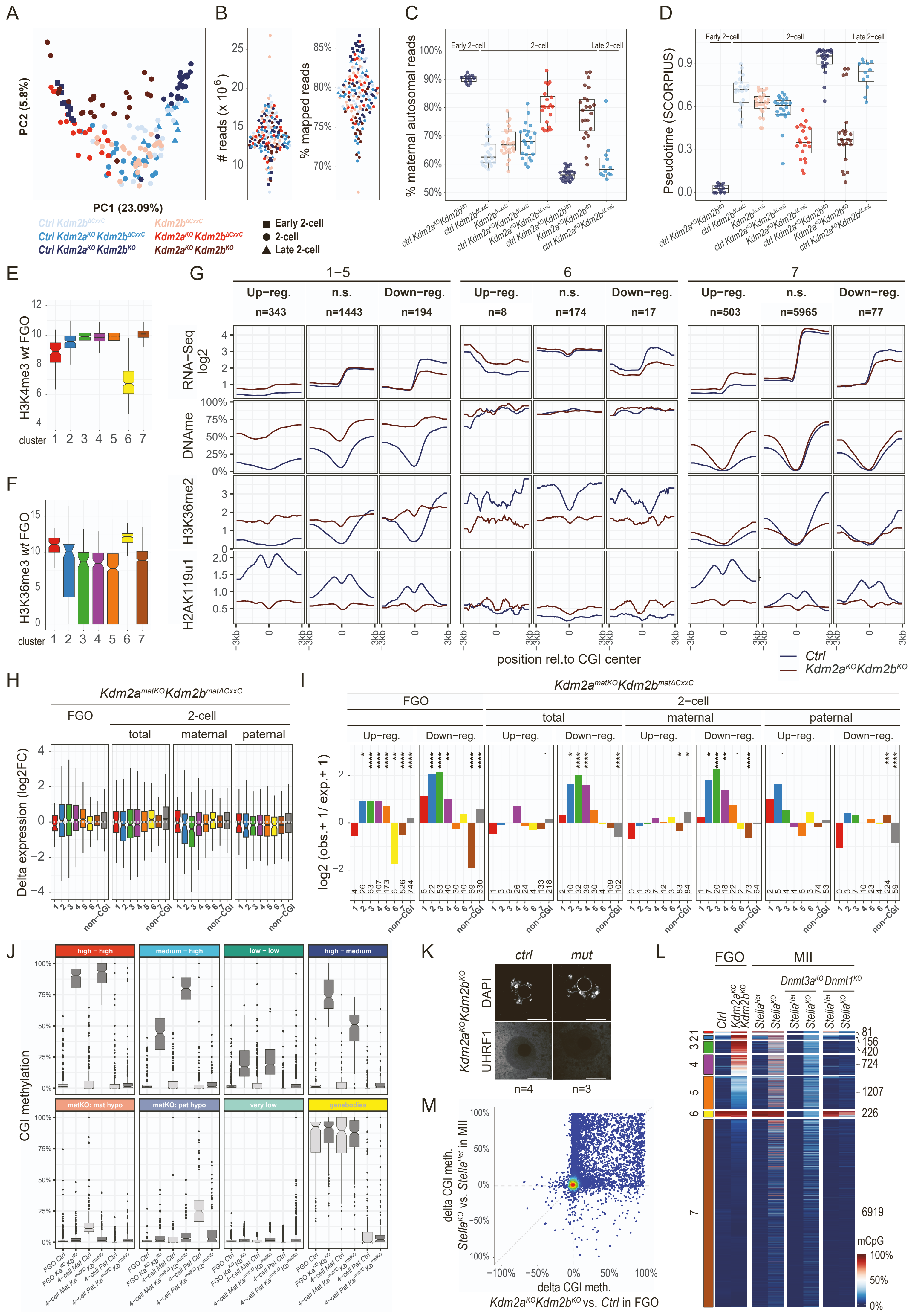
A
PC1 (23.09%)
PC2 (5.8%)
Ctrl Kdm2bΔCxxC
Kdm2bΔCxxC
Ctrl Kdm2aKO Kdm2bΔCxxC
Kdm2aKO Kdm2bΔCxxC
Ctrl Kdm2aKO Kdm2bKO
Kdm2aKO Kdm2bKO
Early 2-cell
2-cell
Late 2-cell
B
reads (x 10^6)
% mapped reads
C
% maternal autosomal reads
Early 2-cell
2-cell
Late 2-cell
D
Pseudotime (SCORPIUS)
E
H3K4me3 wt FGO
cluster
F
H3K36me3 wt FGO
G
1–5
6
7
Up-reg.
n.s.
Down-reg.
n=343
n=1443
n=194
n=8
n=174
n=17
n=503
n=5965
n=77
RNA-Seq log2
DNAme
H3K36me2
H2AK119u1
position rel.to CGI center
Ctrl
Kdm2aKOKdm2bKO
H
Kdm2amatKOKdm2bmatΔCxxC
FGO
2-cell
total
maternal
paternal
Delta expression (log2FC)
non-CGI
I
log2 (obs.+ 1/ exp.+ 1)
J
high – high
medium – high
low – low
high – medium
matKO: mat hypo
matKO: pat hypo
very low
genebodies
CGI methylation
K
ctrl
mut
DAPI
UHRF1
Kdm2aKOKdm2bKO
n=4
n=3
L
FGO
MII
Dnmt3aKO Dnmt1KO
Ctrl
Kdm2aKO Kdm2bKO
StellaHet
StellaKO
mCpG
M
delta CGI meth. StellaKO vs. StellaHet in MII
delta CGI meth. Kdm2aKOKdm2bKO vs. Ctrl in FGO

Figure S7: RNA and chromatin dynamics in *Kdm2a/Kdm2b* mutant versus *ctrl* FGOs at UCSC-defined CGIs associated with genes being up- or down-regulated in 2-cell embryos, related to Figure 7.

A. PCA plot illustrating variance in RNA-seq expression data between single 2-cell embryos of indicated genotypes (*Kdm2b*$^{\Delta CxxC}$, *Kdm2a*^{KO}*Kdm2b*$^{\Delta CxxC}$, *Kdm2a*^{KO}*Kdm2b*^{KO}, and respective *ctrl*) and stage (early 2-cell, 2-cell and late 2-cell stage).

B. RNA-seq read counts and mapping rates for individual 2-cell embryos in Figure S7A.

C. Percentage of SNP based allelic reads attributed as transcribed from the maternal genome for single embryos grouped by stage and genotype.

D. Inferred pseudotime for single embryos grouped by stage and genotype.

E. **F.** Boxplots displaying log2 enrichment for H3K4me3 [S7] (**E**) and H3K36me3 [S6] (**F**) in *wt* FGOs at promoter CGIs according to UCSC for genes belonging to the different DNAme clusters, as defined in Figure 7A.

G. Metaprofiles for RNA-seq read coverage (including coverage from exon-exon junctions in spliced reads), mCpG methylation, enrichments for H3K36me2 and H2AK119ub1 in *Ctrl* and *Kdm2a*^{KO}*Kdm2b*^{KO} FGOs around promoter CGIs belonging to clusters 1-5, 6 and 7 in Figure 7A nearby up-regulated, not significantly changed (n.s.) and down-regulated genes in *Kdm2a*^{KO}*Kdm2b*^{KO} FGOs. Direction from negative to positive positions around CGI centers coincide with direction from promoters to gene bodies of nearby genes.

H. Boxplot showing log2FC expression of different clusters of CGI-promoter and all nonCGI promoter genes in *Kdm2a*^{KO}*Kdm2b*$^{\Delta CxxC}$ over *ctrl* FGOs and in *Kdm2a*^{matKO}*Kdm2b*$^{mat\Delta CxxC}$ over *ctrl* 2-cell embryos according to all, mat and pat specific sequencing reads.

I. Barplot showing over-/under-representation and statistical significance of CGI-promoter genes belonging to the different DNAme clusters and being either significantly up- or down-regulated in *Kdm2a*^{KO}*Kdm2b*$^{\Delta CxxC}$ relative to *ctrl* FGOs or in *Kdm2a*^{matKO}*Kdm2b*$^{mat\Delta CxxC}$ relative to *ctrl* 2-cell embryos for all, mat and pat specific sequencing reads. Numbers of affected genes are indicated below the bars. Statistical significance is coded as follows: **** ≤ 0.001%; *** ≤ 0.01%; ** ≤ 0.1%; * ≤ 1%; · ≤ 5%.

J. Box plots showing DNAme levels at CGI-promoters, classified in 8 groups as shown in Figure 7F, in *ctrl* and *Kdm2a*^{KO}*Kdm2b*^{KO} FGOs, and in *ctrl* and *Kdm2a*^{matKO}*Kdm2b*^{matKO} 4-cell embryos for maternal and paternal genomes.

K. Representative immunofluorescence images of UHRF1 localization in *ctrl* and *Kdm2a*^{KO}*Kdm2b*^{KO} FGOs. Numbers of analyzed oocytes are indicated. Scale bars, 10 µm.

L. Heatmap showing absolute DNAme levels at CGI-gene promoters in *ctrl* and *Kdm2a*^{KO}*Kdm2b*^{KO} FGOs that had been clustered as described in panel 7A. DNAme levels at corresponding CGIs in MII-oocytes being heterozygous or deficient for *Stella* and wt or deficient for *Dnmt3a* or *Dnmt1*, as measured by RRBS, are shown as well [S10].

M. Scatter plot showing differences in DNAme at CGI-promoters in *Kdm2a*^{KO}*Kdm2b*^{KO} versus *ctrl* FGOs over *Stella*^{KO} versus *Stella*^{Het} MII oocytes.

Supplemental references

[S1]. Wang, C., Liu, X., Gao, Y., Yang, L., Li, C., Liu, W., Chen, C., Kou, X., Zhao, Y., Chen, J., et al. (2018). Reprogramming of H3K9me3-dependent heterochromatin during mammalian embryo development. Nature Cell Biology *20*. 10.1038/s41556-018-0093-4.

[S2]. De Vries, W.N., Binns, L.T., Fancher, K.S., Dean, J., Moore, R., Kemler, R., and Knowles, B.B. (2000). Expression of Cre recombinase in mouse oocytes: A means to study maternal effect genes. Genesis *26*. 10.1002/(SICI)1526-968X(200002)26:2<110::AID-GENE2>3.0.CO;2-8.

[S3]. Turberfield, A.H., Kondo, T., Nakayama, M., Koseki, Y., King, H.W., Koseki, sH., and Klose, R.J. (2019). KDM2 proteins constrain transcription from CpG island gene promoters independently of their histone demethylase activity. Nucleic Acids Research *47*. 10.1093/NAR/GKZ607.

[S4]. Blackledge, N.P., Farcas, A.M., Kondo, T., King, H.W., McGouran, J.F., Hanssen, L.L.P., Ito, S., Cooper, S., Kondo, K., Koseki, Y., et al. (2014). Variant PRC1 complex-dependent H2A ubiquitylation drives PRC2 recruitment and polycomb domain formation. Cell *157*. 10.1016/j.cell.2014.05.004.

[S5]. Veselovska, L., Smallwood, S.A., Saadeh, H., Stewart, K.R., Krueger, F., Maupetit-Méhouas, S., Arnaud, P., Tomizawa, S.i., Andrews, S., and Kelsey, G. (2015). Erratum to: Deep sequencing and de novo assembly of the mouse occyte transcriptome define the contribution of transcription to the DNA methylation landscape[Genome Biol., 16, (2015), 209]. Genome Biology. 10.1186/s13059-015-0809-8.

[S6]. Xu, Q., Xiang, Y., Wang, Q., Wang, L., Brind'Amour, J., Bogutz, A.B., Zhang, Y., Zhang, B., Yu, G., Xia, W., et al. (2019). SETD2 regulates the maternal epigenome, genomic imprinting and embryonic development. Nature Genetics *51*. 10.1038/s41588-019-0398-7.

[S7]. Hanna, C.W., Taudt, A., Huang, J., Gahurova, L., Kranz, A., Andrews, S., Dean, W., Stewart, A.F., Colomé-Tatché, M., and Kelsey, G. (2018). MLL2 conveys transcription-independent H3K4 trimethylation in oocytes. Nature Structural and Molecular Biology *25*. 10.1038/s41594-017-0013-5.

[S8]. Mei, H., Kozuka, C., Hayashi, R., Kumon, M., Koseki, H., and Inoue, A. (2021). H2AK119ub1 guides maternal inheritance and zygotic deposition of H3K27me3 in mouse embryos. Nature Genetics *53*. 10.1038/s41588-021-00820-3.

[S9]. Bourc'his, D., and Bestor, T.H. (2004). Meiotic catastrophe and retrotransposon reactivation in male germ cells lacking Dnmt3L. Nature. 10.1038/nature02886.

[S10]. Li, Y., Zhang, Z., Chen, J., Liu, W., Lai, W., Liu, B., Li, X., Liu, L., Xu, S., Dong, Q., et al. (2018). Stella safeguards the oocyte methylome by preventing de novo methylation mediated by DNMT1. Nature *564*. 10.1038/s41586-018-0751-5.
