# Supplementary material for: Preventing CpG hypermethylation in oocytes safeguards mouse development
Source: Dev Cell. 2025 Dec 1;60(23):3285–3303.e9. doi: 10.1016/j.devcel.2025.08.005 (PMC12687725; doi:10.1016/j.devcel.2025.08.005)
Supplement: Document S2. Article plus supplemental information [file mmc9.pdf]

Article

Developmental Cell

Preventing CpG hypermethylation in oocytes safeguards mouse development

Graphical abstract

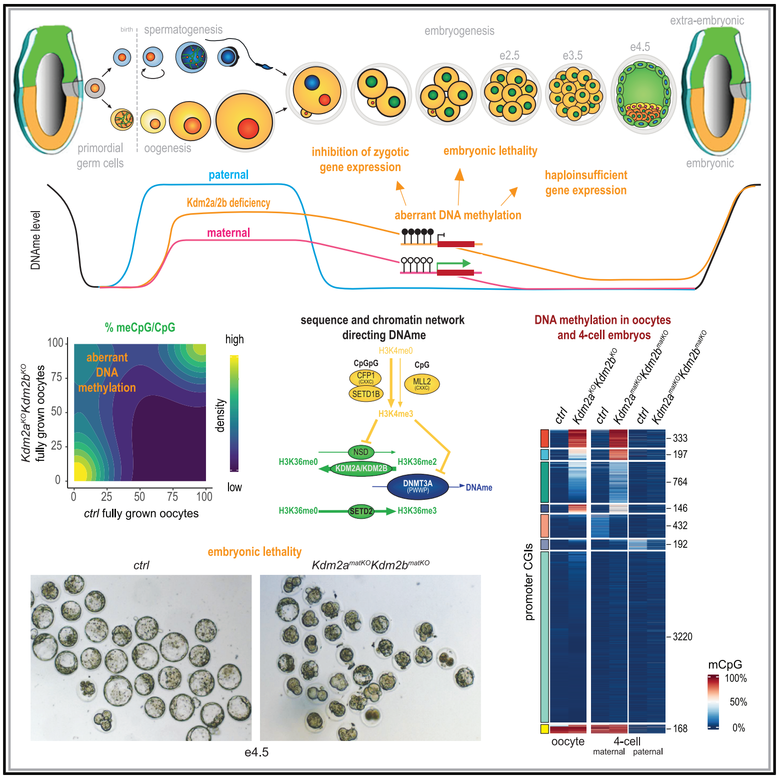

Highlights

- KDM2A/KDM2B prevent *de novo* DNA methylation in mouse oocytes by demethylating H3K36me2
- DNA sequence and H3K4me3 control aberrant H3K36me2 and DNAme acquisition at CGIs
- Aberrant oocyte DNAme is not reprogrammed in early embryos and represses transcription
- Intergenerational inheritance of aberrant oocyte DNAme is lethal for early embryos

Authors

Yumiko K. Kawamura, Evgeniy A. Ozonov, Panagiotis Papasaikas, ..., Sebastien A. Smallwood, Haruhiko Koseki, Antoine H.F. M. Peters

Correspondence

antoine.peters@fmi.ch

In brief

Kawamura et al. show that H3K36me2 removal by the KDM2A and KDM2B histone demethylases restricts DNMT3A in establishing global DNA methylation in oocytes. Maternal *Kdm2a/Kdm2b* knockout embryos die during pre-implantation development with impaired gene transcription, identifying limited DNA methylation reprogramming and susceptibility to gene dosage haplo-insufficiency effects in early mouse embryos.

Kawamura et al., 2025, Developmental Cell *60*, 3285–3303
December 1, 2025 © 2025 The Author(s). Published by Elsevier Inc.
https://doi.org/10.1016/j.devcel.2025.08.005

Developmental Cell

CellPress
OPEN ACCESS

Article

Preventing CpG hypermethylation in oocytes safeguards mouse development

Yumiko K. Kawamura,[1] Evgeniy A. Ozonov,[1,2,5] Panagiotis Papasaikas,[1,2,5] Takashi Kondo,[3] Nhuong V. Nguyen,[1] Michael B. Stadler,[1,2] Sebastien A. Smallwood,[1] Haruhiko Koseki,[3] and Antoine H.F.M. Peters[1,4,6,*]
[1]Friedrich Miescher Institute for Biomedical Research, 4056 Basel, Switzerland
[2]SIB Swiss Institute of Bioinformatics, 4056 Basel, Switzerland
[3]Laboratory for Developmental Genetics, RIKEN Center for Integrative Medical Sciences (IMS), Yokohama 230-0045, Japan
[4]Faculty of Sciences, University of Basel, 4056 Basel, Switzerland
[5]These authors contributed equally
[6]Lead contact
*Correspondence: antoine.peters@fmi.ch
https://doi.org/10.1016/j.devcel.2025.08.005

SUMMARY

Except for regulatory CpG-island sequences, genomes of most mammalian cells are widely DNA-methylated. In oocytes, though, DNA methylation (DNAme) is largely confined to transcribed regions. The mechanisms restricting *de novo* DNAme in oocytes and their relevance thereof for zygotic genome activation and embryonic development are largely unknown. Here we show that KDM2A and KDM2B, two histone demethylases, prevent genome-wide accumulation of histone H3 lysine 36 di-methylation, thereby impeding DNMT3A-catalyzed DNAme. We demonstrate that aberrant DNAme at CpG islands inherited from *Kdm2a/Kdm2b* double-mutant oocytes represses gene transcription in two-cell embryos. Aberrant maternal DNAme impairs pre-implantation embryonic development, which is suppressed by *Dnmt3a* deficiency during oogenesis. Hence, KDM2A/KDM2B are essential for confining the oocyte methylome, thereby conferring competence for early embryonic development. Our research implies that the reprogramming capacity eminent to early embryos is insufficient for erasing aberrant DNAme from maternal chromatin, and that early development is susceptible to gene dosage haplo-insufficiency effects.

INTRODUCTION

Shortly after fertilization, genomes undergo extensive reprogramming of germline-specific epigenetic programs, including DNA methylation and chromatin, to support acquisition of totipotency and embryonic development. DNA methylation in mammals is generally found on cytosine residues within CpG dinucleotides (mCpG) throughout the genome, while CpGs located within CpG dinucleotide-dense regions, commonly referred to as CpG islands (CGIs), are generally unmethylated throughout the entire mammalian life cycle, including in sperm and oocytes. Many CGIs serve transcriptional regulatory functions at housekeeping and cell-fate-determining genes.[1] Through evolution, CpGs have become underrepresented in mammalian genomes due to deamination and incorrect repair of methylated cytosines in the germ line.[2] To date, the mechanisms ensuring the unmethylated status of CGIs in the germ line remain poorly understood. It is further unknown whether the germline-derived unmethylated state of CGIs in parental genomes is required for zygotic genome activation in early embryos and for supporting embryonic development, or whether (experimentally induced) DNAme at CGIs would undergo epigenetic reprogramming after fertilization.

Within the mammalian life cycle, genomes undergo two rounds of erasure and re-establishment of global DNAme patterns, largely excluding CGIs.[3] Following the specification of primordial germ cells, genomes first lose embryonically established DNAme and then acquire in a sexually dimorphic manner oocyte- and sperm-specific DNAme patterns supporting germline-specific cellular physiology. Next, following fertilization, both genomes lose most DNAme in a parent-of-origin-specific manner.[4] After implantation, parental genomes become similarly and widely methylated. Despite extensive DNAme reprogramming during pre-implantation development, some regions on maternal and paternal genomes escape erasure.[5,6] The transmission of DNAme at so-called imprinting control regions (ICRs) drives parent-of-origin-specific mono-allelic repression, which is vital to embryo development.[3]

Remarkably, global patterns and functions of DNAme differ greatly between sperm and oocytes. Male germ cells gain DNAme at >90% of individual CpGs, comparable to somatic cells. Such DNAme is essential for meiotic progression[7] and maintenance of long-term spermatogenesis.[8] By contrast, growing oocytes (GOs) acquire high *de novo* DNAme levels exclusively in transcribed regions and low levels in other regions, resulting in global DNAme levels <40%.[9–11] Curiously, DNAme in oocytes does not majorly regulate gene expression, nor is it required for oocyte development.[12] After fertilization, however, embryos lacking maternal DNAme arrest by day 10.5 of

This is an open access article under the CC BY license (http://creativecommons.org/licenses/by/4.0/).

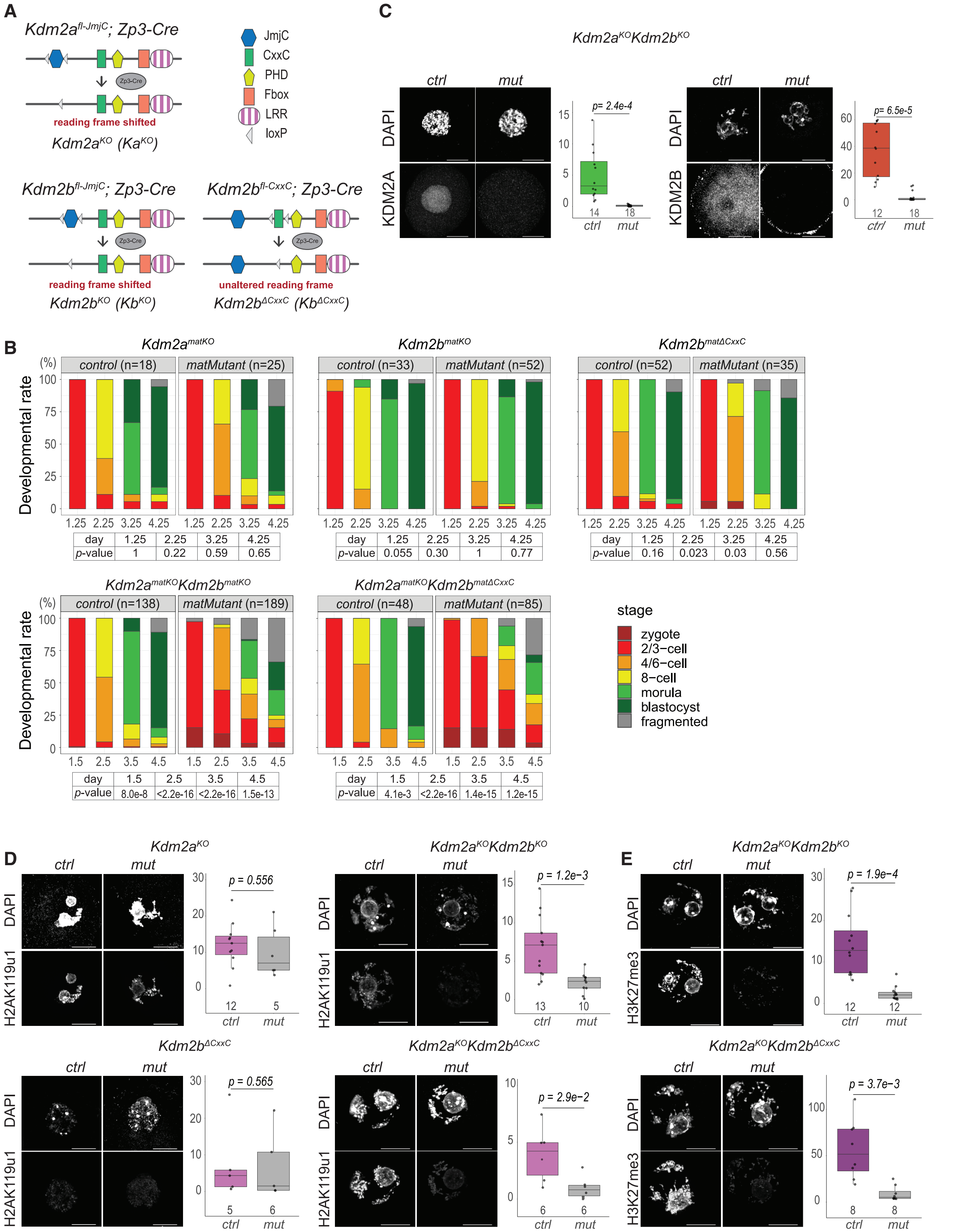

Figure 1. KDM2A and KDM2B function in oocytes to regulate embryonic development

(A) Schematic overview of *Kdm2a* and *Kdm2b* genes expressed in oocytes of *ctrl* and mutant conditions. Positions of JmjC- and CxxC-encoding domains flanked by *loxP* sites are indicated.[29,31]

(legend continued on next page)

gestation due to genomic imprinting defects and/or impaired trophoblast formation.[12,13]

As in somatic cells, *de novo* DNAme acquisition in germ cells is controlled by histone methylation modifiers.[14–18] In GOs, most DNAme catalyzed by the *de novo* DNA methyltransferase DNMT3A is directed by transcription-coupled histone H3 lysine 36 trimethylation (H3K36me3) deposited by SETD2.[9,19,20] Moderate-to-low DNAme has been associated with H3K36me2 occupancy.[11] Inversely, DNMT3A catalysis is inhibited by H3K4me3, which is widely deposited in GOs by the SETD1B and MLL2 enzymes.[21–24] At selective regions, including ICRs, H3K4me3 is removed by the KDM1B demethylase, enabling DNAme acquisition.[22] In mouse embryonic stem cells (ESCs), loss of KDM2B expression (also termed FBXL10, NDY1, JHDM1B, and CXXC2) resulted in aberrant DNAme at CGIs controlled by Polycomb repressive complexes (PRCs).[25] The mechanism underlying such selective DNAme acquisition has, however, remained unknown.[25] Importantly, KDM2B localizes at almost all CGIs throughout the mouse genome via recognition of unmethylated CpGs by its CXXC domain.[26] The protein also contains a JmjC domain that was reported to demethylate H3K36me2 *in vitro*.[27,28] In ESCs, however, only limited activity was reported.[29] KDM2B further contains a plant homeodomain domain, an F-box domain, and a leucine-rich repeat that interacts with members of the variant PRC1.1 (vPRC1.1).[30–32] vPRC1.1 deposits histone H2AK119 mono-ubiquitin (H2AK119u1) at CGIs through the E3 ligases RING1 and RNF2, which are PRC1 core components, and is essential for transcriptional repression of target genes.[29,33] Like KDM2B, the KDM2A paralog (FBXL11, JHDM1A, and CXXC8) localizes at unmethylated CGIs[34] and demethylates H3K36me2 *in vitro*.[28] KDM2A plays, however, only a minor gene regulatory function in ESCs, compared with KDM2B.[29] Oocyte-specific *Kdm2a* deficiency was reported to compromise oogenesis.[35]

We previously identified *Ring1* and *Rnf2* as critical transcriptional regulators and chromatin modifiers in oocytes, essential for defining embryonic competence.[36] Deficiency for *Pcgf1*, another component of vPRC1.1, indicated a role for this complex in defining H2AK119u1 and transcriptional states in oocytes.[37] Here, we study the role of *Kdm2b* and its paralog *Kdm2a* in regulating PRC1-mediated gene repression and *de novo* DNAme acquisition during oogenesis and the impact of their loss of function on embryogenesis.[28,34] We identify KDM2A and KDM2B as essential maternal regulators of pre-implantation development, safeguarding the maternal genome against CpG hypermethylation throughout the genome, including CGIs, thereby enabling proper zygotic gene expression and embryo viability.

RESULTS

Kdm2a/Kdm2b function in oocytes controls embryogenesis

RNA sequencing experiments show that *Kdm2b* and other vPRC1 components are highly expressed in GOs and fully grown germinal vesicle oocytes (FGOs) and in early embryos (Figure S1A). To study vPRC1.1 function in oocytes and pre-implantation embryos, we conditionally altered expression of *Kdm2b* in GOs in two ways using the *Zp3*-promoter-driven CRE-recombinase expressed in primary GOs (Figure S1B). Firstly, removal of exons encoding the histone demethylase JmjC domain (*Kdm2b*$^{fl\text{-}JmjC}$)[29] abrogated *Kdm2b* expression completely (Figure S1C). We therefore designate the *Kdm2b*$^{fl\text{-}JmjC}$ allele as a knockout (*Kdm2b*^{KO}) allele (Figure 1A). Secondly, we generated mice expressing a KDM2B protein lacking its CxxC domain (*Kdm2b*$^{\Delta CxxC}$; Figures 1A and S1C). In ESCs, this domain was shown to recruit vPRC1.1 to CGIs.[31] In both models, we did not observe changes in the number of ovulated oocytes nor in the progression of pre-implantation embryonic development after *in vitro* fertilization with wild-type (WT) sperm generating so-called *Kdm2b*^{matKO} or *Kdm2b*$^{mat\Delta CxxC}$ embryos (Figures 1B and S1D).

To address developmental roles of the KDM2A paralog, highly expressed in oocytes and therefore possibly compensating for the *Kdm2b* deficiency, we generated oocytes conditionally deficient for *Kdm2a* transcript and protein expression, alone and in combination with either *Kdm2b* mutation (Figures 1A, S1A, and S1C). Whereas the development of *Kdm2a*^{KO} oocytes and resulting *Kdm2a*^{matKO} embryos was not affected, double *Kdm2a*^{KO}*Kdm2b*^{KO} deficiency in oocytes, lacking expression of both proteins, severely impaired developmental progression of *Kdm2a*^{matKO}*Kdm2b*^{matKO} embryos toward the blastocyst stage, even though ovulation rates were normal (Figures 1B, 1C, and S1D). Such embryonic impairment was phenocopied by embryos maternally compound mutant for *Kdm2a*^{KO} and *Kdm2b*$^{\Delta CxxC}$ (Figures 1B and S1D). Therefore, we conclude that KDM2A and KDM2B serve, additively or redundantly, essential functions during oogenesis to support pre-implantation development.

KDM2A/KDM2B regulate PRC1-mediated gene repression

To assess the role of KDM2A/KDM2B in vPRC1.1- and other Polycomb-mediated functions, we first quantified the levels of PRC1-catalyzed H2AK119u1 in *ctrl* and mutant FGOs by immunofluorescence (IF) analyses. Whereas H2AK119u1 levels were not altered in FGOs singly deficient for either *Kdm2a* or *Kdm2b*$^{\Delta CxxC}$, they were massively reduced in *Kdm2a*^{KO}*Kdm2b*^{KO} and *Kdm2a*^{KO}*Kdm2b*$^{\Delta CxxC}$ FGOs (Figure 1D). These results argue that either KDM2A or KDM2B is sufficient for recruiting vPRC1 to chromatin, likely via their CxxC domains, to enable H2AK119u1 deposition. Moreover, PRC2-catalyzed H3K27me3 levels were greatly reduced in both types of double-mutant FGOs (Figure 1E), indicating that vPRC1 functions upstream of PRC2 in GOs, as reported for ESCs.[31]

To understand transcriptional regulation by KDM2A and KDM2B, we profiled transcriptomes of >20 individual FGOs from single and double-mutant and *ctrl* genotypes (Figure S2A).

(B) Developmental progression rates of *ctrl*, single, and double/compound mutant pre-implantation embryos at indicated days of *in vitro* embryonic development. *p* values according to Fisher's exact test.
(C–E) Immunofluorescence staining and quantification of KDM2A, cytosolic KDM2B, H2AK119u1, and H3K27me3 in GOs (KDM2A) or FGOs (others) of indicated genotypes. *p* values according to two-sided Student's t test. Scale bars, 20 μm.
For panels (B)–(E), numbers of embryos or oocytes analyzed are indicated.

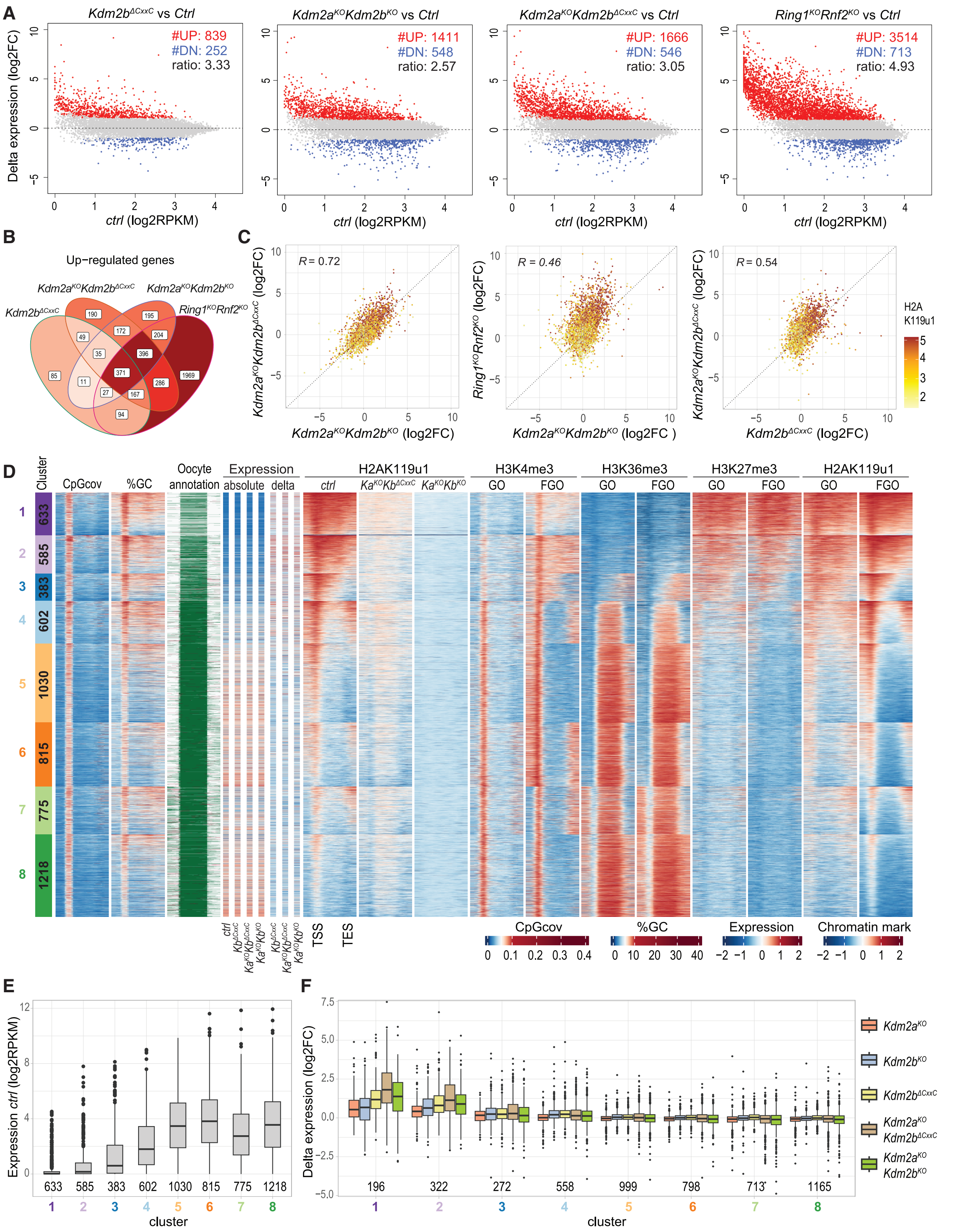

(legend on next page)

Compared with *ctrl* oocytes, 423, 522, and 839 genes were upregulated in *Kdm2a*^{KO}, *Kdm2b*^{KO}, and *Kdm2b*$^{\Delta CxxC}$ FGOs, respectively, while only 145, 192, and 252 genes were downregulated (Figures 2A and S2C). FGOs of the two *Kdm2b* mutation types showed a larger overlap in commonly upregulated genes than FGOs with either *Kdm2a* or *Kdm2b* mutation. Hence, KDM2A and KDM2B mainly function as transcriptional repressors with specific and common gene targets (Figures S2D and S2E). In *Kdm2a*^{KO}*Kdm2b*^{KO} and *Kdm2a*^{KO}*Kdm2b*$^{\Delta CxxC}$ FGOs, over 1,400 genes were upregulated while ~550 genes were downregulated, arguing for cooperative repressing roles between KDM2A and KDM2B (Figures 2A and S2D).

Most promoters of 974 genes commonly upregulated in *Kdm2a*^{KO}*Kdm2b*^{KO} and *Kdm2a*^{KO}*Kdm2b*$^{\Delta CxxC}$ FGOs are H2AK119u1-marked in *ctrl* oocytes[37] (Figure 2C; Tables S1 and S2). Genes upregulated in single mutant FGOs were also enriched for H2AK119u1 (Figure S2E). Further, 71% of genes upregulated in *Kdm2a*^{KO}*Kdm2b*^{KO} FGOs were also upregulated in FGOs deficient for *Ring1* and *Rnf2*, two core components of all PRC1 complexes, acting redundantly during oogenesis[36] (Figures 2A–2C, S2B, and S2F; Tables S1 and S2). Hence, these data characterize KDM2A/KDM2B as prominent PRC1-associated transcriptional repressors, in line with results of Gene Ontology analyses (Figure S2G).

The comparable misexpression in *Kdm2a*^{KO}*Kdm2b*^{KO} and *Kdm2a*^{KO}*Kdm2b*$^{\Delta CxxC}$ FGOs suggests that binding of KDM2B to CGI promoters via its CxxC domain is key for vPRC1-driven gene repression (Figure 2C). In line, of 1,666 genes upregulated in *Kdm2a*^{KO}*Kdm2b*$^{\Delta CxxC}$ FGOs, 37% were upregulated in single *Kdm2b*$^{\Delta CxxC}$ FGOs (Figure 2B). Another 41% were upregulated in *Ring*^{KO}*Rnf2*^{KO} FGO (Figure 2B; Tables S1 and S2). Together, contrasting results in ESCs,[29] our data support the notion that KDM2A functions as a vPRC1 member in oocytes, like KDM2B.

KDM2A/KDM2B recruit repressive PRC1 to chromatin

In FGOs, H2AK119u1 and H3K27me3 were previously reported to co-occupy broad genomic regions, while dual marking by H2AK119u1 and H3K4me3 was shown to label promoters of expressed genes.[21,37,39,40] To derive the syntax of DNA sequence and chromatin configurations underlying the gene regulatory function of KDM2A/KDM2B proteins, we selected CGI- and non-CGI-promoter genes (Figure S3A) and partitioned each gene group into 8 clusters by *k*-means clustering, based on occupancy levels of H3K4me3,[21] H3K27me3,[41] and H2AK119u1[37] at promoters and of H3K36me3[19] along gene bodies measured in WT FGOs (Figures 2D and S3B). For CGI-promoter genes, this resulted in clusters 1–3 harboring Polycomb-regulated genes while clusters 4–8 contain genes that had been transcribed and hence accumulated H3K36me3 during oocyte growth (Figures 2D and 2E). We then incorporated absolute and differential expression levels measured in *ctrl* and mutant FGOs (Figures 2D–2F and S3B–S3D). We further measured H2AK119u1 occupancies by CUT&RUN and observed an extensive to almost complete loss in all gene clusters in *Kdm2a*^{KO}*Kdm2b*$^{\Delta CxxC}$ and *Kdm2a*^{KO}*Kdm2b*^{KO} oocytes, respectively, both relative to *ctrl* FGOs (Figures 2D, S3B, and S3E). Primarily, the expression of CGI-promoter genes belonging to clusters 1, 2, and some in 3, characterized by extensive H2AK119u1 and H3K27me3, lack of H3K36me3, and low to no expression in WT GOs and FGOs, was upregulated in *Kdm2a*^{KO} and *Kdm2b*^{KO} FGOs and more pronounced in *Kdm2b*$^{\Delta CxxC}$, *Kdm2a*^{KO}*Kdm2b*$^{\Delta CxxC}$, and *Kdm2a*^{KO}*Kdm2b*^{KO} FGOs. By contrast, cluster 4–8 expressed genes with moderate-to-low H2AK119u1 yet high H3K4me3 levels at their promoters barely responded to KDM2A/KDM2B mutations (Figures 2D and 2F). By large, we observed a similar transcriptional response and chromatin logic for non-CGI-promoter genes, for which H2AK119u1 and H3K27me3 co-occupancy in WT oocytes relate to CpG/GC density (Figures S3B and S3D). Hence, while the KDM2A/KDM2B proteins control H2AK119u1 deposition at gene promoters throughout the genome, they only repress genes extensively co-labeled by H2AK119u1 and PRC2-mediated H3K27me3.

KDM2A/KDM2B restrict H3K36me2 and DNAme deposition in genes

Besides their role in vPRC1 recruitment, KDM2A/KDM2B can demethylate H3K36me1/2.[28] To test their catalytic function during oogenesis, we performed IF staining and measured an increase in H3K36me2 in *Kdm2a*^{KO}*Kdm2b*$^{\Delta CxxC}$ GOs. The increase was more significant in *Kdm2a*^{KO}*Kdm2b*^{KO} GOs lacking KDM2A/KDM2B demethylase activity (Figures 1A and 3A). By contrast, H3K36me3 was only slightly increased in both mutant GOs, which may relate to the weakly enhanced gene expression measured in mutant FGOs (Figures 2F, 3A, and S3D).[19]

Given the role of *Kdm2b* in preventing DNAme at Polycomb-regulated CGIs in ESCs,[25] we next assessed DNAme by IF, revealing a vast increase in 5mC levels in both double mutants (Figure 3A). We next performed whole-genome bisulfite sequencing (WGBS) and measured gains in global CpG

Figure 2. KDM2A/KDM2B regulate H2AK119u1 deposition and gene expression during oogenesis

(A) MA plots showing differential expression of indicated mutant FGOs over respective *ctrl* FGOs (log2 fold change [log2FC]) as a function of expression in respective *ctrl* FGOs (log2RPKM). #UP and #DN refer to numbers of more highly or lowly expressed genes in mutant versus *ctrl* FGOs (log2FC > 1.0; adj p value < 0.05). Ratio refers to #UP genes over #DN genes.

(B) Venn diagram showing numbers of genes upregulated in indicated mutant FGOs relative to respective *ctrl* FGOs.

(C) Scatterplots showing expression log2FC of indicated mutant FGOs over *ctrl* FGOs versus indicated mutant FGOs over *ctrl* FGOs. H2AK119u1 occupancy (log2) at promoters (−1,500/+500 bps of TSS) is indicated by color scale.[37] R indicates Pearson's correlation coefficient.

(D) Heatmap displaying sequence composition, transcriptional, and chromatin variables within CGI-promoter genes (5 kb upstream, TSS, gene body, TES, and 5 kb downstream) grouped into 8 clusters by *k*-means clustering. From left to right: gene numbers per cluster; CpG coverage; GC percentage; oocyte-specific sense (green) and antisense (red) transcripts[38]; absolute RNA (scaled RPKM) in *ctrl* and mutant FGOs; log2FC expression in mutant versus *ctrl* FGOs (delta); H2AK119u1 occupancy in *ctrl* and mutant FGOs; and H3K4me3, H3K36me3, H3K27me3, and H2AK119u1 occupancies in WT GOs and FGOs.[19,21,37] All chromatin data are shown as *Z* scores.

(E) Boxplot presenting RNA expression levels per gene cluster in *ctrl* FGOs with gene numbers per cluster indicated.

(F) Boxplot presenting log2FC in expression per gene cluster measured in various mutant FGOs relative to respective *ctrl* FGOs.

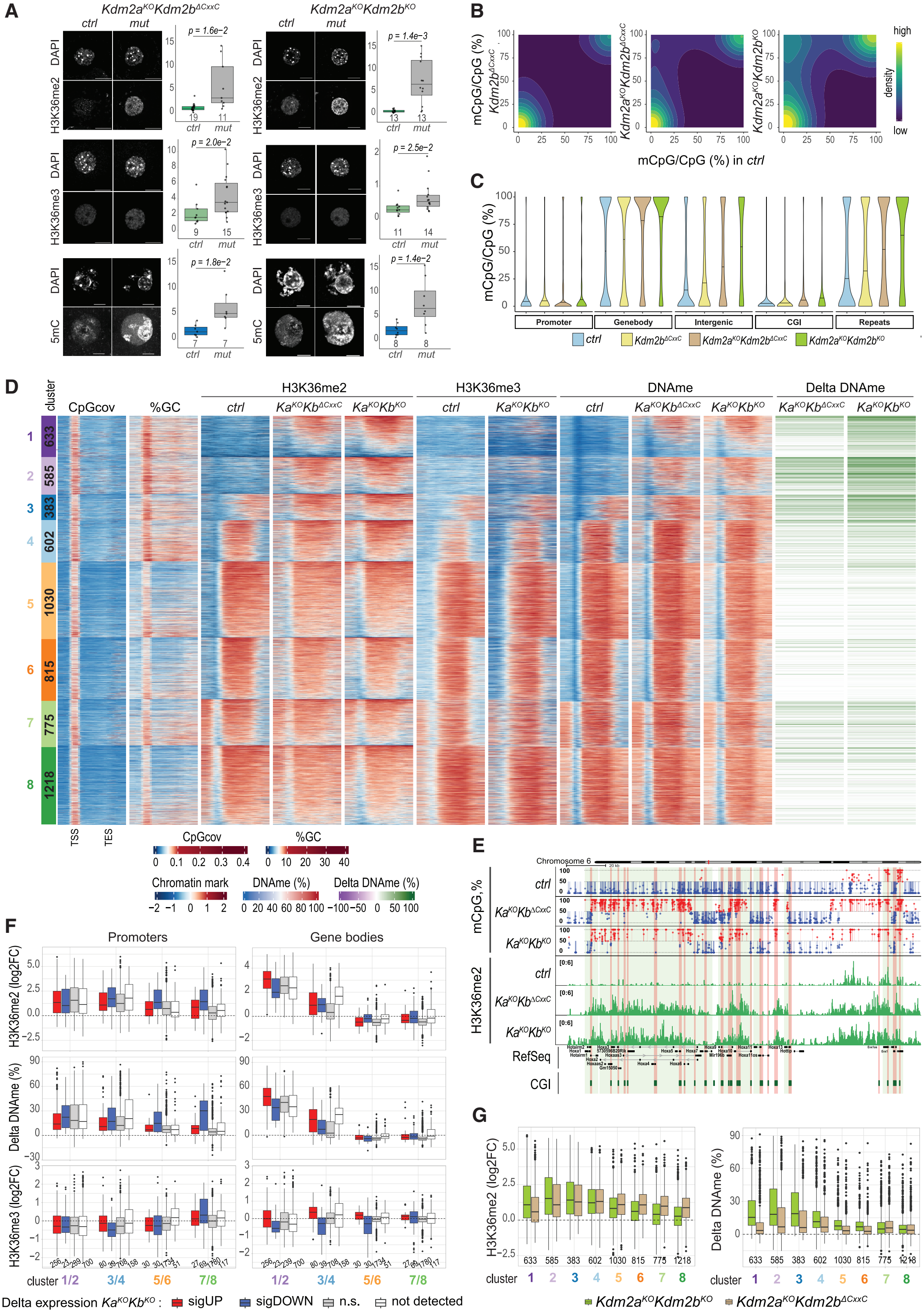

Figure 3. KDM2A/KDM2B restrict H3K36me2 and DNAme at promoters and along genes in oocytes

(A) Immunofluorescence staining and quantification of H3K36me2 and H3K36me3 in GOs and 5mC in FGOs of indicated mutant and respective *ctrl* genotypes. Numbers of analyzed oocytes are indicated. *p* values according to the two-sided Student's t test. Scale bars, 20 μm.

(legend continued on next page)

methylation of 35.8% mCpGs/CpGs in controls to 40.7% in *Kdm2b*$^{\Delta CxxC}$, 51.5% in *Kdm2a*^{KO}*Kdm2b*$^{\Delta CxxC}$, and 58.8% in *Kdm2a*^{KO}*Kdm2b*^{KO} FGOs (Figures 3B and S4A–S4C). DNAme gains were not limited to Polycomb-controlled promoter regions, as in *Kdm2b*-deficient ESCs,[25] but extended widely into gene bodies, intergenic regions, and endogenous repetitive elements (ERVs) (Figures 3C and 3D, displaying gene clusters as shown in Figure 2D), as exemplified for the *Hoxa* gene cluster (Figure 3E). Importantly, numerous non-Polycomb-controlled promoter CGIs acquired aberrant DNAme (Figures 3D and 3F).

Differential gene expression analysis did not reveal changes in expression of *Nsd1-3* and *Setd2* H3K36 methyltransferases nor of *Dnmts* in mutant FGOs (Figure S4D). Global DNMT3A protein levels appeared unchanged in *Kdm2a*^{KO}*Kdm2b*^{KO} FGOs as well (Figure S4E). Hence, to understand mechanisms underlying the differential increases in DNAme, we aimed to compare genome-wide distributions of DNAme, H3K36me2, and H3K36me3. We therefore performed CUT&RUN analyses for H3K36me2 in *ctrl*, *Kdm2a*^{KO}*Kdm2b*$^{\Delta CxxC}$, and *Kdm2a*^{KO}*Kdm2b*^{KO} FGOs and for H3K36me3 in *ctrl* and *Kdm2a*^{KO}*Kdm2b*^{KO} FGOs (Figure S4F). In *ctrl* oocytes, we observed a strong co-occupancy of H3K36me2, H3K36me3, and DNAme within gene bodies of transcribed CGI-promoter genes (clusters 4–8), as reported previously for H3K36me3 and DNAme (Figure 3D).[19,20] Regions upstream (clusters 7 and 8) and downstream (clusters 5 and 8) of expressed genes showed similar marking, likely reflecting initiation of transcription from upstream-located ERVs[38] and transcriptional run-through beyond annotated transcriptional end sites, respectively (Figure 3D).

In *Kdm2a*^{KO}*Kdm2b*$^{\Delta CxxC}$ and *Kdm2a*^{KO}*Kdm2b*^{KO} FGOs, we measured major gains in H3K36me2 and DNAme along gene bodies of cluster 1–3 genes compared with *ctrl* oocytes, reaching high levels characteristic of robustly expressed genes as those in clusters 5–8 (Figure 3D). By contrast, H3K36me3 levels remained largely unchanged along gene bodies of cluster 1–3 genes, suggesting that the rather moderate increases in expression upon *Kdm2a*/*Kdm2b* deficiency (Figures 2D, 2F, and 3D) are insufficient for robust *Setd2*-dependent transcription-coupled H3K36me3 deposition.[19] Hence, it is unlikely that aberrant DNAme acquisition at cluster 1–3 genes in mutant FGOs was instructed by H3K36me3 (Figure 3D). Quantitative enrichment analysis confirmed strong gains of both H3K36me2 and DNAme, but not of H3K36me3, in gene bodies of CGI- and non-CGI-promoter genes (Figures 3F and S4G). Importantly, H3K36me2 and DNAme were even increased at genes with unchanged or downregulated expression, or with non-detectable expression in mutant FGOs (Figure 3F). Further, gains in H3K36me2 and DNAme were more pronounced along cluster 1–3 genes of *Kdm2a*^{KO}*Kdm2b*^{KO} compared with *Kdm2a*^{KO}*Kdm2b*$^{\Delta CxxC}$ oocytes and even occurred at regions upstream and/or downstream of genes (Figures 3D, 3G, and S4G). Together, these data argue that the gain in H3K36me2 along gene bodies is not linked to transcription but results from the loss of H3K36me2 demethylase activity by KDM2A/KDM2B.

KDM2A/KDM2B control H3K36me2 and DNAme at CGI promoters

In addition to gene bodies, we measured increased H3K36me2 and DNAme at promoters of CGI and non-CGI promoters of genes marked by H2AK119u1/H3K27me3 in WT oocytes (Figures 2D, 3D, 3F, S3B, and S4G). Remarkably, even at cluster 4–8 CGI-promoter genes, which are not or only weakly marked by H2AK119u1, lack H3K27me3 at their promoters, and are expressed in *ctrl* oocytes, we measured significant gains in H3K36me2 and DNAme in both double-mutant FGOs, particularly for genes displaying decreased expression (Figures 2D, 3D, 3F, and 3G). These results differ from the reported gain in DNAme in *Kdm2b*-deficient ESCs, occurring exclusively at CGI promoters controlled by PRC1.[25] Instead, consistent with KDM2A and KDM2B localizing to all CGI promoters in ESCs irrespective of their chromatin status,[29,31] our data point to a widespread catalytic H3K36me2 demethylating function of KDM2A and KDM2B *in vivo*, protecting PRC1- and non-PRC1-controlled CGI promoters from gaining H3K36me2 and DNAme.

KDM2A/KDM2B prevent deposition of H3K36me2 and DNAme genome wide

To investigate whether non-genic regions respond similarly, we partitioned 10 kb-sized intergenic sequences into 8 clusters based on H3K4me3,[21] H3K36me3,[19] H3K27me3,[41] and H2AK119u1[37] occupancy in WT FGOs (Figure S5A) and then incorporated changes in H3K36me2, H3K36me3, and DNAme levels in mutant versus *ctrl* FGOs (Figure 4A). As for genes, GC-dense regions in clusters 1–3, broadly marked by H2AK119u1 and H3K27me3, were devoid of H3K36me2, H3K36me3, and DNAme in *ctrl* FGOs (Figures 4A and S5A). These regions gained H3K36me2 and DNAme moderately to majorly in *Kdm2a*^{KO}*Kdm2b*$^{\Delta CxxC}$ and *Kdm2a*^{KO}*Kdm2b*^{KO} FGOs, respectively (Figures 4A and S5B). Even GC-poor regions in clusters 5 and 6, marked by some H2AK119u1 and H3K27me3 in *ctrl* GOs, gained H3K36me2 and DNAme, particularly again in *Kdm2a*^{KO}*Kdm2b*^{KO} FGOs (Figures 4A and S5B). Hence, these data point to a prominent genome-wide role, beyond CGIs, for KDM2A/KDM2B proteins in maintaining H3K36me2 levels low during oocyte development (Figures 4B–4D).

(B) 2D-density plots displaying distributions of CpGs according to their mean methylation levels (mCpG/CpG in %) in mutant FGOs versus *ctrl* FGOs.

(C) Violin plot showing distribution of mCpG/CpG (%) values at different genome elements in FGOs of indicated genotypes.

(D) Heatmap displaying sequence composition and chromatin variables within 8 CGI-promoter gene clusters previously described in Figure 2D. From left to right: CpG coverage; GC percentage; H3K36me2, H3K36me3, and DNAme in FGOs of indicated genotypes; differential DNAme at CGI promoters in mutant versus *ctrl* FGOs.

(E) Genomics snapshot of the *Hoxa–Evx1* gene cluster illustrating gain in DNAme and H3K36me2 at CGI promoters (in orange) and gene bodies in mutant versus *ctrl* FGOs.

(F) Boxplots displaying differential H3K36me2, DNAme, and H3K36me3 at CGI promoters (−1,500/+500 bps of TSS) or gene bodies (+500 bps of TSS to TES) of genes assigned to gene clusters in which expression is either upregulated, downregulated, not changed, or not detected in *Kdm2a*^{KO}*Kdm2b*^{KO} FGOs relative to *ctrl* FGOs. Numbers of genes per condition are indicated.

(G) Boxplots displaying differential H3K36me2 and DNAme at promoters of all genes belonging to the 8 CGI-promoter gene clusters in *Kdm2a*^{KO}*Kdm2b*^{KO} and *Kdm2a*^{KO}*Kdm2b*$^{\Delta CxxC}$ FGOs relative to *ctrl* FGOs, as indicated. Numbers of genes per cluster are indicated.

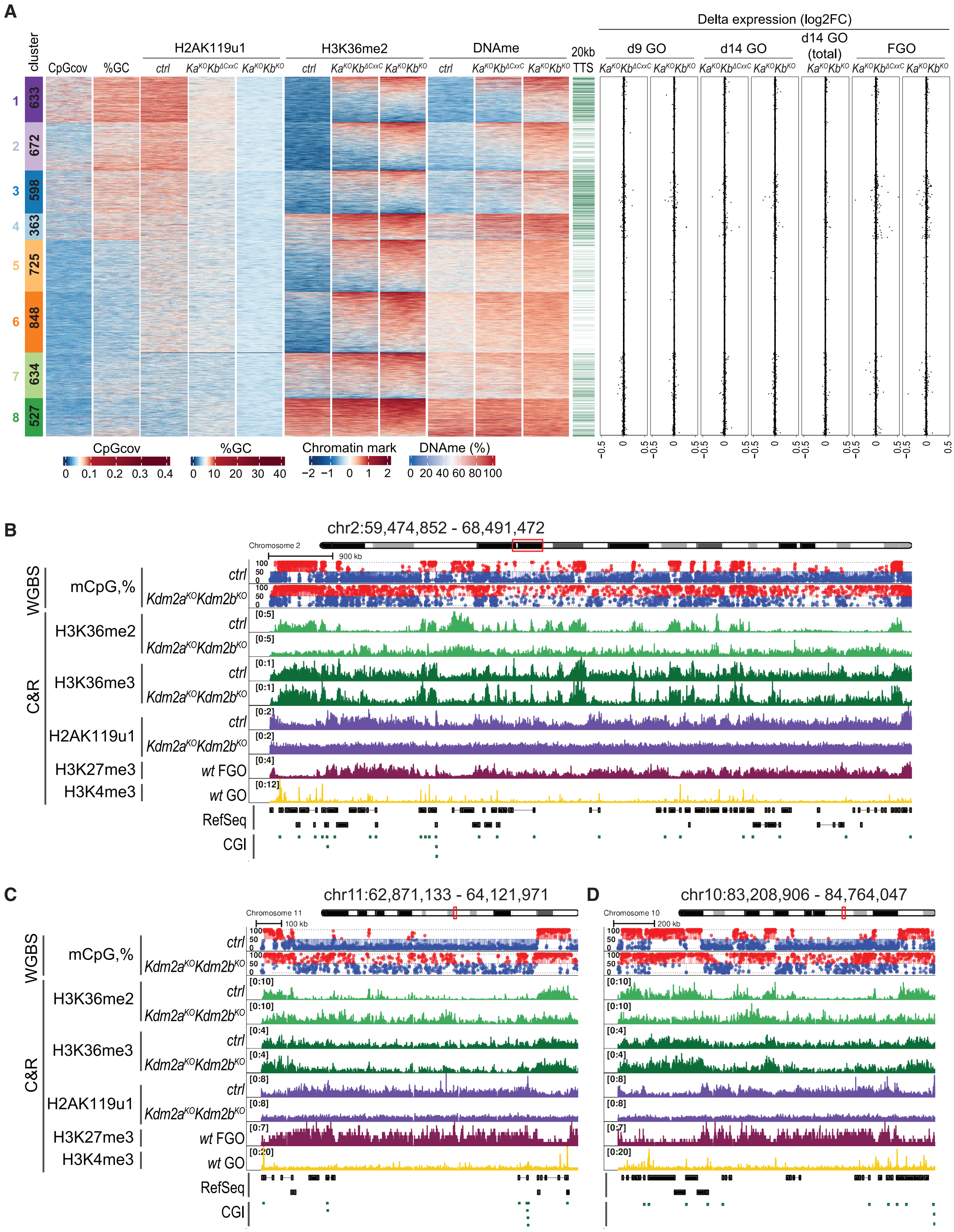

Figure 4. H3K36me2 and DNAme accumulate in *Kdm2a/Kdm2b*-deficient oocytes, independently of transcription

(A) Heatmap displaying chromatin and transcriptional variables within 8 clusters of 10 kb intergenic regions in oocytes. Data are displayed in 20 neighboring 500 bp bins. From left to right: number of regions per cluster; CpG coverage; GC percentage; H2AK119u1, H3K36me2, and DNAme in *ctrl* and mutant FGOs;

(legend continued on next page)

We did not measure upregulation of expression within 10 kb nor flanking regions having gained aberrant H3K36me2 in mutant FGOs (Figure 4A). To investigate possible expression changes during oocyte growth, we profiled GOs at days 9 and 14 of development using poly(A) and random primed RNA sequencing approaches (Figures S5C and S5D). Again, we did not measure consistent transcript level upregulation in regions gaining aberrant H3K36me2 (Figures 4A, S5E, and S5F). In summary, our data support a widespread transcription-independent deposition of H3K36me2 during oocyte growth that is efficiently counteracted by KDM2A/KDM2B proteins.

H3K4me3 in GOs prevents atypical DNAme acquisition

To identify chromatin features underlying aberrant *Dnmt3a*-mediated DNA methylation at CGIs, we performed regularized linear regression analysis for predicting DNAme, assuming additive effects of different sequence and chromatin parameters in GOs and FGOs. First, 82% of variation in CGI DNAme in *ctrl* FGOs could be explained, demonstrating the suitability of the approach. In keeping with previous reports,[11,19,20] H3K36me2 and H3K36me3 contributed positively, while H3K4me3 in WT FGOs represented the key negative predictor to CGI DNAme (Figures 5A and S6A–S6C).[21]

When employing chromatin features of *ctrl* oocytes, only 33% of differential DNAme between *Kdm2a*^{KO}*Kdm2b*^{KO} and *ctrl* FGOs could be explained. When integrating H3K36me2, H3K36me3, and H2AK119u1 occupancies in *Kdm2a*^{KO}*Kdm2b*^{KO} FGOs into the regression analysis, 63% of DNAme changes at CGIs could be accounted for, with H3K36me2 contributing positively and residual H2AK119u1 in *Kdm2a*^{KO}*Kdm2b*^{KO} FGOs negatively. Remarkably, H3K4me3 occupancy as measured in WT GOs but not FGOs was negatively correlated (Figures S6D–S6F). This suggests that CGIs are permissive for aberrant *de novo* DNAme only when H3K4me3 occupancy levels are low early during oocyte growth (Figure 5B).[20] In summary, our data are consistent with high H3K36me2 and low H3K4me3 occupancy serving instructive and permissive functions, respectively, for atypical DNAme acquisition at CGIs in mutant GOs.

Combinatorial sequence and chromatin features define aberrant H3K36me2 acquisition at CGIs

The globally increased H3K36me2 occupancy in *Kdm2a*^{KO} *Kdm2b*^{KO} FGOs points to widespread H3K36 methyltransferase activity in GOs. Nonetheless, H3K36me2 occupancy in *Kdm2a*^{KO}*Kdm2b*^{KO} FGOs was only increased at ~30% of CGIs that are normally controlled by PRC1. To resolve the regulatory complexity underlying specificity of atypical H3K36me2 at CGIs, we performed regularized linear regression analysis for H3K36me2 itself (Figures 5C, S6G, and S6H). This showed that aberrant H3K36me2 in *Kdm2a*^{KO}*Kdm2b*^{KO} FGOs is indeed deposited at CGIs normally marked by H2AK119u1 and to some extent by H3K27me3 in WT FGOs. In line, residual H2AK119u1 in *Kdm2a*^{KO}*Kdm2b*^{KO} FGOs associated negatively with atypical H3K36me2, a finding supported by biochemical studies demonstrating robust inhibition of all NSD and SETD2 lysine methyltransferases (KMTs) by nucleosomal H2AK119u1.[44,45] Moreover, as for DNA methylation, H3K4me3 in GOs is a negative predictor for atypical H3K36me2 occupancy, suggesting that H3K36 KMT function *in vivo* is inhibited by H3K4me3. This latter finding is in line with biochemical data for NSD3.[45]

We next aimed at understanding the principle underlying heterogeneity in H3K4me3 occupancy among CGIs in GOs and its possible negative impact on H3K36me2 and DNAme acquisition in *Kdm2a*^{KO}*Kdm2b*^{KO} GOs. At most CGI promoters in mouse oocytes, H3K4me3 is robustly catalyzed by the SETD1B KMT in conjunction with the CFP1 (CXXC1) cofactor, binding preferentially to CpGpG trinucleotides and reading out H3K4me3 as well.[41,46–48] At other promoters, intra- and intergenic sites, H3K4me3 is deposited by MLL2, which is recruited via its CxxC domain to CpG dinucleotides, having no preference for adjacent bases.[21,49] When including trinucleotide frequencies into our modeling, we identified a negative contribution of CpGpG trinucleotides to atypical H3K36me2 in *Kdm2a*^{KO} *Kdm2b*^{KO} FGOs (Figure 5C), suggesting that SETD1B/CFP1 catalyzing robust H3K4me3 may be the major barrier to NSD and/or SETD2-mediated catalysis at promoter CGIs in oocytes. Consistently, CGIs with low H3K4me3 in GOs are characterized by low CpGpG densities and harbor higher H3K36me2 in FGOs when H2AK119u1 levels are reduced (Figures 5D, S6I, and S6J). In summary, our analyses reveal that CGIs with a low frequency of CpGpG trinucleotides are particularly vulnerable toward aberrant accumulation of H3K36me2 and DNAme (Figure 5E).[45]

Aberrant maternal DNAme impairs pre-implantation development

To study the impact of increased DNAme in maternal genomes of *Kdm2a/Kdm2b* mutant oocytes for embryonic development, we investigated first its stability upon fertilization by IF analysis on zygotes. Impressively, contrasting the global loss of DNAme in the sperm genome, aberrant methylation in the maternal genome originating from *Kdm2a*^{KO}*Kdm2b*^{KO} oocytes did not become distinctively reprogrammed upon fertilization (Figure 6A), in keeping with parent-of-origin-specific epigenetic reprogramming of regular DNAme.[3] In line with less extensive gains of aberrant DNAme in *Kdm2a*^{KO}*Kdm2b*$^{\Delta CxxC}$ (than *Kdm2a*^{KO}*Kdm2b*^{KO}) FGOs, we did not observe a significant difference in global maternal DNAme levels in *Kdm2a*^{matKO}*Kdm2b*$^{mat\Delta CxxC}$ zygotes versus *ctrl* zygotes (Figure 6A). We next addressed whether the aberrant maternal methylome underlies the low developmental competence of both types of *Kdm2a/Kdm2b* mutant oocytes. To do so, we prevented the establishment of DNAme in *Kdm2a*^{KO}*Kdm2b*^{KO} and *Kdm2a*^{KO}*Kdm2b*$^{\Delta CxxC}$ GOs by conditionally mutating *Dnmt3a* function.[12] Significantly, embryos maternally triple-deficient developed into blastocyst-stage embryos equally efficiently as control embryos. Thus, maternal

presence of annotated TTS in 20 kb flanking regions that could be compatible with run-through transcription through the window; log2FC in expression between indicated mutant and *ctrl* genotypes in day 9 and day 14 GOs; in random primed (total) day 14 GOs and in FGOs. RNA expression data are based on poly(A)-primed RNA capture and Smart-seq2 library generation, if not indicated otherwise.
(B–D) Genomic interval[15,42,43] snapshots illustrating gain of DNAme and H3K36me2 at large intergenic regions in *Kdm2a*^{KO}*Kdm2b*^{KO} FGOs that are marked by H2AK119u1 and H3K27me3 in *ctrl* FGOs.

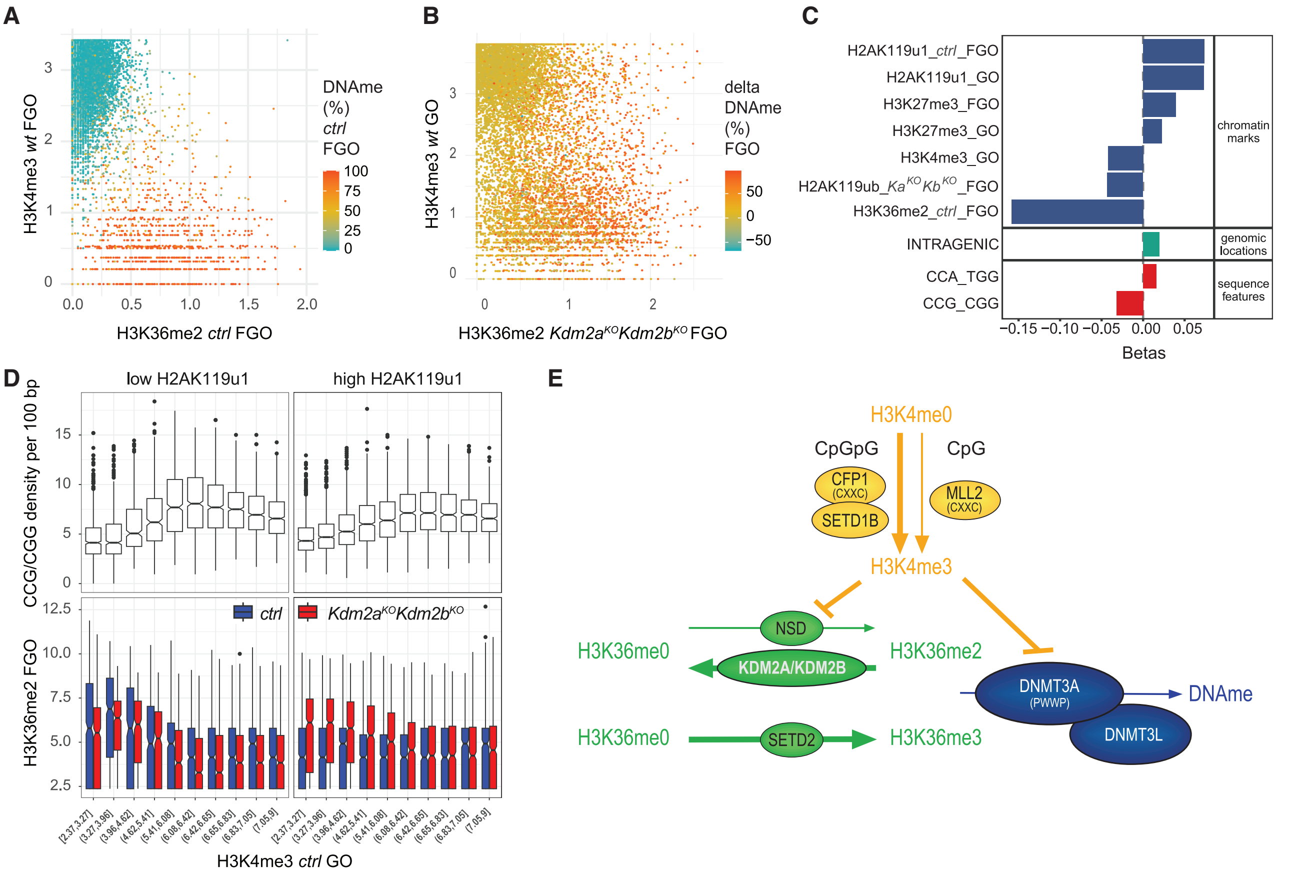

Figure 5. Low H3K4me3 in GOs is permissive for H3K36me2 and DNAme acquisition during oocyte growth

(A) Scatterplot showing correlations between H3K36me2 and H3K4me3 occupancies and DNAme (%) at promoter and intragenic CGIs in *ctrl* and WT FGOs.

(B) Scatterplot showing differential DNAme (%) at promoter and intragenic CGIs between *Kdm2a*^{KO}*Kdm2b*^{KO} over *ctrl* FGOs in relation to H3K36me2 occupancy in *Kdm2a*^{KO}*Kdm2b*^{KO} FGOs and H3K4me3 occupancy in WT GOs.

(C) Bar plot showing linear regression coefficients of chromatin marks, genomic location, and triplet nucleotide sequences contributing to predicting differential H3K36me2 occupancy at CGIs in *Kdm2a*^{KO}*Kdm2b*^{KO} over *ctrl* FGOs (R^2 = 0.338).

(D) Boxplots representing frequencies of CCG/CGG trinucleotides per 100 bp and enrichments of H3K36me2 in *ctrl* and *Kdm2a*^{KO}*Kdm2b*^{KO} FGOs at 533 bp regions surrounding CGI centers for bins of H3K4me3 occupancies in GOs having low or high H2AK119u1 levels.

(E) Cartoon illustrating dependencies between DNA sequences, histone modifying enzymes, and DNA methyltransferases in regulating H3K36 di-/tri-methylation and *de novo* DNA methylation.

Dnmt3a deficiency elicited complete suppression of the progressive early embryonic lethality seen for both maternal *Kdm2a/Kdm2b* mutants (compare Figures 1B to 6B). By contrast, deficiency of the DNMT1 enzyme, normally contributing together with UHRF1 to *de novo* DNAme at certain inactive regions in late GOs and maintaining DNAme during pre-implantation development,[50–52] did not rescue the embryonic lethality caused by *Kdm2a/Kdm2b* deficiency in oocytes (Figures 1B and 6B). In summary, these data indicate that beyond regulating vPRC1-mediated gene repression, one essential intergenerational function of KDM2A and KDM2B in oocytes is confining the targeting of DNMT3A and *de novo* DNAme catalysis by preventing H3K36me2 accumulation throughout the genome.

Maternal *Kdm2a/Kdm2b* deficiency impairs post-implantation development

To address possible other intergenerational functions of maternal *Kdm2a/Kdm2b* expression beyond controlling maternal DNAme, we studied post-implantation development of offspring of single, double, or triple maternally deficient females mated with WT C57BL/6J males. In line with our pre-implantation data (Figure 1B), we isolated only 1 *Kdm2a*^{matKO} *Kdm2b*$^{mat\Delta CxxC}$ embryo that was growth retarded at day 9.5 of embryonic development (e9.5) and no *Kdm2a*^{matKO}*Kdm2b*^{matKO} embryos (Figure 6C). By contrast, we recovered at e9.5 a comparable number of developmentally retarded embryos and implantation sites for *Dnmt3a*^{KO}*Kdm2a*^{KO}*Kdm2b*$^{\Delta CxxC}$ mothers as for *Dnmt3a*^{KO} mothers. Hence, maternal *Dnmt3a* deficiency rescues early post-implantation development of *Dnmt3a*^{matKO} *Kdm2a*^{matKO}*Kdm2b*$^{mat\Delta CxxC}$ embryos to an extent as observed for *Dnmt3a*^{matKO} single mutant embryos, which in turn suffer from defects related to DNMT3A's critical role in controlling genomic imprinting.[12,53] For *Dnmt3a*^{KO}*Kdm2a*^{KO}*Kdm2b*^{KO} triple mutant mothers, however, the overall number and percentage of living and developmentally retarded embryos at e9.5 were greatly reduced (Figure 6C). These data indicate that beyond

CellPress
OPEN ACCESS

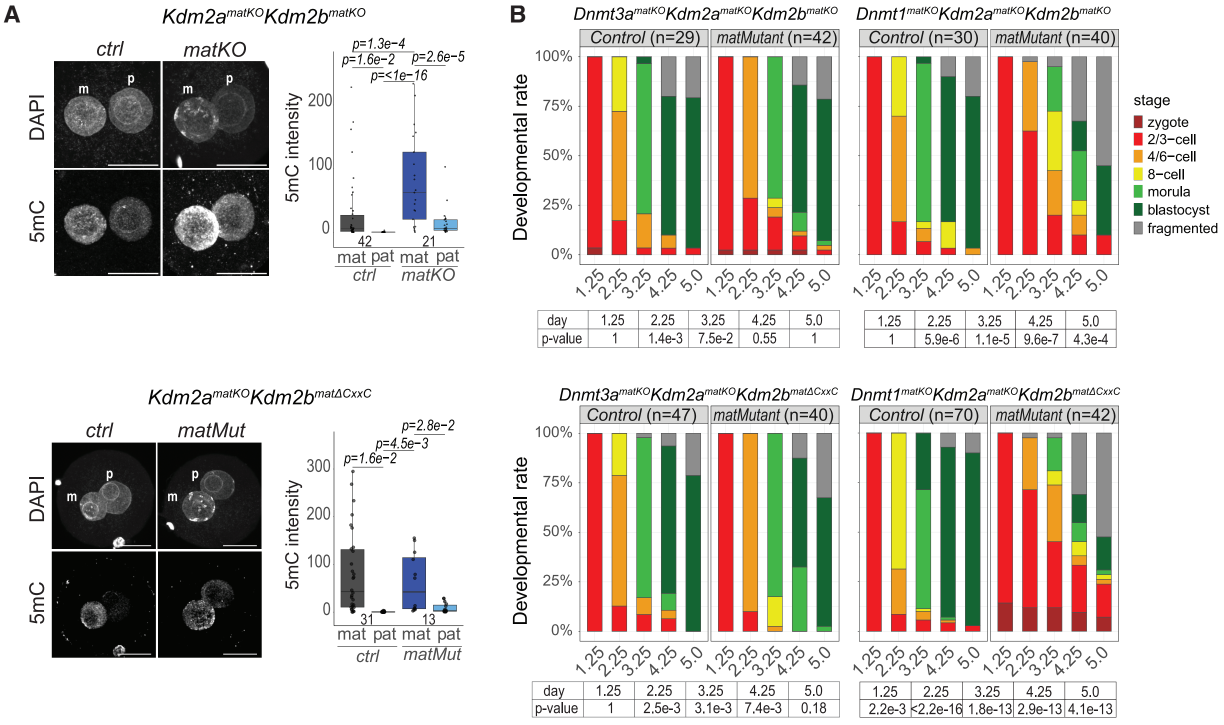

C

Postimplantation development

Stage	Mother	N of mothers	Father	N of implantation sites	N of embryos	N of normal embryos	N of developmental retardation cases	N of dead embryos	N of implantation sites w/o embryo
e9.5	*ctrl*	4	C57BL/6J	40	37	36	1	0	3
e9.5	$Kdm2a^{KO}Kdm2b^{KO}$	5	C57BL/6J	0	0	0	0	0	0
e9.5	$Kdm2a^{KO}Kdm2b^{\Delta CxxC}$	3	C57BL/6J	1	1	0	1	0	0
e9.5	$Dnmt3a^{KO}$	3	C57BL/6J	20	18	0	12	6	2
e9.5	$Dnmt3a^{KO}Kdm2a^{KO}Kdm2b^{KO}$	6	C57BL/6J	9	4	0	4	0	5
e9.5	$Dnmt3a^{KO}Kdm2a^{KO}Kdm2b^{\Delta CxxC}$	5	C57BL/6J	30	22	0	17	5	8

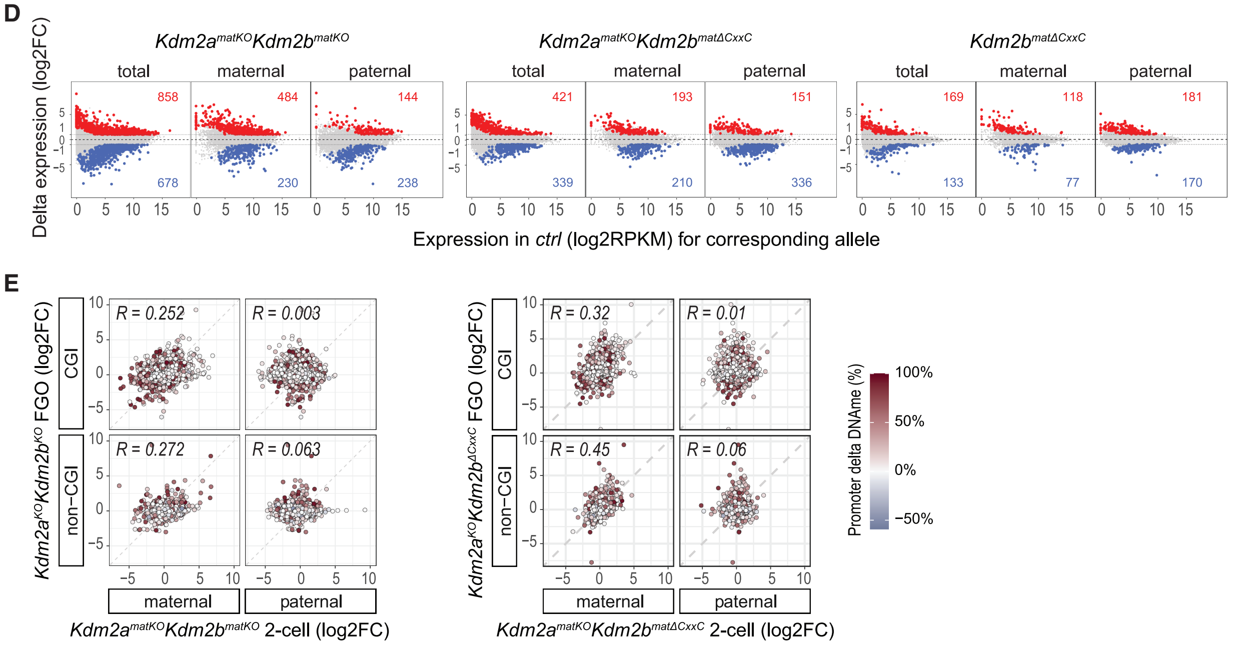

Figure 6. Aberrant maternal DNA methylation impairs pre-implantation development

(A) Immunofluorescence staining and quantification of 5mC in maternal (m) and paternal (p) pronuclei of *ctrl* and mutant late zygotes (numbers indicated). *p* values according to Tukey's HSD test. Scale bars, 20 μm.

(legend continued on next page)

controlling maternal DNAme acquisition at CGIs, as seen in both types of *Kdm2a/Kdm2b* mutant FGOs (Figure 3D), a second oocyte and/or maternal function of KDM2A/KDM2B, presumably acting throughout the remaining genome, as observed in $Kdm2a^{KO}Kdm2b^{KO}$ FGOs (Figures 3 and 4), is critical for post-implantation development. The underlying molecular mechanism warrants further investigations.

Aberrant DNAme impairs gene expression in oocytes

To assess the impact of maternal *Kdm2a/Kdm2b* deficiency on gene regulation in embryos, we profiled differential expression in $Kdm2a^{matKO}Kdm2b^{matKO}$ versus *ctrl* two-cell embryos sired by JF1/Ms males (Figures S1B and S7A–S7D). Hundreds of genes were misregulated, with clear allele-specific responses (Figure 6D; Tables S4 and S5). Differential expression in oocytes and two-cell embryos was positively correlated for maternal but not paternal alleles (Figure 6E). Importantly, 36% of 157 maternally expressed CGI-promoter genes that were downregulated in mutant two-cell embryos were hypermethylated (>50%) at their promoters in $Kdm2a^{KO}Kdm2b^{KO}$ oocytes (Figure 6E). Counterintuitively, multiple genes with methylated CGIs had upregulated gene expression in mutant FGOs (Figure 6E). We observed comparable transcriptional and methylation responses in $Kdm2a^{matKO}Kdm2b^{mat\Delta CxxC}$ and, to a lesser degree, in single mutant $Kdm2b^{mat\Delta CxxC}$ samples (Figures 6D and 6E).

To dissect in more detail the mechanistic relationship between aberrant DNAme and transcription in mutant oocytes and two-cell embryos, we first grouped CGIs located within UCSC-defined promoters in 7 clusters according to their DNAme status in *ctrl* and three types of mutant FGOs and in WT sperm (Figure 7A). In clusters 1 to 4, almost 1,500 CGI promoters had gained extensive aberrant DNAme in *Kdm2a/Kdm2b* mutant FGOs. Further, over 1,250 CGI promoters of cluster 5 genes gained aberrant DNA to moderate levels (<50%), particularly in $Kdm2a^{KO}Kdm2b^{KO}$ oocytes. Consistent with their identity as PRC1-target genes, Gene Ontology term analysis showed that many genes of clusters 1–5 serve important functions during development (Figure 7B; Table S6). By contrast, cluster 6 contained CGIs that are highly methylated in *ctrl* and mutant FGOs. These CGIs are likely localized within gene bodies transcribed from alternative upstream oocyte-specific promoters, despite their UCSC classification as promoters. In accord, these CGIs are characterized by low H3K4me3 and high H3K36me3 occupancy and by high transcript levels upstream of the CGI in WT FGOs (Figures S7E–S7G). Cluster 7 CGIs were either unmethylated or harbored only low DNAme levels in any genotype. Notably, none of the UCSC-annotated CGIs were substantially methylated in mature spermatozoa[54] (Figure 7A).

We next related aberrant CGI-promoter DNAme to gene expression changes. In FGOs, upregulated and downregulated genes were rather evenly distributed among the different clusters (Figures 7C and S7H). Nonetheless, aberrant DNAme was significantly associated with CGIs of cluster 2–4 genes that had been transcriptionally downregulated in double-mutant FGOs (Figures 7D and S7I). Thus, this likely reflects direct repression of CGI promoters by aberrant DNAme resulting from H3K36me2 accumulation (Figure S7G).

Counterintuitively, aberrant DNAme at other UCSC-annotated CGIs of clusters 2–5 was significantly associated with genes that were upregulated in both double-mutant FGOs but not mutant embryos (Figures 7D and S7I). As for cluster 6 CGIs, the DNAme gain at such UCSC-annotated promoter CGIs likely stems from a transcription-coupled process that relates to their localization within genomic regions that become aberrantly transcribed in GOs from oocyte-specific promoters located upstream of these UCSC-annotated CGIs and that are normally repressed by PRC1. Indeed, their aberrant transcripts were elevated, not only downstream but also upstream of such CGIs (Figure S7G).

Aberrant DNAme associates with maternal gene repression in two-cell embryos

We next evaluated whether aberrant DNAme at CGI promoters may inhibit expression of genes that normally become activated during zygotic genome activation (ZGA). We first identified genes that become upregulated or downregulated in two-cell embryos upon inhibition of RNA polymerase II and III by alpha-amanitin.[55] We observed that clusters of 3–5 genes with aberrantly methylated CGI promoters in *Kdm2a/Kdm2b* mutant FGOs were significantly enriched among genes downregulated but not upregulated in response to alpha-amanitin treatment (Figure 7E).

To test this notion, we studied parental allele-specific gene expression in two-cell embryos. Genes associated with clusters 2–4 CGI promoters that had robust aberrant DNAme in $Kdm2a^{KO}Kdm2b^{KO}$ and $Kdm2a^{KO}Kdm2b^{\Delta CxxC}$ oocytes were significantly downregulated from maternal but not paternal alleles (Figures 7C, 7D, S7H, and S7I). In contrast to FGOs, aberrant DNAme was not associated with upregulated maternal expression in mutant two-cell embryos (Figures 7D and S7I). Hence, these results argue that aberrant maternal DNAme inherited from oocytes represses gene transcription during pre-implantation development, including up to 500 ZGA-induced genes.

Aberrant maternal DNAme is maintained in four-cell embryos

To assess propagation of maternal DNAme during early pre-implantation development, we performed WGBS on *Ctrl* and

(B) Developmental progression rates of *ctrl* and triple mutant pre-implantation embryos at indicated days of *in vitro* embryonic development. *p* values according to Fisher's exact test.
(C) Table summarizing post-implantation development of embryos with indicated genotypes.
(D) MA plots showing differential expression in maternally mutant over respective *ctrl* two-cell embryos (log2FC) as a function of expression in *ctrl* two-cell embryos (log2RPKM) for all (total) and parental-specific sequencing reads. Genes upregulated or downregulated in mutants are indicated in red and blue (|log2FC| > 1.0; adj *p* value < 0.05).
(E) Left: scatterplot showing log2FC expression of $Kdm2a^{matKO}Kdm2b^{matKO}$ over *ctrl* two-cell embryos versus log2FC expression of $Kdm2a^{KO}Kdm2b^{KO}$ over *ctrl* FGOs for parental-specific expression of CGI- and non-CGI-promoter-associated genes. Right: data as in the left panel for $Kdm2a^{matKO}Kdm2b^{mat\Delta CxxC}$ over *ctrl* two-cell embryos versus $Kdm2a^{KO}Kdm2b^{\Delta CxxC}$ over *ctrl* FGOs. Differential promoter methylation (%) in $Kdm2a^{KO}Kdm2b^{KO}$ over *ctrl* FGOs is indicated by color.

CellPress
OPEN ACCESS

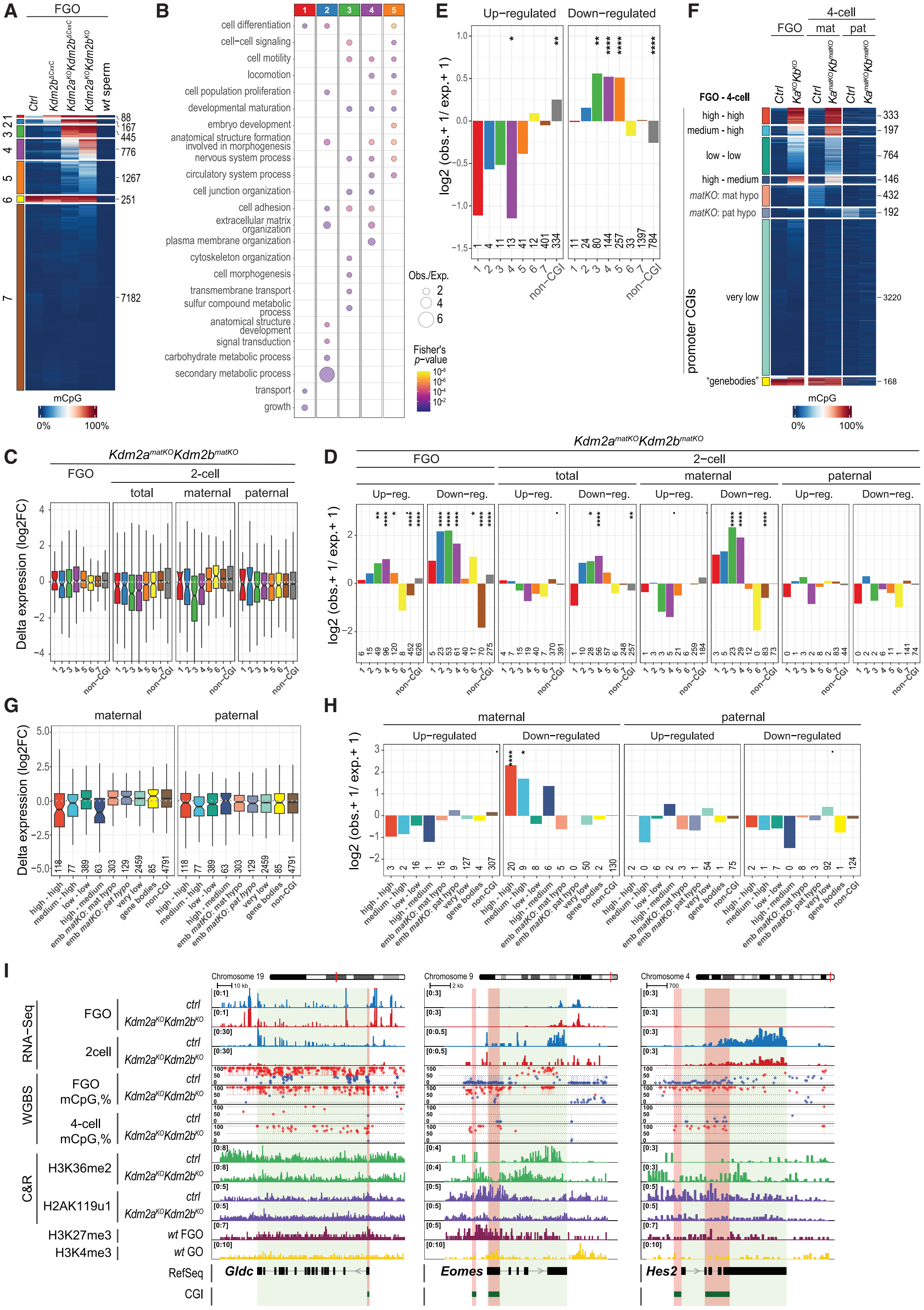

Figure 7. Genes marked by aberrant maternal promoter DNAme are repressed in early embryos

(A) Heatmap showing clustering of UCSC-based CGI promoters (numbers indicated) according to absolute promoter methylation levels in *ctrl* and mutant FGOs and in WT sperm.[54]

(legend continued on next page)

$Kdm2a^{matKO}Kdm2b^{matKO}$ four-cell embryos sired by JF1 fathers, allowing discrimination of DNAme at 5,452 maternal and paternal CGI promoters (Figures 7F and S7J; Table S3). Remarkably, 333 of 479 CGIs with high-level aberrant DNAme (≥75%) in $Kdm2a^{KO}Kdm2b^{KO}$ FGOs retained aberrantly high-level DNAme in four-cell embryos, while 146 displayed a 2-fold reduction to medium (~50%) DNAme levels at maternal alleles in four-cell embryos. Even low levels of DNAme (~10% to 30%) at 764 CGIs remained stably maintained. Principally, passive, replication-related loss of DNAme would have resulted in a 4- to 8-fold reduction in DNAme pending on the cell cycle stage of the four-cell embryos. Hence, our data point to proficient propagation of aberrant maternal DNAme from FGOs to 4-cell stage embryos (Figure S7J). Such efficient propagation resembles proficient DNAme maintenance observed at 168 CGIs that had acquired high DNAme levels in *ctrl* and mutant oocytes, presumably in a transcription-coupled process (cluster 6 in Figures 7A and S7E–S7G, yellow cluster in 7F). Such similarity may point to analogous mechanisms, a notion to be further studied.

Moreover, 197 CGIs with medium-level aberrant DNAme in $Kdm2a^{KO}Kdm2b^{KO}$ FGOs displayed even high-level DNAme in four-cell embryos, pointing to a *de novo* gain of DNAme at such CGIs during embryogenesis (Figure 7F). Finally, we observed reduced low-level *de novo* DNA methylation at maternal and paternal CGI alleles in $Kdm2a^{matKO}Kdm2b^{matKO}$ compared with *ctrl* embryos (Figures 7F and S7J). It remains to be determined whether such *de novo* DNAme in *ctrl* embryos depends on maternal *Dnmt3a* function, as reported previously for certain CGIs on the paternal genome,[56] and whether genomic regions compete for available maternal DNMT3A protein in early embryos.

Hypermethylation affects promoters of key developmental regulatory genes

Relating allelic CGI-promoter DNAme in four-cell embryos with allelic gene expression in two-cell embryos confirmed the repressive function of aberrant DNAme during early embryonic development (Figures 7F–7H). For example, zygotic expression of the *glycine decarboxylase* gene (*Gldc*), encoding a key enzyme in glycine metabolism,[57–59] is majorly suppressed in maternally deficient *Kdm2a/Kdm2b* two-cell embryos (Figure 7I). Other factors regulating blastocyst development (*Eomes*, *Gata4*, *Hnf1b*, *Junb*, *Klf4*, and *Sox17*) (Figure 7I), placenta development (*Atrx*, *Hand1*, *Lhx3*, *Vash2*, and *Wnt2*), and gastrulation (*Bmp4*, *Brachyury* [*T*], *Sox7*, and *Tlx2*) are also hypermethylated at their promoters. Indeed, Gene Ontology analysis revealed that many genes with hypermethylated CGI promoters serve major functions throughout post-implantation embryonic development, e.g., in cell fate specification and determination, morphogenesis, cellular differentiation, and cell cycle regulation, e.g., like *Hes2* (Figures 7B and 7I; Table S6). They include members of various transcription factor gene families, such as *Foxo*, *Gata*, *Hand*, *Hes*, *Hox*, *Lhx*, *Nkx*, *Pax*, *Six*, *Sox*, *Tead*, *Tbx*, and *Zfp*. Gene families involved in cell signaling, growth factor activity, and cell adhesion were also affected (*Bmp*, *Dll*, *Fgf*, *Igf2*, *Pdgf*, *Pcdh*, *Rar*, *Wnt*, and *Tgfb*), as were genes functioning in gonadal development and synaptonemal complex assembly. Hence, KDM2A and KDM2B protect large sets of CGI promoters of key developmental regulators against hypermethylation in oocytes, thereby safeguarding development.

DISCUSSION

In this study, we identify the mechanism specifying the hypo-DNA-methylated genome characteristic of mouse oocytes. We further demonstrate the necessity of hypomethylation of the oocyte genome for embryonic development and correct gene expression following fertilization. Hypermethylation impairs zygotic gene expression and embryonic viability.

Our research reveals that in oocytes DNMT3A-catalyzed *de novo* DNA methylation acquisition beyond transcriptional units is principally instructed by H3K36me2 (Figure 3). KDM2A and KDM2B serve critical roles in GOs in limiting H3K36me2 occupancy within and between genes as well as at gene promoters. Our data support that KDM2A/KDM2B demethylate H3K36me2 via their enzymatic JmjC domain.[27,28] In addition, by recruiting vPRC1.1 complexes that catalyze abundant and widespread H2AK119u1 on chromatin, KDM2A/KDM2B may also inhibit NSD and/or SETD2 histone methyltransferases from depositing H3K36me2/me3.[45]

At CGIs, we resolved the syntax of a multi-layered sequence and chromatin modifier interaction network specifying unmethylated versus methylated DNA states. In line with biochemical

(B) Level of enrichments and statistical significances in Gene Ontology enrichment analyses for genes associated with CGIs belonging to DNAme clusters 1–5 shown in (A).

(C) Boxplot showing log2FC expression of clusters of CGI-promoter and all non-CGI-promoter genes in $Kdm2a^{KO}Kdm2b^{KO}$ over *ctrl* FGOs and in $Kdm2a^{matKO}Kdm2b^{matKO}$ over *ctrl* two-cell embryos according to all and parental-specific sequencing reads.

(D) Bar plot showing over-/under-representation and statistical significance of CGI-promoter genes in DNAme clusters and being either significantly up- or down-expressed in $Kdm2a^{KO}Kdm2b^{KO}$ relative to *ctrl* FGOs or in $Kdm2a^{matKO}Kdm2b^{matKO}$ relative to *ctrl* two-cell embryos for all and parental-specific sequencing reads. Statistical significance (*p* value) is coded as follows: ****$p \leq 0.001\%$, **$p \leq 0.1\%$, *$p \leq 1\%$, $p \leq 5\%$.

(E) Bar plot showing over-/under-representation and statistical significance of CGI-promoter genes in DNAme clusters that are significantly upregulated or downregulated in two-cell embryos treated with alpha-amanitin, an inhibitor of RNA polymerase II and III.[55] *p* value: ****$p \leq 0.001\%$, **$p \leq 0.1\%$, *$p \leq 1\%$.

(F) Heatmap showing clustering of UCSC-based CGI promoters according to absolute promoter DNAme levels in *ctrl* and $Kdm2a^{KO}Kdm2b^{KO}$ FGOs and *ctrl* and $Kdm2a^{matKO}Kdm2b^{matKO}$ four-cell embryos at maternal and paternal genomes. Mat- and pat-hypo refer to CGIs undergoing *de novo* methylation in *ctrl* embryos. "Genebodies" refers to CGIs having undergone transcription-coupled *de novo* DNAme in GOs.

(G) Boxplot showing log2FC expression of different clusters of CGI- and non-CGI-promoter genes shown in (F) in $Kdm2a^{matKO}Kdm2b^{matKO}$ over *ctrl* two-cell embryos according to parental-specific sequencing reads.

(H) Bar plot showing over-/under-representation and statistical significance of CGI-promoter genes in DNAme clusters as defined in (F) and being either significantly up- or down-expressed in $Kdm2a^{matKO}Kdm2b^{matKO}$ relative to *ctrl* two-cell embryos according to parental-specific sequencing reads. *p* value: ****$p \leq 0.001\%$, *$p \leq 1\%$, $p \leq 5\%$.

(I) Genomic snapshots of *Gldc*, *Eomes*, and *Hes2* genes with aberrant DNAme at CGI promoters (in orange) in oocytes and four-cell embryos. RNA expression in FGOs and two-cell embryos (both alleles), DNA methylation in FGOs and four-cell embryos (maternal alleles), and chromatin marks in FGOs are indicated.

assays,[23,45] our results show that KDM2A/KDM2B coordinate the balance between local H2AK119u1 and H3K36me2 levels, thereby controlling downstream *de novo* DNAme acquisition in GOs, with local low H3K4me3 occupancy being permissive (Figure 5). Our work further shows that a selective set of CGIs is particularly vulnerable to aberrant H3K36me2 and DNAme acquisition. These CGIs are characterized by low CpGpG trinucleotide frequencies, which likely limit recruitment of the CFP1/SETD1B complex driving high H3K4me3 levels.[41,45,46,48]

It remains to be determined whether KDM2A and/or KDM2B serve related functions in male germ cells, thereby possibly protecting CGIs also against erosion through deamination of methylated CpGs during evolution.[2] During gastrulation, *Kdm2b* and particularly *Mll2* have been shown to partially suppress DNAme acquisition in the epiblast of gastrulating embryos.[60] While functional redundancy by paralogs needs to be considered, these findings point to a universal mechanism keeping H3K36me2 occupancy levels at CGIs low, thereby preventing aberrant CGI DNAme during the mammalian life cycle. Recently, experimentally induced DNAme at CGI promoters of the *low-density lipoprotein receptor* (*Ldlr*) and *Ankyrin repeat domain 26* (*Ankrd26*) genes was associated with transgenerational inheritance of reduced gene expression and metabolic phenotypes across multiple generations.[61] Intriguingly, we observed 39% and 52% DNAme at the *Ldlr* CGI in $Kdm2a^{KO}Kdm2b^{KO}$ and $Kdm2a^{KO}Kdm2b^{\Delta CxxC}$ FGOs, respectively, compared with 0% in *ctrl* FGOs. *Ldlr* was also transcriptionally downregulated in mutant FGOs. For *Ankrd26*, we measured 30% aberrant DNAme in $Kdm2a^{KO}Kdm2b^{KO}$ FGOs (Tables S1, S2, and S3). For the *Ldlr* CGI, previous work argued that an acquired epigenetic state other than DNAme confers epigenetic inheritance through the germ line.[61] Our work suggests that the presence of H3K36me2 and absence of H3K4me3 may contribute to such memory.

Further, our study demonstrates the importance of the CxxC domain of KDM2B in recruiting vPRC1.1 to CGIs to deposit H2AK119u1. Interestingly, the absence of the CxxC domain did not prevent $KDM2B^{\Delta CxxC}$ from maintaining low H3K36me2 occupancy levels and precluding DNAme acquisition at genomic regions other than CGIs, as measured in $Kdm2a^{KO}Kdm2b^{\Delta CxxC}$ versus $Kdm2a^{KO}Kdm2b^{KO}$ oocytes. Such activities may reflect physiological functions of KDM2B beyond CGIs, e.g., in non-canonical imprinting that is critical for post-implantation development (Figure 6C).[37]

Moreover, absence of KDM2A/KDM2B proteins provoked widespread de-repression of PRC1/PRC2 target genes. Nonetheless, such transcriptional de-repression did not result in significant H3K36me3 deposition within gene bodies. As observed for the Set2 homolog in *Saccharomyces cerevisiae*,[62] SETD2 deposits H3K36me3 co-transcriptionally along with RNA polymerase 2, probably requiring multiple rounds of transcription to accumulate sufficient levels of the mark within gene bodies. By contrast, H3K36me2 was efficiently established within such lowly expressed genes as in intergenic regions, presumably by one or more NSD family members.

Our study shows that KDM2A/KDM2B define the competence of oocytes for pre-implantation development by restricting DNAme acquisition during oocyte growth. Given the lethality of maternal *Kdm2a*/*Kdm2b* mutants during pre-implantation development, we propose that aberrant maternal DNAme is maintained during this period, at least in a partially penetrant manner, reducing expression of zygotically activated genes and of key developmental regulators in a variegating manner between different embryos. In line with this, the extensive oocyte-derived DNAme is not majorly removed after fertilization in zygotes, unlike sperm-derived DNAme, nor at CGIs as measured in four-cell embryos, arguing that global DNAme reprogramming activities in early embryos are restrained in a parent-of-origin-specific manner. Importantly, transcriptional and DNAme profiling in 2- and four-cell embryos, respectively, revealed a strong association between aberrant CGI-promoter DNAme and suppression of maternal allele-specific gene expression. The observed lethality implies haplo-insufficiency in autosomal gene expression in pre-implantation embryos, in which paternal expression is not sufficient to compensate for the loss of maternal expression caused by persistent promoter DNAme. This finding is in line with early lethality reported for mouse embryos with autosomal monosomy.[63] In addition, the aberrant promoter methylation observed in mutant oocytes at over 50 X-linked loci (e.g., *Atrx*[64]) may effectively suppress expression in male and female early embryos and impair their development.

Besides DNMT3A, DNMT1 together with UHRF1 catalyze *de novo* methylation during late oocyte growth, mainly at lowly or non-transcribed genomic regions.[50,51] Most DNMT1 and UHRF1 proteins, however, are localized in the cytoplasm of oocytes, where UHRF1 is essential in regulating cytoskeletal organization of oocytes and for subsequent pre-implantation development.[65] Intriguingly, by sequestering UHRF1 in the oocyte cytoplasm and refraining it from interacting with chromatin, the STELLA protein (DPPA3 and PGC7) protects the oocyte genome from becoming hypermethylated,[52,66] analogously to KDM2A and KDM2B. In $Kdm2a^{KO}Kdm2b^{KO}$ FGOs, the localization of UHRF1 appeared unaltered (Figure S7K). Comparison of CGIs aberrantly methylated in *Kdm2a*/*Kdm2b* versus *Stella*-deficient oocytes revealed only a limited overlap in targets that are atypically methylated by DNMT3A or by DNMT1/UHRF1 (Figures S7L and S7M). Moreover, while aberrant DNMT3A-mediated DNAme drives pre-implantation lethality of $Kdm2a^{matKO}Kdm2b^{matKO}$ embryos, a spindle transfer experiment with MII-stage oocytes demonstrated that the early lethality of $Stella^{matKO}$ pre-implantation embryos was mainly due to faulty cytoplasm of $Stella^{KO}$ oocytes, while hyper-DNAme may have contributed to later-stage lethality.[52,65] Together, our study assigns an essential role to KDM2A and KDM2B in specifying a hypo-DNA-methylated genome in mouse oocytes required for proper embryonic development.

Limitations of the study

The applied CUT&RUN and WGBS assays provide population-based epigenomic information of mouse oocytes and early embryos. Single embryo approaches are needed to investigate possible variation in aberrant DNAme and its impact on allelic gene expression and development between embryos.

RESOURCE AVAILABILITY

Lead contact

Further information and requests for resources and reagents should be directed to Antoine H.F.M. Peters (antoine.peters@fmi.ch).

Materials availability

This study did not generate new, unique reagents. *Kdm2a* and *Kdm2b* conditionally mutant mouse lines are available from Haruhiko Koseki with a completed materials transfer agreement.

Data and code availability

- RNA-seq, WGBS, and CUT&RUN genomic datasets are available at GEO under GSE234968 (https://www.ncbi.nlm.nih.gov/geo/query/acc.cgi?acc=GSE234968).
- Raw imaging data are available at BioStudies (accession code: S-BSST2126).
- This paper does not report original code.
- Any additional information required to reanalyze the data reported in this work paper is available from the lead contact upon request.

ACKNOWLEDGMENTS

We thank B. Knowles, E. Li, and R. Jaenisch for providing *Zp3-cre* transgenic mice, *Dnmt3a*, and *Dnmt1* conditionally deficient mice, respectively. We gratefully acknowledge A. Inoue for sharing the CUT&RUN protocol and S. Henikoff for providing the pAG/Mnase plasmid (Addgene 123461). We thank S. Bourke, J. Eglinger, and L. Gelman (Facility for Advanced Imaging and Microscopy) and the FMI animal facility for excellent assistance. We thank M. Bühler, D. Schübeler, P. de Boer, and laboratory members for critical reading of the manuscript. This research was supported by the Japan Society for the Promotion of Science fellowship (Y.K.K), the Naito fellowship (Y.K.K), the Novartis Research Foundation, the Swiss National Science Foundation (grant numbers 406340_128131, 31003A-172873), and the European Research Council (ERC) under the European Union's Horizon 2020 research and innovation programme (grant agreement ERC-AdG 695288 – Totipotency).

AUTHOR CONTRIBUTIONS

Y.K.K. and A.H.F.M.P. conceived the study. Y.K.K., E.A.O., P.P., and A.H.F.M.P. designed experiments and interpreted data. Y.K.K. performed genetic, cell biology, and genomic experiments. E.A.O. and P.P. performed computational data analyses. T.K. and H.K. provided *Kdm2a* and *Kdm2b* conditional mutant mouse strains. N.V.N. purified protein-AG-MNase. M.B.S. supported computational analyses. S.A.S. assisted and supervised genomic sequencing. A.H.F.M.P. supervised the project and wrote the manuscript with input from all authors.

DECLARATION OF INTERESTS

The authors declare no competing interests.

STAR★METHODS

Detailed methods are provided in the online version of this paper and include the following:

- KEY RESOURCES TABLE
- EXPERIMENTAL MODEL AND STUDY PARTICIPANT DETAILS
- METHOD DETAILS
  - Collection of mouse oocytes and pre- and post-implantation embryos
  - Immunofluorescence staining of oocytes and preimplantation embryos
  - Smart-seq2 RNA-sequencing of growing and fully grown oocytes and two-cell embryos
  - Total RNA sequencing of GOs
  - CUT&RUN of oocytes
  - Whole Genome Bisulfite sequencing (WGBS)
  - Alignment and quantification of RNA-Seq data of oocyte samples
  - Alignment and allelic assignment for RNA-Seq data of two-cell embryo samples
  - Differential expression analysis of RNA-seq datasets for single FGOs
  - Differential expression analysis of RNA-seq datasets for single day 9 and day 14 GOs
  - Differential expression analysis RNA-seq datasets for 2-cell embryos
  - Gene Ontology enrichment analysis
  - Alignment and quality control of WGBS datasets
  - Analysis of CUT&RUN sequencing data
  - Genome arithmetic operations
  - Clustering and heatmaps for chromatin, sequence and expression features
  - Regularized linear regression for chromatin-mark and DNA-methylation-modelling
  - Example commands
  - Data visualization
  - Chromatin and sequence analysis at UCSC-annotated CGIs
- QUANTIFICATION AND STATISTICAL ANALYSIS

SUPPLEMENTAL INFORMATION

Supplemental information can be found online at https://doi.org/10.1016/j.devcel.2025.08.005.

Received: December 6, 2023
Revised: April 15, 2025
Accepted: August 8, 2025
Published: September 2, 2025

REFERENCES

1. Deaton, A.M., and Bird, A. (2011). CpG islands and the regulation of transcription. Genes Dev. *25*, 1010–1022. https://doi.org/10.1101/gad.2037511.
2. Bird, A.P. (1980). DNA methylation and the frequency of CpG in animal DNA. Nucleic Acids Res. *8*, 1499–1504. https://doi.org/10.1093/nar/8.7.1499.
3. Reik, W., Dean, W., and Walter, J. (2001). Epigenetic reprogramming in mammalian development. Science *293*, 1089–1093. https://doi.org/10.1126/science.1063443.
4. Kobayashi, H., Sakurai, T., Imai, M., Takahashi, N., Fukuda, A., Yayoi, O., Sato, S., Nakabayashi, K., Hata, K., Sotomaru, Y., et al. (2012). Contribution of intragenic DNA methylation in mouse gametic DNA methylomes to establish Oocyte-specific heritable marks. PLoS Genet. *8*, e1002440. https://doi.org/10.1371/journal.pgen.1002440.
5. Smallwood, S.A., Tomizawa, S.I., Krueger, F., Ruf, N., Carli, N., Segonds-Pichon, A., Sato, S., Hata, K., Andrews, S.R., and Kelsey, G. (2011). Dynamic CpG island methylation landscape in oocytes and preimplantation embryos. Nat. Genet. *43*, 811–814. https://doi.org/10.1038/ng.864.
6. Rutledge, C.E., Thakur, A., O'Neill, K.M., Irwin, R.E., Sato, S., Hata, K., and Walsh, C.P. (2014). Ontogeny, conservation and functional significance of maternally inherited DNA methylation at two classes of non-imprinted genes. Development *141*, 1313–1323. https://doi.org/10.1242/dev.104646.
7. Bourc'his, D., and Bestor, T.H. (2004). Meiotic catastrophe and retrotransposon reactivation in male germ cells lacking Dnmt3L. Nature *431*, 96–99. https://doi.org/10.1038/nature02886.
8. Dura, M., Teissandier, A., Armand, M., Barau, J., Lapoujade, C., Fouchet, P., Bonneville, L., Schulz, M., Weber, M., Baudrin, L.G., et al. (2022). DNMT3A-dependent DNA methylation is required for spermatogonial stem cells to commit to spermatogenesis. Nat. Genet. *54*, 469–480. https://doi.org/10.1038/s41588-022-01040-z.
9. Gahurova, L., Tomizawa, S.I., Smallwood, S.A., Stewart-Morgan, K.R., Saadeh, H., Kim, J., Andrews, S.R., Chen, T., and Kelsey, G. (2017). Transcription and chromatin determinants of de novo DNA methylation

timing in oocytes. Epigenetics Chromatin *10*, 25. https://doi.org/10.1186/s13072-017-0133-5.

10. Chotalia, M., Smallwood, S.A., Ruf, N., Dawson, C., Lucifero, D., Frontera, M., James, K., Dean, W., and Kelsey, G. (2009). Transcription is required for establishment of germline methylation marks at imprinted genes. Genes Dev. *23*, 105–117. https://doi.org/10.1101/gad.495809.

11. Yano, S., Ishiuchi, T., Abe, S., Namekawa, S.H., Huang, G., Ogawa, Y., and Sasaki, H. (2022). Histone H3K36me2 and H3K36me3 form a chromatin platform essential for DNMT3A-dependent DNA methylation in mouse oocytes. Nat. Commun. *13*, 4440. https://doi.org/10.1038/s41467-022-32141-2.

12. Kaneda, M., Okano, M., Hata, K., Sado, T., Tsujimoto, N., Li, E., and Sasaki, H. (2004). Essential role for de novo DNA methyltransferase Dnmt3a in paternal and maternal imprinting. Nature *429*, 900–903. https://doi.org/10.1038/nature02633.

13. Branco, M.R., King, M., Perez-Garcia, V., Bogutz, A.B., Caley, M., Fineberg, E., Lefebvre, L., Cook, S.J., Dean, W., Hemberger, M., and Reik, W. (2016). Maternal DNA Methylation Regulates Early Trophoblast Development. Dev. Cell *36*, 152–163. https://doi.org/10.1016/j.devcel.2015.12.027.

14. Baubec, T., Colombo, D.F., Wirbelauer, C., Schmidt, J., Burger, L., Krebs, A.R., Akalin, A., and Schübeler, D. (2015). Genomic profiling of DNA methyltransferases reveals a role for DNMT3B in genic methylation. Nature *520*, 243–247. https://doi.org/10.1038/nature14176.

15. Weinberg, D.N., Papillon-Cavanagh, S., Chen, H., Yue, Y., Chen, X., Rajagopalan, K.N., Horth, C., McGuire, J.T., Xu, X., Nikbakht, H., et al. (2019). The histone mark H3K36me2 recruits DNMT3A and shapes the intergenic DNA methylation landscape. Nature *573*, 281–286. https://doi.org/10.1038/s41586-019-1534-3.

16. Dukatz, M., Holzer, K., Choudalakis, M., Emperle, M., Lungu, C., Bashtrykov, P., and Jeltsch, A. (2019). H3K36me2/3 Binding and DNA Binding of the DNA Methyltransferase DNMT3A PWWP Domain Both Contribute to its Chromatin Interaction. J. Mol. Biol. *431*, 5063–5074. https://doi.org/10.1016/j.jmb.2019.09.006.

17. Gong, T., Gu, X., Liu, Y.T., Zhou, Z., Zhang, L.L., Wen, Y., Zhong, W.L., Xu, G.L., and Zhou, J.Q. (2020). Both combinatorial K4me0-K36me3 marks on sister histone H3s of a nucleosome are required for Dnmt3a-Dnmt3L mediated de novo DNA methylation. J. Genet. Genomics *47*, 105–114. https://doi.org/10.1016/j.jgg.2019.12.006.

18. Shirane, K., Miura, F., Ito, T., and Lorincz, M.C. (2020). NSD1-deposited H3K36me2 directs de novo methylation in the mouse male germline and counteracts Polycomb-associated silencing. Nat. Genet. *52*, 1088–1098. https://doi.org/10.1038/s41588-020-0689-z.

19. Xu, Q., Xiang, Y., Wang, Q., Wang, L., Brind'Amour, J., Bogutz, A.B., Zhang, Y., Zhang, B., Yu, G., Xia, W., et al. (2019). SETD2 regulates the maternal epigenome, genomic imprinting and embryonic development. Nat. Genet. *51*, 844–856. https://doi.org/10.1038/s41588-019-0398-7.

20. Stewart, K.R., Veselovska, L., Kim, J., Huang, J., Saadeh, H., Tomizawa, S.I., Smallwood, S.A., Chen, T., and Kelsey, G. (2015). Dynamic changes in histone modifications precede de novo DNA methylation in oocytes. Genes Dev. *29*, 2449–2462. https://doi.org/10.1101/gad.271353.115.

21. Hanna, C.W., Taudt, A., Huang, J., Gahurova, L., Kranz, A., Andrews, S., Dean, W., Stewart, A.F., Colomé-Tatché, M., and Kelsey, G. (2018). MLL2 conveys transcription-independent H3K4 trimethylation in oocytes. Nat. Struct. Mol. Biol. *25*, 73–82. https://doi.org/10.1038/s41594-017-0013-5.

22. Ciccone, D.N., Su, H., Hevi, S., Gay, F., Lei, H., Bajko, J., Xu, G., Li, E., and Chen, T. (2009). KDM1B is a histone H3K4 demethylase required to establish maternal genomic imprints. Nature *461*, 415–418. https://doi.org/10.1038/nature08315.

23. Ooi, S.K.T., Qiu, C., Bernstein, E., Li, K., Jia, D., Yang, Z., Erdjument-Bromage, H., Tempst, P., Lin, S.P., Allis, C.D., et al. (2007). DNMT3L connects unmethylated lysine 4 of histone H3 to de novo methylation of DNA. Nature *448*, 714–717. https://doi.org/10.1038/nature05987.

24. Stäubli, A., and Peters, A.H.F.M. (2021). Mechanisms of maternal intergenerational epigenetic inheritance. Curr. Opin. Genet. Dev. *67*, 151–162. https://doi.org/10.1016/j.gde.2021.01.008.

25. Boulard, M., Edwards, J.R., and Bestor, T.H. (2015). FBXL10 protects Polycomb-bound genes from hypermethylation. Nat. Genet. *47*, 479–485. https://doi.org/10.1038/ng.3272.

26. Farcas, A.M., Blackledge, N.P., Sudbery, I., Long, H.K., McGouran, J.F., Rose, N.R., Lee, S., Sims, D., Cerase, A., Sheahan, T.W., et al. (2012). KDM2B links the polycomb repressive complex 1 (PRC1) to recognition of CpG islands. eLife *1*, e00205. https://doi.org/10.7554/eLife.00205.

27. He, J., Kallin, E.M., Tsukada, Y.I., and Zhang, Y. (2008). The H3K36 demethylase Jhdm1b/Kdm2b regulates cell proliferation and senescence through p15(Ink4b). Nat. Struct. Mol. Biol. *15*, 1169–1175. https://doi.org/10.1038/nsmb.1499.

28. Tsukada, Y.I., Fang, J., Erdjument-Bromage, H., Warren, M.E., Borchers, C.H., Tempst, P., and Zhang, Y. (2006). Histone demethylation by a family of JmjC domain-containing proteins. Nature *439*, 811–816. https://doi.org/10.1038/nature04433.

29. Turberfield, A.H., Kondo, T., Nakayama, M., Koseki, Y., King, H.W., Koseki, H., and Klose, R.J. (2019). KDM2 proteins constrain transcription from CpG island gene promoters independently of their histone demethylase activity. Nucleic Acids Res. *47*, 9005–9023. https://doi.org/10.1093/NAR/GKZ607.

30. Gao, Z., Zhang, J., Bonasio, R., Strino, F., Sawai, A., Parisi, F., Kluger, Y., and Reinberg, D. (2012). PCGF Homologs, CBX Proteins, and RYBP Define Functionally Distinct PRC1 Family Complexes. Mol. Cell *45*, 344–356. https://doi.org/10.1016/j.molcel.2012.01.002.

31. Blackledge, N.P., Farcas, A.M., Kondo, T., King, H.W., McGouran, J.F., Hanssen, L.L.P., Ito, S., Cooper, S., Kondo, K., Koseki, Y., et al. (2014). Variant PRC1 complex-dependent H2A ubiquitylation drives PRC2 recruitment and polycomb domain formation. Cell *157*, 1445–1459. https://doi.org/10.1016/j.cell.2014.05.004.

32. Huo, D., Yu, Z., Li, R., Gong, M., Sidoli, S., Lu, X., Hou, Y., Dai, Z., Kong, Y., Liu, G., et al. (2022). CpG island reconfiguration for the establishment and synchronization of polycomb functions upon exit from naive pluripotency. Mol. Cell *82*, 1169–1185.e7. https://doi.org/10.1016/j.molcel.2022.01.027.

33. Endoh, M., Endo, T.A., Endoh, T., Fujimura, Y.I., Ohara, O., Toyoda, T., Otte, A.P., Okano, M., Brockdorff, N., Vidal, M., and Koseki, H. (2008). Polycomb group proteins Ring1A/B are functionally linked to the core transcriptional regulatory circuitry to maintain ES cell identity. Development *135*, 1513–1524. https://doi.org/10.1242/dev.014340.

34. Blackledge, N.P., Zhou, J.C., Tolstorukov, M.Y., Farcas, A.M., Park, P.J., and Klose, R.J. (2010). CpG Islands Recruit a Histone H3 Lysine 36 Demethylase. Mol. Cell *38*, 179–190. https://doi.org/10.1016/j.molcel.2010.04.009.

35. Xiong, X., Zhang, X., Yang, M., Zhu, Y., Yu, H., Fei, X., Mastuda, F., Lan, D., Xiong, Y., Fu, W., et al. (2022). Oocyte-Specific Knockout of Histone Lysine Demethylase KDM2a Compromises Fertility by Blocking the Development of Follicles and Oocytes. Int. J. Mol. Sci. *23*, 12008. https://doi.org/10.3390/ijms231912008.

36. Posfai, E., Kunzmann, R., Brochard, V., Salvaing, J., Cabuy, E., Roloff, T. C., Liu, Z., Tardat, M., van Lohuizen, M., Vidal, M., et al. (2012). Polycomb function during oogenesis is required for mouse embryonic development. Genes Dev. *26*, 920–932. https://doi.org/10.1101/gad.188094.112.

37. Mei, H., Kozuka, C., Hayashi, R., Kumon, M., Koseki, H., and Inoue, A. (2021). H2AK119ub1 guides maternal inheritance and zygotic deposition of H3K27me3 in mouse embryos. Nat. Genet. *53*, 539–550. https://doi.org/10.1038/s41588-021-00820-3.

38. Veselovska, L., Smallwood, S.A., Saadeh, H., Stewart, K.R., Krueger, F., Maupetit-Méhouas, S., Arnaud, P., Tomizawa, S.I., Andrews, S., and Kelsey, G. (2015). Erratum to: Deep sequencing and de novo assembly of the mouse occyte transcriptome define the contribution of transcription to the DNA methylation landscape. Genome Biol. *16*, 271. https://doi.org/10.1186/s13059-015-0809-8.

39. Zheng, H., Huang, B., Zhang, B., Xiang, Y., Du, Z., Xu, Q., Li, Y., Wang, Q., Ma, J., Peng, X., et al. (2016). Resetting Epigenetic Memory by Reprogramming of Histone Modifications in Mammals. Mol. Cell *63*, 1066–1079. https://doi.org/10.1016/j.molcel.2016.08.032.

40. Zhang, B., Zheng, H., Huang, B., Li, W., Xiang, Y., Peng, X., Ming, J., Wu, X., Zhang, Y., Xu, Q., et al. (2016). Allelic reprogramming of the histone modification H3K4me3 in early mammalian development. Nature *537*, 553–557. https://doi.org/10.1038/nature19361.

41. Hanna, C.W., Huang, J., Belton, C., Reinhardt, S., Dahl, A., Andrews, S., Stewart, A.F., Kranz, A., and Kelsey, G. (2022). Loss of histone methyltransferase SETD1B in oogenesis results in the redistribution of genomic histone 3 lysine 4 trimethylation. Nucleic Acids Res. *50*, 1993–2004. https://doi.org/10.1093/nar/gkac051.

42. Kubo, N., Uehara, R., Uemura, S., Ohishi, H., Shirane, K., and Sasaki, H. (2024). Combined and differential roles of ADD domains of DNMT3A and DNMT3L on DNA methylation landscapes in mouse germ cells. Nat. Commun. *15*, 3266. https://doi.org/10.1038/s41467-024-47699-2.

43. Xu, W., Li, J., Rong, B., Zhao, B., Wang, M., Dai, R., Chen, Q., Liu, H., Gu, Z., Liu, S., et al. (2020). Correction to: DNMT3A reads and connects histone H3K36me2 to DNA methylation. Protein Cell *11*, 230. https://doi.org/10.1007/s13238-019-00678-6.

44. Yuan, G., Ma, B., Yuan, W., Zhang, Z., Chen, P., Ding, X., Feng, L., Shen, X., Chen, S., Li, G., and Zhu, B. (2013). Histone H2A ubiquitination inhibits the enzymatic activity of H3 lysine 36 methyltransferases. J. Biol. Chem. *288*, 30832–30842. https://doi.org/10.1074/jbc.M113.475996.

45. Li, W., Tian, W., Yuan, G., Deng, P., SenGupta, D., Cheng, Z., Cao, Y., Ren, J., Qin, Y., Zhou, Y., et al. (2021). Molecular basis of nucleosomal H3K36 methylation by NSD methyltransferases. Nature *590*, 498–503. https://doi.org/10.1038/s41586-020-03069-8.

46. Brici, D., Zhang, Q., Reinhardt, S., Dahl, A., Hartmann, H., Schmidt, K., Goveas, N., Huang, J., Gahurova, L., Kelsey, G., et al. (2017). Setd1b, encoding a histone 3 lysine 4 methyltransferase, is a maternal effect gene required for the oogenic gene expression program. Development *144*, 2606–2617. https://doi.org/10.1242/dev.143347.

47. Brown, D.A., Di Cerbo, V., Feldmann, A., Ahn, J., Ito, S., Blackledge, N.P., Nakayama, M., McClellan, M., Dimitrova, E., Turberfield, A.H., et al. (2017). The SET1 Complex Selects Actively Transcribed Target Genes via Multivalent Interaction with CpG Island Chromatin. Cell Rep. *20*, 2313–2327. https://doi.org/10.1016/j.celrep.2017.08.030.

48. Yu, C., Fan, X., Sha, Q.Q., Wang, H.H., Li, B.T., Dai, X.X., Shen, L., Liu, J., Wang, L., Liu, K., et al. (2017). CFP1 Regulates Histone H3K4 Trimethylation and Developmental Potential in Mouse Oocytes. Cell Rep. *20*, 1161–1172. https://doi.org/10.1016/j.celrep.2017.07.011.

49. Xu, C., Liu, K., Lei, M., Yang, A., Li, Y., Hughes, T.R., and Min, J. (2018). DNA Sequence Recognition of Human CXXC Domains and Their Structural Determinants. Structure *26*, 85–95.e3. https://doi.org/10.1016/j.str.2017.11.022.

50. Shirane, K., Toh, H., Kobayashi, H., Miura, F., Chiba, H., Ito, T., Kono, T., and Sasaki, H. (2013). Mouse Oocyte Methylomes at Base Resolution Reveal Genome-Wide Accumulation of Non-CpG Methylation and Role of DNA Methyltransferases. PLoS Genet. *9*, e1003439. https://doi.org/10.1371/journal.pgen.1003439.

51. Maenohara, S., Unoki, M., Toh, H., Ohishi, H., Sharif, J., Koseki, H., and Sasaki, H. (2017). Role of UHRF1 in de novo DNA methylation in oocytes and maintenance methylation in preimplantation embryos. PLoS Genet. *13*, e1007042. https://doi.org/10.1371/journal.pgen.1007042.

52. Li, Y., Zhang, Z., Chen, J., Liu, W., Lai, W., Liu, B., Li, X., Liu, L., Xu, S., Dong, Q., et al. (2018). Stella safeguards the oocyte methylome by preventing de novo methylation mediated by DNMT1. Nature *564*, 136–140. https://doi.org/10.1038/s41586-018-0751-5.

53. Kaneda, M., Hirasawa, R., Chiba, H., Okano, M., Li, E., and Sasaki, H. (2010). Genetic evidence for Dnmt3a-dependent imprinting during oocyte growth obtained by conditional knockout with Zp3-Cre and complete exclusion of Dnmt3b by chimera formation. Genes Cells *15*, 169–179. https://doi.org/10.1111/j.1365-2443.2009.01374.x.

54. Kubo, N., Toh, H., Shirane, K., Shirakawa, T., Kobayashi, H., Sato, T., Sone, H., Sato, Y., Tomizawa, S.I., Tsurusaki, Y., et al. (2015). DNA methylation and gene expression dynamics during spermatogonial stem cell differentiation in the early postnatal mouse testis. BMC Genomics *16*, 624. https://doi.org/10.1186/s12864-015-1833-5.

55. Dahl, J.A., Jung, I., Aanes, H., Greggains, G.D., Manaf, A., Lerdrup, M., Li, G., Kuan, S., Li, B., Lee, A.Y., et al. (2016). Broad histone H3K4me3 domains in mouse oocytes modulate maternal-to-zygotic transition. Nature *537*, 548–552. https://doi.org/10.1038/nature19360.

56. Richard Albert, J., Au Yeung, W.K., Toriyama, K., Kobayashi, H., Hirasawa, R., Brind'Amour, J., Bogutz, A., Sasaki, H., and Lorincz, M. (2020). Maternal DNMT3A-dependent de novo methylation of the paternal genome inhibits gene expression in the early embryo. Nat. Commun. *11*, 5417. https://doi.org/10.1038/s41467-020-19279-7.

57. Mukha, D., Fokra, M., Feldman, A., Sarvin, B., Sarvin, N., Nevo-Dinur, K., Besser, E., Hallo, E., Aizenshtein, E., Schug, Z.T., and Shlomi, T. (2022). Glycine decarboxylase maintains mitochondrial protein lipoylation to support tumor growth. Cell Metab. *34*, 775–782.e9. https://doi.org/10.1016/j.cmet.2022.04.006.

58. Kanno, J., Hutchin, T., Kamada, F., Narisawa, A., Aoki, Y., Matsubara, Y., and Kure, S. (2007). Genomic deletion within GLDC is a major cause of non-ketotic hyperglycinaemia. J. Med. Genet. *44*, e69. https://doi.org/10.1136/jmg.2006.043448.

59. Tian, S., Feng, J., Cao, Y., Shen, S., Cai, Y., Yang, D., Yan, R., Wang, L., Zhang, H., Zhong, X., and Gao, P. (2019). Glycine cleavage system determines the fate of pluripotent stem cells via the regulation of senescence and epigenetic modifications. Life Sci. Alliance *2*, e201900413. https://doi.org/10.26508/lsa.201900413.

60. Grosswendt, S., Kretzmer, H., Smith, Z.D., Kumar, A.S., Hetzel, S., Wittler, L., Klages, S., Timmermann, B., Mukherji, S., and Meissner, A. (2020). Epigenetic regulator function through mouse gastrulation. Nature *584*, 102–108. https://doi.org/10.1038/s41586-020-2552-x.

61. Takahashi, Y., Morales Valencia, M., Yu, Y., Ouchi, Y., Takahashi, K., Shokhirev, M.N., Lande, K., Williams, A.E., Fresia, C., Kurita, M., et al. (2023). Transgenerational inheritance of acquired epigenetic signatures at CpG islands in mice. Cell *186*, 715–731.e19. https://doi.org/10.1016/j.cell.2022.12.047.

62. Lerner, A.M., Hepperla, A.J., Keele, G.R., Meriesh, H.A., Yumerefendi, H., Restrepo, D., Zimmerman, S., Bear, J.E., Kuhlman, B., Davis, I.J., and Strahl, B.D. (2020). An optogenetic switch for the Set2 methyltransferase provides evidence for transcription-dependent and -independent dynamics of H3K36 methylation. Genome Res. *30*, 1605–1617. https://doi.org/10.1101/gr.264283.120.

63. Magnuson, T., Debrot, S., Dimpfl, J., Zweig, A., Zamora, T., and Epstein, C.J. (1985). The early lethality of autosomal monosomy in the mouse. J. Exp. Zool. *236*, 353–360. https://doi.org/10.1002/jez.1402360313.

64. Garrick, D., Sharpe, J.A., Arkell, R., Dobbie, L., Smith, A.J.H., Wood, W.G., Higgs, D.R., and Gibbons, R.J. (2006). Loss of Atrx affects trophoblast development and the pattern of X-inactivation in extraembryonic tissues. PLoS Genet. *2*, e58. https://doi.org/10.1371/journal.pgen.0020058.

65. Uemura, S., Maenohara, S., Inoue, K., Ogonuki, N., Matoba, S., Ogura, A., Kurumizaka, M., Yamagata, K., Sharif, J., Koseki, H., et al. (2023). UHRF1 is essential for proper cytoplasmic architecture and function of mouse oocytes and derived embryos. Life Sci. Alliance *6*, e202301904. https://doi.org/10.26508/lsa.202301904.

66. Du, W., Dong, Q., Zhang, Z., Liu, B., Zhou, T., Xu, R.M., Wang, H., Zhu, B., and Li, Y. (2019). Stella protein facilitates DNA demethylation by disrupting the chromatin association of the RING finger-type E3 ubiquitin ligase UHRF1. J. Biol. Chem. *294*, 8907–8917. https://doi.org/10.1074/jbc.RA119.008008.

67. De Vries, W.N., Binns, L.T., Fancher, K.S., Dean, J., Moore, R., Kemler, R., and Knowles, B.B. (2000). Expression of Cre recombinase in mouse oocytes: A means to study maternal effect genes. Genesis *26*, 110–112. https://doi.org/10.1002/(SICI)1526-968X(200002)26:2<110::AID-GENE2>3.0.CO;2-8.

68. Hirasawa, R., Chiba, H., Kaneda, M., Tajima, S., Li, E., Jaenisch, R., and Sasaki, H. (2008). Maternal and zygotic Dnmt1 are necessary and sufficient for the maintenance of DNA methylation imprints during preimplantation development. Genes Dev. *22*, 1607–1616. https://doi.org/10.1101/gad.1667008.

69. Schindelin, J., Arganda-Carreras, I., Frise, E., Kaynig, V., Longair, M., Pietzsch, T., Preibisch, S., Rueden, C., Saalfeld, S., Schmid, B., et al. (2012). Fiji: an open-source platform for biological-image analysis. Nat. Methods *9*, 676–682. https://doi.org/10.1038/nmeth.2019.

70. Picelli, S., Faridani, O.R., Björklund, A.K., Winberg, G., Sagasser, S., and Sandberg, R. (2014). Full-length RNA-seq from single cells using Smart-seq2. Nat. Protoc. *9*, 171–181. https://doi.org/10.1038/nprot.2014.006.

71. Picelli, S., Björklund, A.K., Reinius, B., Sagasser, S., Winberg, G., and Sandberg, R. (2014). Tn5 transposase and tagmentation procedures for massively scaled sequencing projects. Genome Res. *24*, 2033–2040. https://doi.org/10.1101/gr.177881.114.

72. Meers, M.P., Bryson, T.D., Henikoff, J.G., and Henikoff, S. (2019). Improved cut&run chromatin profiling tools. eLife *8*, e46314. https://doi.org/10.7554/eLife.46314.

73. Clark, S.J., Smallwood, S.A., Lee, H.J., Krueger, F., Reik, W., and Kelsey, G. (2017). Genome-wide base-resolution mapping of DNA methylation in single cells using single-cell bisulfite sequencing (scBS-seq). Nat. Protoc. *12*, 534–547. https://doi.org/10.1038/nprot.2016.187.

74. Dobin, A., Davis, C.A., Schlesinger, F., Drenkow, J., Zaleski, C., Jha, S., Batut, P., Chaisson, M., and Gingeras, T.R. (2013). STAR: Ultrafast universal RNA-seq aligner. Bioinformatics *29*, 15–21. https://doi.org/10.1093/bioinformatics/bts635.

75. Carlson, M., and Maintainer, B.P. (2019). TxDb.Mmusculus.UCSC.mm10.knownGene: Annotation package for TxDb object(s). R package version 3.10.0. Bioconductor. https://bioconductor.org/packages/release/data/annotation/html/TxDb.Mmusculus.UCSC.mm10.knownGene.html.

76. Gaidatzis, D., Lerch, A., Hahne, F., and Stadler, M.B. (2015). QuasR: Quantification and annotation of short reads in R. Bioinformatics *31*, 1130–1132. https://doi.org/10.1093/bioinformatics/btu781.

77. Takada, T., Ebata, T., Noguchi, H., Keane, T.M., Adams, D.J., Narita, T., Shin-I, T., Fujisawa, H., Toyoda, A., Abe, K., et al. (2013). The ancestor of extant Japanese fancy mice contributed to the mosaic genomes of classical inbred strains. Genome Res. *23*, 1329–1338. https://doi.org/10.1101/gr.156497.113.

78. McCarthy, D.J., Chen, Y., and Smyth, G.K. (2012). Differential expression analysis of multifactor RNA-Seq experiments with respect to biological variation. Nucleic Acids Res. *40*, 4288–4297. https://doi.org/10.1093/nar/gks042.

79. Cannoodt, R., Saelens, W., Sichien, D., Tavernier, S., Janssens, S., Guilliams, M., Lambrecht, B., De Preter, K.D., and Saeys, Y. SCORPIUS improves trajectory inference and identifies novel modules in dendritic cell development. Preprint at bioRxiv. https://doi.org/10.1101/079509.

80. Alexa, A., and Rahnenführer, J. (2022). topGO: Enrichment Analysis for Gene Ontology. R package version 2.61.1. Bioconductor. https://bioconductor.org/packages/devel/bioc/html/topGO.html#:~:text=Enrichment%20Analysis%20for%20Gene%20Ontology&text=topGO%20package%20provides%20tools%20for,can%20be%20implemented%20and%20applied.

81. Carlson, M. (2019). org.Mm.eg.db: Genome wide annotation for Mouse. R package version 3.21.0. Bioconductor. http://bioconductor.org/packages/release/data/annotation/html/org.Mm.eg.db.html.

82. Krueger, F. (2015). Trim Galore!: A wrapper tool around Cutadapt and FastQC to consistently apply quality and adapter trimming to FastQ files (Babraham Institute).

83. Krueger, F., and Andrews, S.R. (2011). Bismark: A flexible aligner and methylation caller for Bisulfite-Seq applications. Bioinformatics *27*, 1571–1572. https://doi.org/10.1093/bioinformatics/btr167.

84. Krueger, F., and Andrews, S.R. (2016). SNPsplit: Allele-specific splitting of alignments between genomes with known SNP genotypes. F1000Res. *5*, 1479. https://doi.org/10.12688/f1000research.9037.2.

85. Gu, Z., Eils, R., and Schlesner, M. (2016). Complex heatmaps reveal patterns and correlations in multidimensional genomic data. Bioinformatics *32*, 2847–2849. https://doi.org/10.1093/bioinformatics/btw313.

86. Wickham, H. (2016). ggplot2: Elegant Graphics for Data Analysis (Springer). https://doi.org/10.1007/978-3-319-24277-4.

STAR★METHODS

KEY RESOURCES TABLE

REAGENT or RESOURCE	SOURCE	IDENTIFIER
**Antibodies**		
Anti-Ubiquityl-Histone H2A (Lys119) (D27C4) XP Rabbit mAb	Cell Signaling Technology	Cat#8240; RRID: AB_10891618
Anti-Tri-Methyl-Histone H3 (Lys27) (C36B11) Rabbit mAb	Cell Signaling Technology	Cat#9733; RRID: AB_2616029
Anti-dimethyl Histone H3 (Lys36) Mouse mAb	MBL International	Cat#MABI0332; RRID: AB_11142494
Anti-5-Methylcytosine (33D3) Mouse mAB	Eurogentec	Cat#BI-MECY_100
Anti-KDM2A antibody	Abcam	Cat#Ab191387; RRID: AB_2928955
Anti-KDM2B antibody	Dr. Haruhiko Koseki, RIKEN Center for Integrative Medical Sciences, Yokohama, Japan.	N/A
Anti-Histone H3 (tri methyl K36) antibody - ChIP Grade	Abcam	Cat#Ab9050; RRID: AB_306966
Donkey anti-Mouse IgG (H+L) Highly Cross-Adsorbed Secondary Antibody, Alexa Fluor 488	Thermo Fisher Scientific	Cat#A-21202; RRID: AB_141607
Donkey anti-Rabbit IgG (H+L) Highly Cross-Adsorbed Secondary Antibody, Alexa Fluor 488	Thermo Fisher Scientific	Cat#A-21206; RRID: AB_2535792
Donkey anti-Mouse IgG (H+L) Highly Cross-Adsorbed Secondary Antibody, Alexa Fluor 568	Thermo Fisher Scientific	Cat#A-10037; RRID: AB_2534013
Donkey anti-Rabbit IgG (H+L) Highly Cross-Adsorbed Secondary Antibody, Alexa Fluor 568	Thermo Fisher Scientific	Cat#A-10042; RRID: AB_2534017
Donkey anti-Mouse IgG (H+L) Highly Cross-Adsorbed Secondary Antibody, Alexa Fluor 647	Thermo Fisher Scientific	Cat#A-31571; RRID: AB_162542
Donkey anti-Rabbit IgG (H+L) Highly Cross-Adsorbed Secondary Antibody, Alexa Fluor 647	Thermo Fisher Scientific	Cat#A-31573; RRID: AB_2536183
**Chemicals, peptides, and recombinant proteins**		
Pregnant Mare Serum Gonadotropin (PMSG)	MSD	Cat#A207A01
Human Chorionic Gonadotoropin (hCG)	MSD	Cat#A201A01
CARD HyperOva Superovulation Reagent for Mice	CARD	Cat#KYD-010-EX-X5
Human Tubal Fluid medium (HTF)	Merck Millipore	Cat#MR-070-D
M2 medium	Merck	Cat#M7167
KSOM	Merck Millipore	Cat#MR-106-D
TrypLE Express Enzyme (1x)	Gibco	Cat#12604013
Mineral oil	Sigma	Cat#M5310
Albumin	Sigma	Cat#A-3311
Milrinone	Sigma-Aldrich	Cat#475840
Tween 20	Sigma-Aldrich	Cat#P2287-100ML
Phosphate-buffered saline (PBS)	Lonza	Cat#11629980
Bovine Serum Albumin (BSA)	New England Biolabs	Cat#B9000S
Tyrode's Solution, Acidic	Sigma-Aldrich	Cat#T1788
VECTASHIELD Antifade Mounting Medium with DAPI	Vector Laboratories	Cat#H-1200-10
VECTASHIELD PLUS Antifade Mounting Medium with DAPI	Vector Laboratories	Cat#H-2000-10
SUPERase-In Rnase inhibitor	Thermo Fisher Scientific	Cat#AM2696
Superscript-II	Thermo Fisher Scientific	Cat#18064014
dNTPs mix (10mM)	Promega/Catalys	Cat#U1515
Betaine Solution	Sigma-Aldrich	Cat#B0300-1VL

(*Continued on next page*)

Continued

REAGENT or RESOURCE	SOURCE	IDENTIFIER
Magnesium Chloride	US Biochemical	Cat#78641
Phusion High-Fidelity DNA Polymerase	Thermo Fisher Scientific	Cat#F530L
Triton X-100	Sigma-Aldrich	Cat#T9284
UltraPure SDS Solution, 10%	Thermo Fisher Scientific	Cat#24730-020
Poly(vinyl alcohol)	Sigma-Aldrich	Cat#P8136
KAPA HiFi HotStart Ready mix	Roche	Cat#KK2601
AMPure XP beads	Beckman Coulter	Cat#A63881
Nuclease-free water (not DEPC-Treated)	Ambion / Thermo Fisher	Cat#A9937
Buffer EB	QIAGEN	Cat#19086
ERCC RNA Spike-In Mix	Thermo Fisher Scientific	Cat#4456740
Tn5-transposase	Picelli et al.[71]; Self-purified protein	N/A
Buffer RLT	QIAGEN	Cat#79216
Klenow Fragment (3′–5′ exo-)	Enzymatics	Cat#P7010-LC-L
dNTPs mix (10mM)	Roche	Cat#4638956001
Exonuclease I	New England Biolabs	Cat#M0293S
Glycogen	Invitrogen	Cat#10814010
RNase A	Roche	Cat#10109169001
BioMag Plus Concanavalin A	Polysciences, Inc	Cat#86057-3
Digitonin (5%)	Thermo Fisher Scientific	Cat#BN2006
Carrier RNA	QIAGEN Epitect Bisulfite Kit	Cat#59104
Phase Lock Gel Heavy	Quantabio	Cat#733-2478
UltraPure™ Phenol:Chloroform:Isoamyl Alcohol (25:24:1, v/v)	Thermo Fisher Scientific	Cat#15593031
PowerUp SYBR Green Master Mix	Thermo Fisher Scientific	Cat#A25741
pA/G-MNase	Meers et al.[72]; Self-purified protein	N/A
Critical commercial assays		
EZ DNA Methylation-Direct Kit	Zymo Research	Cat#D5020
PureLink micro Kit	Thermo Fischer Scientific	Cat#K310050
Single Cell RNA Purification Kit	NORGEN	Cat#51800
Nextera XT Index Kit v2 Set A (96 indexes, 384 samples)	illumina	Cat# FC-131-2001
Nextera XT Index Kit v2 Set B (96 indexes, 384 samples)	illumina	Cat# FC-131-2002
IDT for Illumina DNA/RNA UD Indexes	illumina	Cat#20027213
NEBNext Multiplex Oligos for Illumina (Index Primer Set1)	illumina	Cat#E7335
NEBNext Multiplex Oligos for Illumina (Index Primer Set2)	illumina	Cat#E7500
NEBNext Multiplex Oligos for Illumina (Index Primer Set3)	illumina	Cat#E7710
NEBNext Multiplex Oligos for Illumina (Index Primer Set4)	illumina	Cat#E7730
KAPA HiFi HotStart PCR Kit	Roche	Cat#KK2502
NEBNext Ultra II DNA Library Prep Kit for Illumina	illumina	Cat#E7645L
Deposited data		
RNA-Seq, WBGS, Cut&Run datasets for oocytes and embryos upon various genetic perturbations of *Kdm2a*, *Kdm2b*, *Ring1 and Rnf2*.	This study	GEO: GSE234968

(*Continued on next page*)

Continued

REAGENT or RESOURCE	SOURCE	IDENTIFIER
**Experimental models: Organisms/strains**		
Mouse: C57BL/6J	Janvier Labs	RRID:IMSR_JAX:000664
Mouse: JF1/MsJ	The Jackson Laboratory	Strain#003720; RRID:IMSR_JAX:003720
Mouse: Kdm2a$^{flox-JmJ/flox-JmJ}$: B6.Cg-Kdm2a.tm1Hko	Dr. Haruhiko Koseki, RIKEN Center for Integrative Medical Sciences, Yokohama, Japan.	N/A
Mouse: Kdm2b$^{flox-JmJ/flox-JmJ}$: B6.Cg-Kdm2b.tm1Hko	Dr. Haruhiko Koseki, RIKEN Center for Integrative Medical Sciences, Yokohama, Japan.	N/A
Mouse: Kdm2b$^{flox-CxxC/flox-CxxC}$: B6.Cg-Kdm2b(CxxC).tm2Hko	Dr. Haruhiko Koseki, RIKEN Center for Integrative Medical Sciences, Yokohama, Japan.	N/A
Mouse: C57BL/6-Tg(Zp3-cre)93Knw/J	Dr. Barbara Knowles, The Jackson Laboratory, Bar Harbor, USA	Strain#003651; RRID:IMSR_JAX:003651
Mouse: Dnmt1$^{flox/flox}$: B6;129S-Dnmt1tm1Jae/J	Dr. Rudolf Jaenisch, Whitehead Institute, Cambridge, USA	RRID:IMSR_JAX:002123
Mouse: Dnmt3a$^{flox/flox}$: TgH(Dnmt3a-cko)Enl	Dr. En Li, Novartis, Cambridge, USA	N/A
**Oligonucleotides**		
TSO (template-switching oligos): AAGCAGT GGTATCAACGCAGAGTACATrGrG+G	Picellli et al.[70]	N/A
Oligo-dT30VN: AAG CAG TGG TAT CAA CGC AGA GTA CTT TTT TTT TTT TTT TTT TTT TTT TTT TTT TVN	Picellli et al.[70]	N/A
ISPCR primers: AAGCAGTGGTATCAACGCAGAGT	Picelli et al.[70]	N/A
Preamp primer for WGBS: 5′-[Btn]TGACTGG AGTTCAGACGTGTGCTCTTCCGATCTNNNNNN*N	Smallwood et al.[5]	N/A
Adapter primer 2 for WGBS: 5′-ACACTCTTTCC CTACACGACGCTCTTCCGATCTNNNNNN*N	Smallwood et al.[5]	N/A
**Recombinant DNA**		
pAG/MNase plasmid	Meers et al.,[72] Addgene	RRID:Addgene123461
**Software and algorithms**		
Fiji	Schindelin et al.[69]	http://fiji.sc; RRID:SCR_002285
R	R Core Team	https://www.R-project.org/; RRID:SCR_001905
edgeR	McCarthy et al.[78]	https://bioconductor.org/packages/release/bioc/html/edgeR.html; RRID:SCR_012802
FastQC (v0.11.8)	Babraham Bioinformatics, Krueger[82]	https://www.bioinformatics.babraham.ac.uk/projects/fastqc/
QuasR	Gaidatzis et al.[76]	https://bioconductor.org/packages/release/bioc/html/QuasR.html
Samtools	Samtools	http://www.htslib.org
SRA-Toolkit	NIH	https://hpc.nih.gov/apps/sratoolkit.html
STAR aligner (v2.5.0a)	Dobin et al.[74]	https://github.com/alexdobin/STAR
topGO (v2.48.0)	Alexa and Rahnenfuhrer[80]	https://bioconductor.org/packages/release/bioc/html/topGO.html
TxDb.Mmusculus.UCSC.mm10.knownGene (version 3.2.2)	Carlson and Maintainer[75]	https://bioconductor.org/packages/3.2/data/annotation/html/TxDb.Mmusculus.UCSC.mm10.knownGene.html
org.Mm.eg.db (version 3.15.0)	Carlson[81]	https://bioconductor.org/packages/3.15/data/annotation/html/org.Mm.eg.db.html

(*Continued on next page*)

Continued

REAGENT or RESOURCE	SOURCE	IDENTIFIER
splines (v3.5.1)	R Core Team	https://www.R-project.org/
TrimGalore(v0.6.2)	Babraham Bioinformatics, Krueger[82]	https://github.com/FelixKrueger/TrimGalore/releases/tag/0.6.2
Bismark(v0.22.3)	Babraham Bioinformatics, Krueger and Andrews[83]	https://github.com/FelixKrueger/Bismark/releases/tag/0.22.3
ggplot2	Wickham[86]	https://ggplot2.tidyverse.org; RRID:SCR_014601
ComplexHeatmap	Gu et al.[85]	https://bioconductor.org/packages/release/bioc/html/ComplexHeatmap.html
SNPsplit (v0.6.0)	Krueger and Andrews[84]	https://github.com/FelixKrueger/SNPsplit

EXPERIMENTAL MODEL AND STUDY PARTICIPANT DETAILS

To generate maternally conditionally mutated mice, *Kdm2a*$^{flox\text{-}JmJ/flox\text{-}JmJ}$ (with exon 8 floxed, parental ES cell line described[29]), *Kdm2b*$^{flox\text{-}JmJ/flox\text{-}JmJ}$ (with exons 7 and 8 floxed, parental ES cell line described[29]) mice and *Kdm2a*$^{flox\text{-}JmJ/flox\text{-}JmJ}$; *Kdm2b*$^{flox\text{-}CxxC/flox\text{-}CxxC}$ (with exon 13 floxed[31]) mice were crossed with mice carrying the Zp3-cre recombinase transgene, which excises floxed exons in GOs.[67] *Kdm2a*$^{flox\text{-}JmJ/flox\text{-}JmJ}$; *Kdm2b*$^{flox\text{-}JmJ/flox\text{-}JmJ}$; *Zp3-cre* female mice produced oocytes deficient for KDM2A and KDM2B proteins. *Kdm2b*$^{flox\text{-}CxxC/flox\text{-}CxxC}$ mutation produces in-frame *Kdm2b* transcripts encoding a KDM2B protein lacking CxxC-motif binding domain. *Kdm2a*$^{flox\text{-}JmJ/flox\text{-}JmJ}$; *Zp3-cre, Kdm2b*$^{flox\text{-}JmJ/flox\text{-}JmJ}$; *Zp3-cre*, and *Kdm2b*$^{flox\text{-}CxxC/flox\text{-}CxxC}$; *Zp3-cre* (single-gene mutation) mice were generated by isolating them from double-gene mutation mice. Triple-gene mutations with *Dnmt1* or *Dnmt3a* were generated by crossing double-gene mutation mice with *Dnmt1*$^{flox/flox}$ or *Dnmt3a*$^{flox/flox}$ mice, respectively.[53,68] All mutant mice were held on a C57BL/6J genetic background.

We refer to *ctrl* mice as genetically modified mice that we generated in experimental crosses but that do not harbor the *Zp3-cre* transgene. We refer to *wt* mice as genetically non-modified mice that we used in this study as sperm donors or that have been used in various published epigenomic studies as sources of gametes.

All experiments were performed in accordance with Swiss animal protection laws (licenses 2569, 2670, 3183, Gesundheitsdepartement Kanton Basel-Stadt, Veterinäramt, Switzerland) and institutional guidelines.

METHOD DETAILS

Collection of mouse oocytes and pre- and post-implantation embryos

GOs were collected from 9.0 or 14.0 days old mice. Ovaries were dissociated in TrypLE Express Enzyme (1x) (Gibco; 12604013). Isolated oocytes were washed in M2 medium supplemented with Milrinone (25 μM, Sigma-Aldrich; 475840).

To collect fully grown germinal vesicle oocytes (FGOs) from 4- to 20-week-old female mice, 100 μl of Hyper Ova (CARD; KYD-010-EX-X5) or 5 I.U. of pregnant mare serum gonadotropin (PMSG, MSD; A207A01) were injected 46-52 h before the collection. Ovaries were dissected out in M2 medium (Merck; M7167) supplemented with Milrinone (25 μM) and cumulus cells were removed by mouth-pipetting using thin glass needles.

To collect MII oocytes, 7- to 20-week-old female mice were super-ovulated by injecting 5 I.U. of PMSG and 5 I.U. of human chorionic gonadotropin (hCG, MSD; A201A01).

For the assessment of preimplantation embryonic development and sample collection for the genomic experiments, we performed *in vitro* fertilization (IVF) to synchronize fertilization timing across experimental conditions. To generate hybrid strain embryos, we used JF1/MsJ strain males (The Jackson Laboratory; 003720). Spermatozoa were capacitated in Human Tubal Fluid medium (HTF) (Merck Millipore; MR-070-D) supplemented with 10 mg/ml Albumin (Sigma; A-3311) for 1-1.5 h prior to insemination and used for IVF performed in HTF with Albumin. The starting time of insemination was designated as 0 hpf (hours post-fertilization). At 4 hpf, eggs were transferred to KSOM medium (Millipore; MR-106-D) and the number of ovulated oocytes was counted. The formation of two pronuclei was visually confirmed under the microscopy at 6 hpf. Fertilized eggs were cultured in KSOM medium covered with mineral oil (Sigma; M5310) at 37°C with 5% CO_2 and 5% O_2 air. Preimplantation embryo development was observed at embryonic developmental days e1.25, e2.25, e3.25, e4.25 and e5.0 or at embryonic days e1.5, e2.5, e3.5 and e4.5 after IVF. For collecting *Kdm2a*$^{flox\text{-}JmJ/flox\text{-}JmJ}$; *Kdm2b*$^{flox\text{-}CxxC/flox\text{-}CxxC}$ maternally deficient 2-cell embryos for smart-seq2 RNA sequencing, embryos were generated by Intra Cytoplasmic Sperm Injection (ICSI) using JF1/MsJ spermatozoa to prevent the contamination of RNA from multiple sperm strongly attached to the blastomere surface, which can occur after IVF.

To begin with genomics and immunostaining experiments, oocytes and preimplantation embryos were first treated with acidic Tyrode's solution (Sigma-Aldrich; T1788) supplemented with 0.01% polyvinyl alcohol (Sigma-Aldrich; P8136) to remove the zona pellucida and then washed in M2 medium and in PBS supplemented with 0.01-0.05% PVA.

For collecting samples for smart-seq2 and WBGS, we first removed the zona pellucida from GOs, FGOs, late two-cell embryos (at 30 hpf) and morphological four-cell embryos, and then lysed them in smart-seq2 lysis buffer for smart-seq2 or RLT plus buffer (QIAGEN) for WGBS.

To assess post-implantation development, maternally deficient/mutated female mice and corresponding control female mice were mated with *wt* C57BL/6J (JANVIER LABS) male mice. Plug formation was checked the next morning, with noon of that day designated as e0.5. The post-implantation developmental outcome was examined at e9.5.

Immunofluorescence staining of oocytes and preimplantation embryos

After removing the zona pellucida, GOs, FGOs and embryos were washed in PBS supplemented with 0.05% PVA (PBS-PVA). Fixation was done in 4% paraformaldehyde (PFA) in PBS-PVA at room temperature for 15 min. After washing in PBS-PVA three times, samples were permeabilized in 0.5% Triton X-100/ PBS at room temperature for 15 min followed by washing in PBS containing 0.1% Tween-20 (Sigma-Aldrich; P2287) (PBS-T). For the staining of 5mC, after permeabilization, samples were treated with 4 N HCl for 10 min followed by incubation in 100 mM Tris-HCl (pH 8.0) for 10 min, both at room temperature. 2% BSA (w/v) or 5% normal goat serum in PBS-T was used for blocking. The incubation with primary antibodies diluted in PBS-T with 1% BSA or 5% normal goat serum was done at 4°C overnight. The following primary antibodies were used: anti-H2AK119ub1 (1:20,000; Cell Signaling Technology; 8240), anti-H3K27me3 (1:15,000; Cell Signaling Technology; 9733), anti-Kdm2a (1:500; Abcam; ab191387), anti-H3K36me2 (1:500; MBL International; MABI0332), anti-H3K36me3 (1:1,000; Abcam; ab9050), anti-5mC (1:500; Eurogentec; BI-MECY-100). After washing out the primary antibodies with PBS-T, the secondary antibody incubation was performed in a light-proof box at room temperature for 2 h.

Secondary antibodies (Thermo Fischer Scientific) were Alexa Fluor (AF) 488 donkey anti-mouse IgG, AF488 donkey anti-rabbit IgG, AF568 donkey anti-mouse IgG, AF568 donkey anti-rabbit IgG, AF647 donkey anti-mouse IgG and AF647 donkey anti-rabbit IgG at 1:1,000 dilution in PBS-T. After washing three times in PBS-T, samples were mounted in Vectashield Antifade Mounting Medium with DAPI (Vector Laboratories; H-1200, H-2000) on glass slides. Z-stack fluorescent images (0.33 μm per z-step) were acquired by Axio Imager M2 (ZEISS) combined with a Yokogawa CSU W1 Dual camera T2 spinning disk confocal scanning unit (YOKOGAWA). Projection image processing, signal intensity and area size quantifications were done using Fiji software.[69] The signal intensity within nuclei of each Z-stack plane was calculated and normalized by the DAPI positive area size. In box plots, whiskers extend to data points that are less than 1.5 x interquartile range away from the 1st/3rd quartile.

Smart-seq2 RNA-sequencing of growing and fully grown oocytes and two-cell embryos

GOs, FGOs and genetically hybrid two-cell embryos were prepared as described above. Libraries were prepared by the Smart-seq2 protocol[70] with some modifications. For each genotype, we prepared 20-25 libraries, each from one individual FGO or embryo. After the removal of the zona pellucida as described above, samples were washed in PBS (Lonza; 11629980) supplemented with 0.01% PVA (PBS-PVA) once and lysed in 4 μl of smart-seq2 lysis buffer composed of 1.8 μl of 0.2% Triton-X 100 (Sigma-Aldrich; T9284), 0.1 μl of SUPERase IN RNase inhibitor (Invitrogen; AM2694), 1 μl of 10 μM Oligo-dT primer (5’-AAG CAG TGG TAT CAA CGC AGA GTA CTT TTT TTT TTT TTT TTT TTT TTT TTT TTT TVN; Microsynth AG), 1 μl of dNTP mix (10 mM each, Promega; U1515), 0.1 μl of ERCC RNA Spike-In Mix (1:8x10^5 dilution, Thermo Fischer Scientific; 4456740) in 8-well strips or 96-well plate (one sample per one well). Samples were immediately frozen on dry ice and kept in a -80 °C freezer for long-term storage. After thawing, lysed samples were denatured at 72°C for 3 min and quickly chilled on ice. Reverse transcription mix composed of 0.5 μl of SuperScript II reverse transcriptase (Thermo Fischer Scientific, 18064014), 0.25 μl of SUPERase IN RNase inhibitor, 2 μl of Superscript II first-strand buffer, 0.5 μl of 100 mM DTT (in SuperScript II reverse transcriptase kit), 2 μl of 5 M Betaine (Sigma; B0300-1VL), 0.06 μl of 1 M MgCl2, 0.1 μl of 100 μM template-switching oligos (TSOs) (5’AGCAGTGGTATCAACGCAGAGTACATrGrG+G–3’; EXIQON) was added to samples to obtain a total volume 10 μl and the reverse transcription was performed in PCR machine. Next, PCR pre-amplification was performed by adding 12.5 μl of KAPA HiFi HotStart Ready Mix (KAPA Biosystems, KK2602) and 0.25 μl of 10 μM ISPCR primers (5’-AAGCAGTGGTATCAACGCAGAGT; Mycrosynth AG) in total volume of 25 μl. The pre-amplification PCR cycle numbers were 14-16 for GOs and FGOs and 16 for two-cell embryos. Half of amplified cDNA was purified with AMPure XP beads (sample to beads ratio 1:1, Beckman; A63881) and eluted in 15 μl of Buffer EB (QIAGEN). 1 ng of pre-amplified cDNA was used for tagmentation reaction (7 min @ 55°C) using Tn5 tagmention mix (1x TAPS-DMF buffer, self-purified Tn5-tagmentase (final dilution 1:1,200))[71] in total volume of 20 μl. Reactions were stopped by adding 5 μl of 0.2% SDS and kept at 25°C for 7 min. Adapter-ligated fragment amplification was done using Nextera XT index kit v2 (Illumina) in total volume 50 μl (10 μl of Phusion HF Buffer, 2 U of Phusion High Fidelity DNA Polymerase (Thermo Fischer Scientific; F530L), 1.5 μl of dNTP mix (10 mM each)) with 9-10 cycles of PCR. Library DNA was purified by AMPure XP beads (sample to beads ratio 1:1) and eluted in Buffer EB. Sequencing was performed on an Illumina HiSeq2500 machine with single-end 75 bp read length or NovaSeq6000 machine with paired-end 2x50 bp read length (Illumina).

Total RNA sequencing of GOs

GOs were isolated from the d14.0 mice. 60-80 oocytes were pooled in 100 μl of Buffer RL and frozen. 4 and 3 biological replicates were prepared for *ctrl* and *Kdm2a^{KO}Kdm2b^{KO}*, respectively. RNA was purified by using Single Cell RNA Purification Kit (NORGEN; 51800). Libraries were prepared according to Illumina Stranded total RNA-seq protocol with Illumina IDT DNA/RNA UDI indexes. Sequencing was performed on NovaSeq6000 machine with paired-end 2x50bp read length (Illumina).

CUT&RUN of oocytes

CUT&RUN libraries were prepared as previously described.[72] 300 to 500 FGOs were used for H2AK119u1 and 200 FGOs were used for H3K36me2 and H3K36me3 for each Cut and Run library. For each genotype and histone PTM, at least two independent libraries were prepared. The antibodies were rabbit anti-H2AK119ub1 (1:100; Cell Signaling Technology; 8240), mouse anti-H3K36me2 antibody (1:100; Cosmo Bio; MABI0332) and anti-H3K36me3 (1:100; Abcam; ab9050). Self-purified Protein AG-MNase (pDNA was from Addgene; 123461) was used. CUT&RUN libraries were prepared using NEBNext Ultra II DNA Library Prep Kit for Illumina (NEB; E7645L) and sequenced on NextSeq500 (paired-end, 2x75 bp) or NovaSeq6000 (paired-end 2x50bp).

Whole Genome Bisulfite sequencing (WGBS)

FGOs and genetically hybrid morphological four-cell embryos were prepared as described above. WGBS library preparation was performed as described previously[73] with modifications. For bisulfite conversion, EZ DNA Methylation-Direct Kit (Zymo Research; D5020) was used. After zona pellucida removal, we collected 13 pools of 10 FGOs per genotype and 7 pools of 10 control four-cells or 10 pools of 10 maternally mutant four-cells by putting one pool in a single well of 8-well strips containing 2.5 μl of Buffer RLT (QIAGEN; 79216). Samples were stored in -80°C freezer. After thawing, 7.5 μl of Nuclease Free Water (Invitrogen; AM9937) and 65 μl of CT conversion reagent (EZ DNA Methylation-Direct Kit; Zymo Research D5020) were added and samples were incubated in a PCR machine (98°C 8 min, 65°C 180 min). DNA was purified by using PureLink micro kit (Thermo Fischer Scientific; K310050). DNA bound to the purification columns was washed by wash buffer (PureLink micro kit) once, 100 μl of M-Desulphonation buffer (EZ DNA-Methylation kit) was applied on the columns and the incubation was done at room temperature for 15 min to complete the CT-conversion. DNA on the columns was further washed twice and then eluted with the DNA strand synthesis mix (4 μl of Blue Buffer (Enzymatics), 1.6 μl of dNTP (10 mM each) (Roche; 4638956001), 1.6 μl of 20 μM Preamp primer (5'-[Btn] TGACTGGAGTTCAGACGTGTGCTCTTCCGATCTNNNNN*N, SIGMA) and 32.8 μl of Nuclease free water). DNA mixture was denatured at 65°C for 3 min and quickly chilled. Then 1 μl of Klenow Fragment (3'-5' exo-) (Enzymatics; P7010-LC-L) was added and the strand synthesis reaction was done in the PCR machine with the following program (4°C for 5 min, 4°C rising to 37°C with increasing 1°C every 15 sec, 37°C for 30 min, 4°C pause). Another 4 rounds of the strand synthesis reaction were performed, in that synthesized DNA from the previous PCR cycle was denatured at 95°C for 1 min and quickly chilled, then added 2.4 μl of master mix (0.25 μl of 10x Blue Buffer, 0.1 μl of dNTP, 1 μl of 20 μM Preamp primer, 0.4 μl of Klenow Fragment (3'-5' exo-) and 0.65 μl of water) before the strand synthesis PCR. Strand synthesized DNA was treated with 40 U of Exonuclease I (NEB; M0293S). DNA was purified by AMPure XP beads (sample to beads ratio 0.8:1) and eluted with the 2nd strand synthesis mix (5 μl of 10x Blue Buffer, 2 μl of dNTPs (10 mM each), 2 μl of 20 μM Adapter primer 2 (5'-ACACTCTTTCCCTACACGACGCTCTTCCGATCTNNNNN*N, SIGMA) and 39 μl of water). DNA was denatured at 95°C for 45 sec and quickly chilled. Then 2 μl of Klenow fragment was added and amplification incubation was done with the same program as the first strand synthesis. KAPA HiFi HotStart PCR Kit (Roche; KK2502) was used for indexing-amplification with NEBNext Multiplex Oligos for Illumina (NEB) by 10-12 PCR cycles. Libraries were purified by AMPure XP beads (sample to beads ratio 0.8:1) and eluted in Buffer EB. WGBS libraries were sequenced on NextSeq with single-end 75 bp read length (Illumina).

Alignment and quantification of RNA-Seq data of oocyte samples

RNA-Seq datasets were aligned to the Mus musculus genome assembly (GRCm38/mm10 Dec. 2011) as single-end (Smart-Seq2 polyA datasets, Figures S2A, S2B, S5C, and S7A–S7D) or paired-end (total random primed datasets, Figure S5D) using STAR[74] with parameters "-outFilterMultimapNmax 300 -outMultimapperOrder Random -outSAMmultNmax 1 -alignIntronMin 20 -alignIntronMax 1000000". Expression quantification for genes in Bioconductor annotation package TxDb.Mmusculus.UCSC.mm10.knownGene (version 3.2.2)[75] was done using QuasR R package[76] selecting only uniquely mapped reads (mapqMin=255). RPKM values for genes were calculated by normalizing exonic read counts to total exonic length of each gene and total number of reads mapping to all exonic regions in each library. RPKM values were log2 transformed using formula log2(RPKM + psc) – log2(psc) where pseudo-count psc was set to 0.1.

Alignment and allelic assignment for RNA-Seq data of two-cell embryo samples

Smart-seq2 RNA-Seq samples for hybrid Bl6 x JF1 F1 2-cell embryos were separately aligned to Bl6 and JF1 genomes obtained by incorporating JF1 single-nucleotide polymorphisms (SNPs) into reference mm10 genome using previously published SNP table.[77] RNA-seq reads were categorized as maternal (Bl6), paternal (JF1) or undefined based on minimal number of mismatches in alignments to both genomes. Total number of maternal and paternal reads was used as library size for calculating RPKM values and differential expression analysis.

Differential expression analysis of RNA-seq datasets for single FGOs

Genes with at least 1 read per million in at least 3 samples were included in the statistical analysis of differential expression. edgeR[78] was used for statistical analysis of differential gene expression between *Kdm2a*^{KO}*Kdm2b*^{KO}, *Kdm2a*^{KO}*Kdm2b*$^{\Delta CxxC}$, *Kdm2b*$^{\Delta CxxC}$ and respective *ctrl* FGOs. Generalized Linear Model was fit using genotypes as covariates. Statistical significance was estimated using log-likelihood tests and the Benjamini-Hochberg method was used to correct for multiple testing.

Differential expression analysis of RNA-seq datasets for single day 9 and day 14 GOs

Principal component analysis for transcriptomes of single day 9 and day 14 GOs revealed strong dependence on oocyte diameter which explains 16% of variance (Figure S5C). To take this variation into account we included basis functions for natural splines into a generalized linear model. Analysis for day 9 and day 14 GOs was performed separately, and model matrix was constructed by 1) including oocyte genotypes and 2) including basis functions for natural splices with 3 components generated by ns function in R package splines (version 3.5.1) using oocyte diameters as knots. More explicitly, design matrix for GLM was generated using model.matrix function with formula $\sim 0 + \text{genotype} + \text{ns}(d,3)$, where d is a diameter of each single oocyte. To control possible over-fitting, we performed the same analysis using randomly shuffled oocyte diameters.

Differential expression analysis RNA-seq datasets for 2-cell embryos

To consider possible developmental delays due to random experimental factors or indirect effects of maternal depletion of *Kdm2a* and *Kdm2b* we additionally profiled gene expression in single *ctrl* embryos at early and late 2-cell stages (Figures S7A–S7D), which served as a timing control and allowed us to study effects of maternal genetic mutation of *Kdm2a* and *Kdm2b* in the context of gene expression changes which normally occur during maternal-to-zygotic transition in 2-cell embryos. Pseudotime for each embryo was estimated using R package SCORPIUS (v1.0.8)[79] using read counts for both exonic and intronic regions of genes and removing genes in chrX and chrY as well as imprinted genes annotated in geneimprint website (https://geneimprint.com/site/genes-by-species.Mus+musculus).

To consider possible effects of embryo sexes on gene expression we identified sex of each embryo using proportions of reads mapping to chrX and chrY.

Genes with at least 1 read per million in at least 3 samples were included in the statistical analysis of differential expression which was done using edgeR package.[78] Construction of a model matrix for generalized linear model (GLM) was done by 1) including interaction between genotypes and embryo sexes as covariates and 2) including basis functions for natural splines with 3 components generated by ns function in R package splines (version 3.5.1) using pseudotime as knots to regress out effects of possible developmental delays. More explicitly, design matrix for GLM was generated using model.matrix function with formula $\sim 0 + \text{genotype:sex} + \text{ns(PsT,3)}$. To control possible overfitting by splines, the same model was fit for samples with randomly shuffled pseudotime estimates.

Expression changes (log2(Fold-changes)) and FDR were calculated for difference between sex averaged coefficients for maternal mutants and respective *ctrls* using log-likelihood test and Benjamini-Hochberg method for multiple testing correction.

Gene Ontology enrichment analysis

Enrichment analysis for Gene Ontology terms was done using R package topGO (version 2.48.0)[80] with parameters method="weight01" and statistic="fisher" extracting Gene Ontology gene annotation from the Bioconductor Annotation Package org.Mm.eg.db (version 3.15.0)[81] (Table S7) or mapping to slim Gene Ontology using map2slim tool (Figures 7B and S2G; Table S6).

Visualization of Gene Ontology enrichments was done by calculation of pairwise Jaccard distances between significant Gene Ontology terms based on intersections and unions of significantly affected gene sets having corresponding Gene Ontology term annotations. After pairwise Jaccard distances between Gene Ontology terms were calculated we applied multidimensional scaling (MDS) using R function cmdscale and represented Gene Ontology terms on a 2D plot where size was scaled by obs./exp. ratio, color was chosen to reflect statistical significance, and relative position reflects similarities in gene sets (Figure S2G).

Alignment and quality control of WGBS datasets

The quality of the data was assessed using FastQC (v0.11.8) and adapters were trimmed using TrimGalore (v0.6.2)[82] with parameters "–illumina –stringency 5 –clip_r1 6 –three_prime_clip_r1 6". Alignment to mm10 genome and deduplication was done using Bismark (v0.22.3)[83] with parameters "–local –non_directional" (Figures S4B and S4C). Reproducibility of samples has been assessed by calculating levels of DNAme in 1e+5 randomly selected 500bp genomic tiles, calculating pairwise Euclidean distances between samples and performing multidimensional scaling for obtained distance matrix (Figure S4A). Samples with small library sizes ($\leq$ 10e+6 reads), insufficient bisulfite conversion efficiency estimated using total levels of non-CpG (CHG, CHH) methylation ($\geq$7%) as well as outlier samples were removed from the analysis and remaining samples for each genotype were merged for further analysis. Allelic analysis of WGBS datasets was performed using SNPsplit.[84]

Analysis of CUT&RUN sequencing data

Reads from the CUT&RUN experiments were aligned to a composite mouse-fly genome (GRCm38/mm10 and dm6 UCSC assemblies, https://genome.ucsc.edu) using the qAlign function of the QuasR R-bioconductor package with the "paired" parameter set to 'fr' and the remaining parameters set to defaults.

To account for coverage distortions across the different CUT&RUN libraries we employed a procedure whereby counts where normalized to the total number of reads originating from regions (gene body + 5kbp flanking windows) of genes stably expressed across genotypes according to the matching RNAseq data (absolute Log2CPM > 0.25, absolute Log2FoldChange < 0.25). This procedure assumes that chromatin marks over stably expressed regions remain -on average- unchanged. In the case of counts over

CellPress
OPEN ACCESS

genomic tiles normalized counts were also min-shifted to the 15th centile to account for detection-limit and background signal level differences across libraries.

For the presented results libraries of biological replicates of either *wt* or mutant libraries have been merged unless otherwise specified.

Genome arithmetic operations

Read counting over specified genomic intervals, or genomic tiles of the mouse genome (GRCm38/mm10) was carried out with the QuasR count function with the ‘‘orientation’’ parameter set to ‘any’ and default parameters otherwise, excluding non-canonical chromosomes.

Gene and CpG-island coordinates for overlap counting and other genome-arithmetic operations were taken from the UCSC annotation database (mm10.knownGene and mm10 CpG Island track respectively, https://genome.ucsc.edu), unless otherwise specified. Specifically, for CpG-island counting operations the regions were resized to windows with length equal to the median CpG island width (533nt) preserving their center coordinate.

Clustering and heatmaps for chromatin, sequence and expression features

Clusters presented on the different subsets of genes / intergenic regions were determined using k-means clustering with 100 random starts using the kmeans implementation of R stats on standardized features. Heatmaps of genomic tiles were plotted using the Heatmap function of the ComplexHeatmap R-Bioconductor package.[85] Plotted tiles were smoothed with a running mean smoothing kernel of width 5. Color-scales were thresholded on both low and high values at the 2nd and 98th centile respectively.

Regularized linear regression for chromatin-mark and DNA-methylation-modelling

For the modelling of basal methylation levels, differential methylation levels and differential H3K36me2 levels across genotypes we opted for the lasso regression framework to identify predictive explanatory variables. Since multicollinearity was extensive among the set of predictors, we also describe for each prediction task the covariance structure of the independent variables to facilitate model interpretability. For all models, CpG island chromatin and sequence features were calculated on the 533nt resized set of CpG islands except for the lower coverage CUT&RUN datasets (H3K36me2, H2AK119ub1) where the signal was calculated on a larger window (1066nt) to account for the lower resolution. For gene-body (gb) and RNA-seq expression features the signal refers to gene-bodies of the nearest annotated genes. All independent variables were z-score normalized prior to fitting.

For the lasso regression fitting we used the CRAN glmnet R package implementation. Briefly, in a first step we selected the lambda parameter as the largest value of lambda such that error is within 1 standard error of the minimum (lambda.1se) in a 10-fold cross validation. We next performed lasso regression with the selected lambda parameter.

Example commands

```
CV_fit <- cv.glmnet(X, Y, alpha=1, nfolds=10, lambda= 10^seq(-2, -6, by = -.05))
lambda_best <- CV_fit$lambda.1se
fit <- glmnet(x, y, lambda=lambda_best)
```

In the case of modelling differential methylation or differential H3K36me2 levels we used a setting where the response variable is the mutant genotype levels while including wild-type levels as a predictor. This choice circumvents the issue of selecting features that are predictive of wild-type levels of the dependent variable (as opposed to differential levels) which is inadvertently the case when one models directly differential levels in the absence of the wild-type levels among the set of predictors. One alternative implementation -that yields almost identical results to the ones presented here - is to first regress out the effect of wild-type levels on mutant signal levels and subsequently model the residual on the remaining set of predictors. In our implementation we omit the wild-type signal levels when we present the most important predictors.

Data visualization

Chromatin, sequence and expression feature heatmaps were generated with the ComplexHeatmap R bioconductor package. Chromatin features are Z-score transformed except when otherwise indicated. In heatmaps in Figures 2D, 3D, S3B, and S4G, we included only genes with gene bodies > 5Kbp and < 80kbp in order to reduce plotting artefacts. Calculations were performed on 10 equal width (500bp) windows for both the regions of 5kbps upstream of the TSS and 5kbps downstream of the TTS. Calculations were performed on 20 variable width windows for the full gene-body regions. Prior to plotting local smoothing of the genomic signals was performed using a local mean smoother with a kernel size of 5 windows.

Boxplots were generated with the ggplot2[86] *geom_boxplot* function. Whiskers extend to 1.5 the IQR range.

Chromatin and sequence analysis at UCSC-annotated CGIs

Enrichments for H2AK119u1 in *wt* FGO, H3K4me3 in *wt* GO as well as H3K36me2 in *ctrl* and mutant FGO were calculated in 533bp regions around the center of 16,023 CGIs. CGIs were split into groups with low (7,506 CGIs) and high (8,517 CGIs) H2AK199u1 levels based on calculated enrichments in *wt* FGO. Next, CGIs were split into 10 groups according to H3K4me3 levels in *wt* GO such that

each group contains similar number of CGIs (1,439–1,842) using function cut_number from ggplot2 R package.[86] Finally, for each H3K4me3 group separately for low and high H2AK119u1 groups we plotted boxplots for H3K36me2 in *ctrl* and mutant FGOs as well counts of CCG and CGG trinucleotides normalized per 100bp (Figure 5D).

QUANTIFICATION AND STATISTICAL ANALYSIS

Statistical analyses were performed using R. All statistical tests, p values and sample numbers were stated in figure panels or legends. Statistical p values were calculated using two-tailed Student's t-test and Tukey's HSD test in immunostaining signal intensity comparison. Fisher's exact test was used for the comparisons of embryonic development results.

Developmental Cell, Volume *60*

Supplemental information

Preventing CpG hypermethylation in oocytes safeguards mouse development

Yumiko K. Kawamura, Evgeniy A. Ozonov, Panagiotis Papasaikas, Takashi Kondo, Nhuong V. Nguyen, Michael B. Stadler, Sebastien A. Smallwood, Haruhiko Koseki, and Antoine H.F.M. Peters

Supplemental Information

Preventing CpG hypermethylation in oocytes safeguards mouse development

Yumiko K. Kawamura, Evgeniy A. Ozonov, Panagiotis Papasaikas, Takashi Kondo, Nhuong V. Nguyen, Michael B. Stadler, Sebastien A. Smallwood, Haruhiko Koseki and Antoine H.F.M Peters.

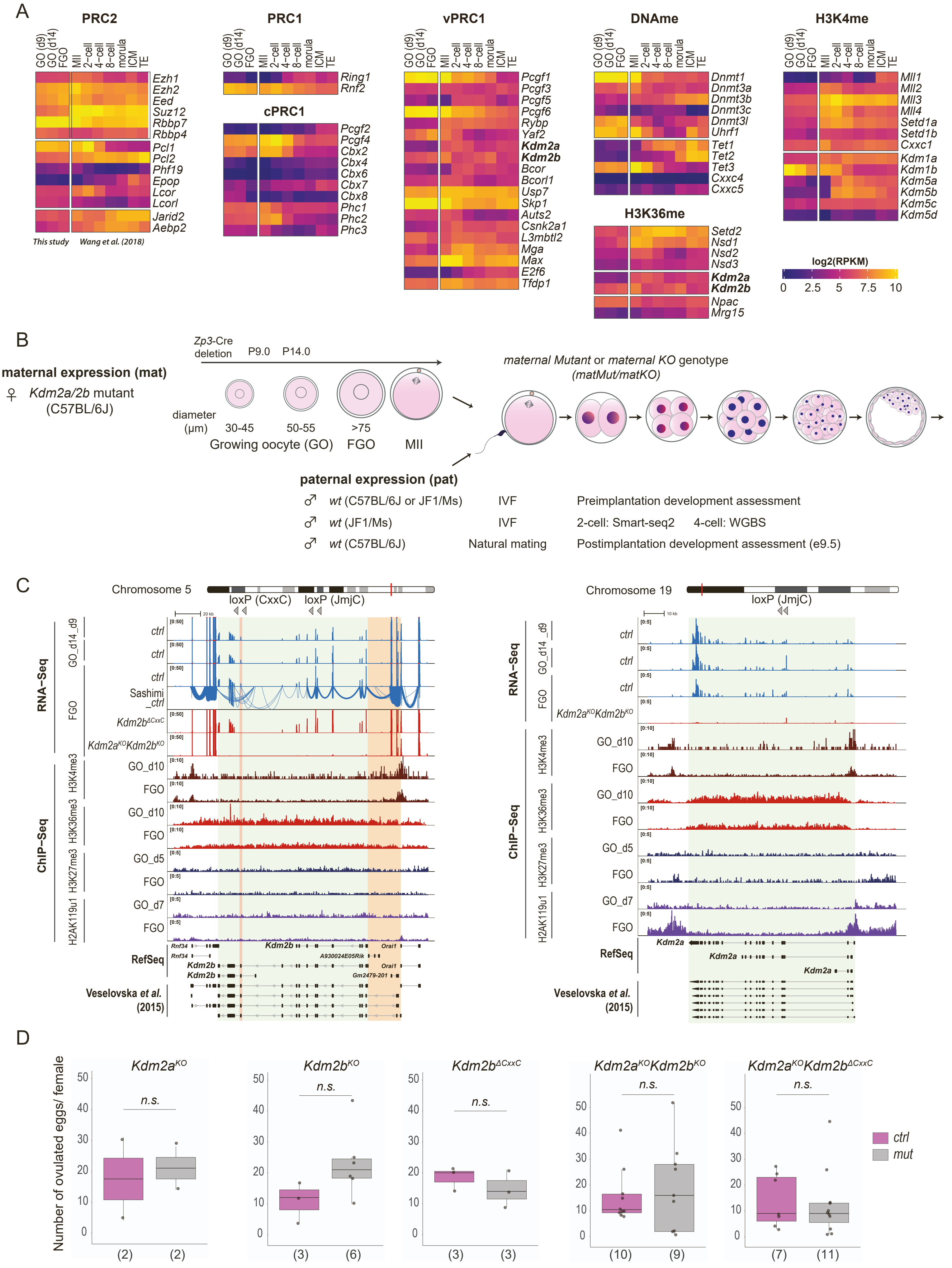

A
PRC2
PRC1
cPRC1
vPRC1
DNAme
H3K36me
H3K4me
GO (d9)
GO (d14)
FGO
MII
2-cell
4-cell
8-cell
morula
ICM
TE
Ezh1
Ezh2
Eed
Suz12
Rbbp7
Rbbp4
Pcl1
Pcl2
Phf19
Epop
Lcor
Lcorl
Jarid2
Aebp2
This study
Wang et al. (2018)
Ring1
Rnf2
Pcgf2
Pcgf4
Cbx2
Cbx4
Cbx6
Cbx7
Cbx8
Phc1
Phc2
Phc3
Pcgf1
Pcgf3
Pcgf5
Pcgf6
Rybp
Yaf2
Kdm2a
Kdm2b
Bcor
Bcorl1
Usp7
Skp1
Auts2
Csnk2a1
L3mbtl2
Mga
Max
E2f6
Tfdp1
Dnmt1
Dnmt3a
Dnmt3b
Dnmt3c
Dnmt3l
Uhrf1
Tet1
Tet2
Tet3
Cxxc4
Cxxc5
Setd2
Nsd1
Nsd2
Nsd3
Kdm2a
Kdm2b
Npac
Mrg15
Mll1
Mll2
Mll3
Mll4
Setd1a
Setd1b
Cxxc1
Kdm1a
Kdm1b
Kdm5a
Kdm5b
Kdm5c
Kdm5d
log2(RPKM)
0 2.5 5 7.5 10
B
maternal expression (mat)
♀ Kdm2a/2b mutant (C57BL/6J)
Zp3-Cre deletion
P9.0
P14.0
diameter (μm)
30-45
50-55
>75
Growing oocyte (GO)
FGO
MII
maternal Mutant or maternal KO genotype (matMut/matKO)
paternal expression (pat)
♂ wt (C57BL/6J or JF1/Ms) IVF Preimplantation development assessment
♂ wt (JF1/Ms) IVF 2-cell: Smart-seq2 4-cell: WGBS
♂ wt (C57BL/6J) Natural mating Postimplantation development assessment (e9.5)
C
Chromosome 5
loxP (CxxC)
loxP (JmjC)
RNA-Seq
ChIP-Seq
GO_d14_d9
FGO
ctrl
Sashimi_ctrl
Kdm2b^ΔCxxC
Kdm2a^KO Kdm2b^KO
H3K4me3
H3K36me3
H3K27me3
H2AK119u1
GO_d10
GO_d5
GO_d7
RefSeq
Rnf34
Kdm2b
Orai1
A930024E05Rik
Gm2479-201
Veselovska et al. (2015)
Chromosome 19
loxP (JmjC)
Kdm2a
D
Number of ovulated eggs/ female
Kdm2a^KO
Kdm2b^KO
Kdm2b^ΔCxxC
Kdm2a^KO Kdm2b^KO
Kdm2a^KO Kdm2b^ΔCxxC
n.s.
(2) (2)
(3) (6)
(3) (3)
(10) (9)
(7) (11)
ctrl
mut

Figure S1: Deficiency of *Kdm2a* and/or *Kdm2b* does not impair oogenesis, related to Figure 1.

- **A.** RNA expression of multiple genes in GOs (at day 9, day 14), FGOs and pre-implantation embryos at different stages of development [S1].
- **B.** Scheme to generate oocyte specific deficiency and maternally deficient embryos (*matKO*) by crossing conditional $Kdm2a^{KO}$, $Kdm2b^{KO}$, $Kdm2b^{\Delta CxxC}$, double or compound mutant females (on C57BL/6J genetic background) with *wt* males (JF1 or C57BL/6J genetic background) or by performing *in vitro* fertilization (IVF) followed by *in vitro* culture for several days, as indicated. Oocyte specific deletion was mediated by deletion of floxed alleles by CRE-recombinase, expressed from a *Zona pellucida 3*-cre (*Zp3*-cre) transgene initiated in primary GOs [S2]. Deletion of the JmjC domain encoding exons of $Kdm2a^{fl\text{-}JmjC}$ and $Kdm2b^{fl\text{-}JmjC}$ [S3] results in a translational frame shift, decay of mRNA transcripts and greatly reduced expression. Deletion of the CGI-binding Zinc Finger Domain "CxxC" encoding exon of $Kdm2b^{fl\text{-}CxxC}$ [S4] causes an in-frame excision leading to expression of a slightly smaller KDM2B protein unable to be recruited to CpG islands (see also Figure S1C) [S3, S4].
- **C.** (Left) RNA expression and chromatin status along the RefSeq-annotated *Kdm2b* locus (in reverse orientation; highlighted in green) in *ctrl* and various *Kdm2b* conditionally mutant oocytes. *Kdm2b* and neighboring *Rnf34* and *Orai1* genes are highly expressed throughout oogenesis and are associated with high H3K4me3 occupancy at gene promoters, widespread H3K36me3 enrichment along gene bodies and absence of repressive H3K27me3 and H2AK119u1. Chromatin and splice-junction analysis revealed that *Kdm2b* transcription in oocytes initiates from an alternative promoter, encoding a protein that is 54 amino acids longer than the canonical form [S4, S5]. The promoter also drives the expression of the short *Gm2479-201* transcript. The region encompassing the alternative transcriptional start site (TSS) as well as the exon encoding the CXXC domain of *Kdm2b* are highlighted in orange. The positions of LoxP sites in the floxed *JmjC* (referred to as *KO*) and *CxxC* conditional alleles are indicated.

  (Right) RNA expression and chromatin status along the RefSeq-annotated *Kdm2a* locus (in reverse orientation; highlighted in green) in *ctrl* and *Kdm2a* conditionally deficient oocytes. *Kdm2a* is expressed throughout oogenesis and is marked by H3K4me3 at its promoter and by H3K36me3 along its gene body. While H3K27me3 is absent, H2AK119u1 levels are increased upon oocyte growth in FGOs.
- **D.** Numbers of ovulated eggs of single and double conditionally mutant females upon hormonal superovulation treatment. Numbers of analyzed females are indicated. P-values according to two-sided student's *t*-test.

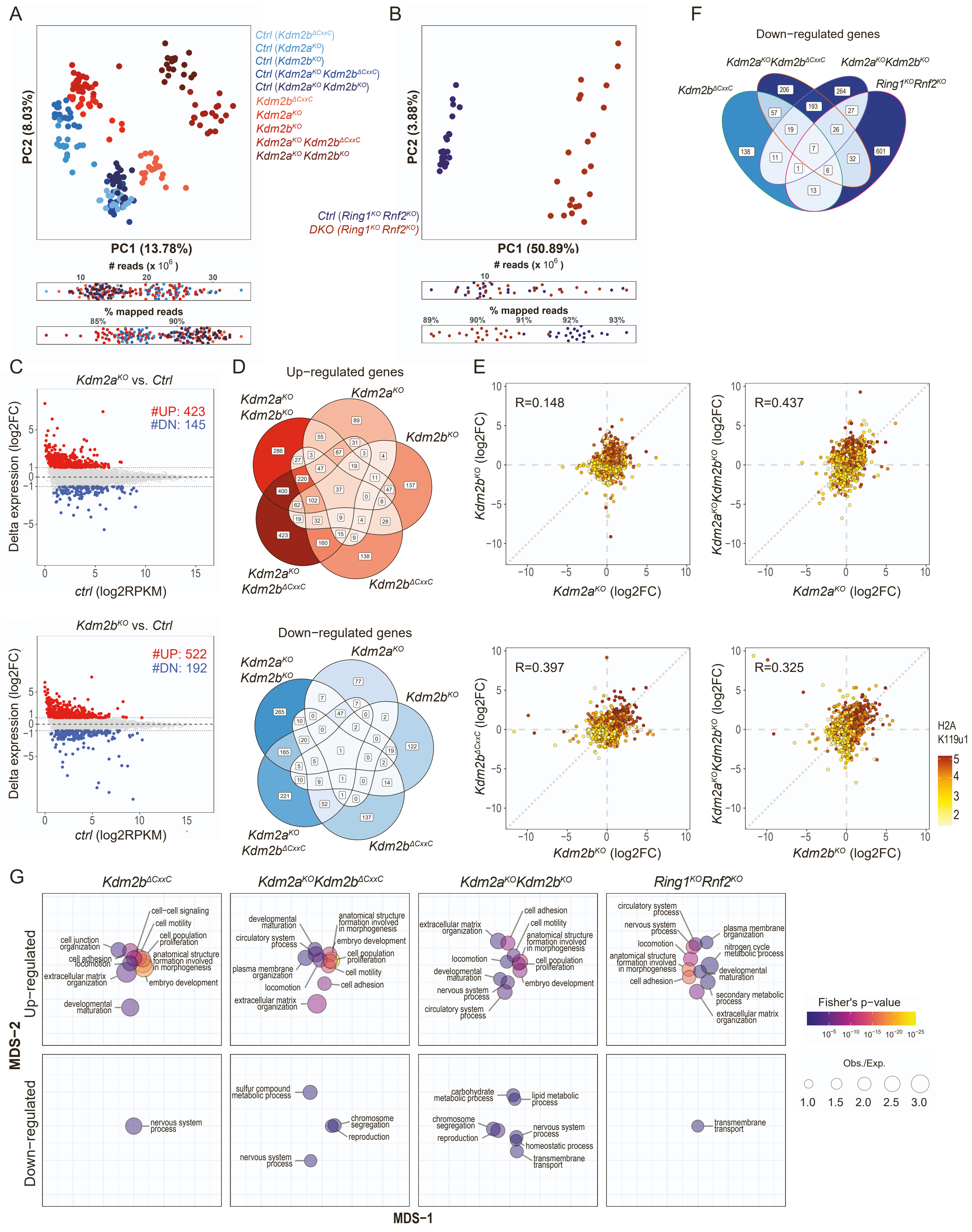

A
B
C
D
E
F
G
PC1 (13.78%)
PC2 (8.03%)
PC1 (50.89%)
PC2 (3.88%)
reads (x 10^6)
% mapped reads
Down-regulated genes
Up-regulated genes
Kdm2a^KO vs. Ctrl
Kdm2b^KO vs. Ctrl
#UP: 423
#DN: 145
#UP: 522
#DN: 192
Delta expression (log2FC)
ctrl (log2RPKM)
R=0.148
R=0.437
R=0.397
R=0.325
H2A K119u1
Fisher's p-value
Obs./Exp.
MDS-1
MDS-2
Up-regulated
Down-regulated

Figure S2: Quality control analyses of RNA-seq and CUT&RUN-seq data, related to Figure 2.

- **A.** PCA plot illustrating the variance in RNA-seq expression data between single FGOs of indicated genotypes (*$Kdm2a^{KO}$, $Kdm2b^{KO}$, $Kdm2b^{\Delta CxxC}$*, *$Kdm2a^{KO}Kdm2b^{\Delta CxxC}$*, *$Kdm2a^{KO}Kdm2b^{KO}$* and respective *ctrl* FGOs). Total and mapped RNA-seq read counts of individual FGOs are indicated in panels below.
- **B.** PCA plot illustrating the variance in RNA-seq expression data between single c*trl* and *$Ring1^{KO}Rnf2^{KO}$* FGOs. Total and mapped RNA-seq read counts of individual FGOs are indicated in panels below.
- **C.** MA-plots showing differential expression of *$Kdm2a^{KO}$* or *$Kdm2b^{KO}$* FGOs over respective *ctrl* FGOs (log2 fold change (log2FC)) as a function of expression in respective *ctrl* FGOs (log2RPKM). #UP and #DN refer to numbers of genes more highly or lowly expressed in mutant versus *ctrl* FGOs (log2FC > 1.0; adj P-value < 0.05).
- **D.** Venn diagrams showing numbers of genes up- or down-regulated in *$Kdm2a^{KO}$, $Kdm2b^{KO}$, $Kdm2b^{\Delta CxxC}$*, *$Kdm2a^{KO}Kdm2b^{\Delta CxxC}$* and/or *$Kdm2a^{KO}Kdm2b^{KO}$* FGOs.
- **E.** Scatter plots showing log2FC in expression of indicated mutant FGOs over respective *ctrl* FGOs versus indicated mutant FGOs over respective *ctrl* FGOs. H2AK119u1 occupancy (log2) at promoters (-1500/+500 bps of TSS) is indicated by color scale[37]. R indicates Pearson's correlation coefficient.
- **F.** Venn diagram showing numbers of genes down-regulated in *$Kdm2b^{\Delta CxxC}$*, *$Kdm2a^{KO}Kdm2b^{\Delta CxxC}$*, *$Kdm2a^{KO}Kdm2b^{KO}$* and/or *$Ring1^{KO}Rnf2^{KO}$* FGOs.
- **G.** MDS plots showing enrichments of top Gene Ontology terms for genes up- or down-regulated in *$Kdm2a^{KO}Kdm2b^{KO}$*, *$Kdm2a^{KO}Kdm2b^{\Delta CxxC}$*, *$Kdm2b^{\Delta CxxC}$* and *$Ring1^{KO}Rnf2^{KO}$* FGOs over *ctrl* FGOs. Bubbles representing GO terms are scaled according to enrichments, colored according to statistical significance and positioned relative to one another to reflect similarities between significantly affected genes with corresponding GO terms.

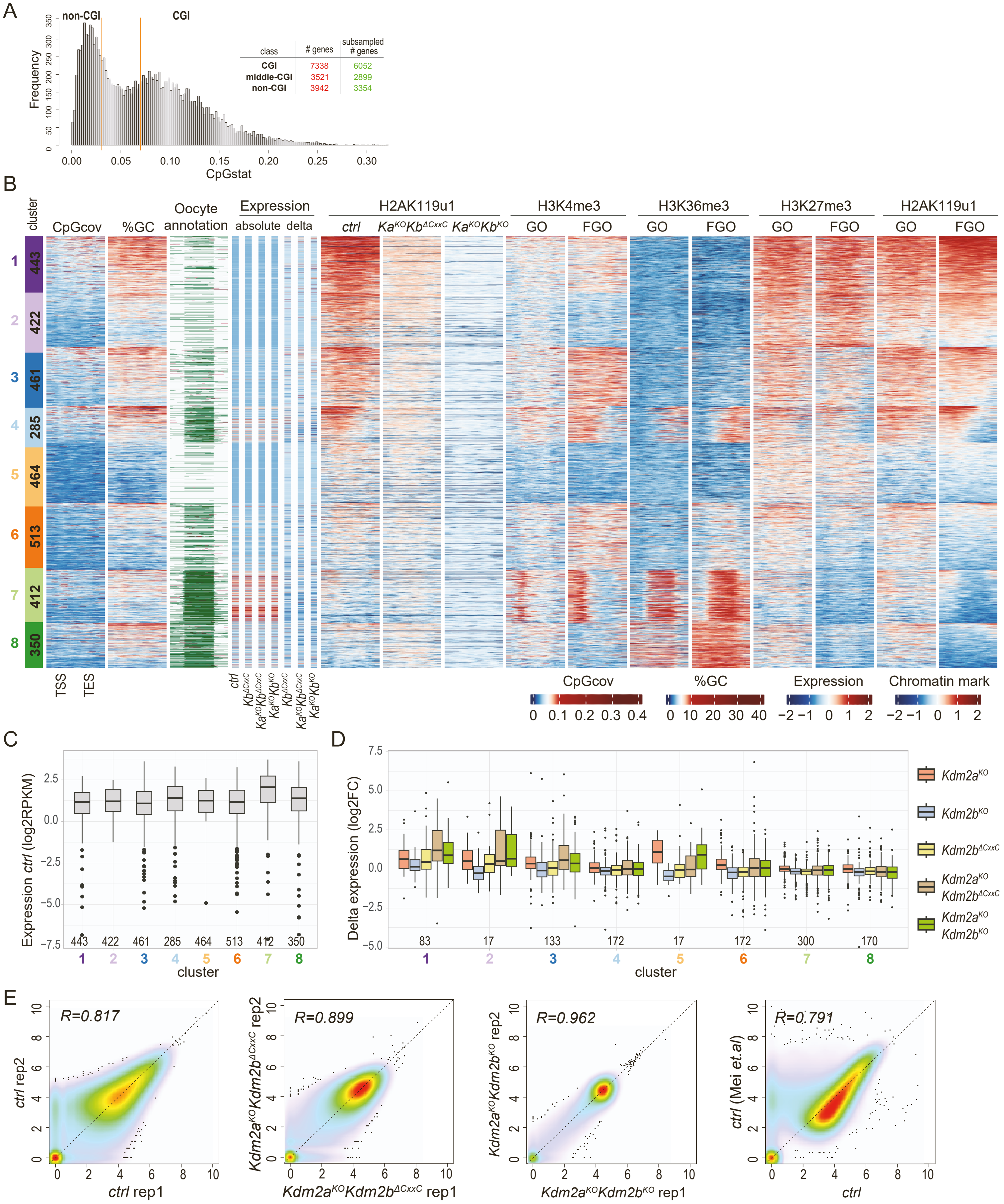

A
non-CGI
CGI
Frequency
CpGstat
class
genes
subsampled # genes
CGI 7338 6052
middle-CGI 3521 2899
non-CGI 3942 3354
B
cluster
CpGcov
%GC
Oocyte annotation
Expression
absolute delta
H2AK119u1
ctrl
H3K4me3
GO
FGO
H3K36me3
H3K27me3
TSS
TES
Expression
Chromatin mark
C
Expression ctrl (log2RPKM)
cluster
D
Delta expression (log2FC)
cluster
Kdm2a^KO
Kdm2b^KO
Kdm2b^ΔCxxC
E
R=0.817
ctrl rep2
ctrl rep1
R=0.899
R=0.962
R=0.791
ctrl (Mei et.al)
ctrl

Figure S3: KDM2A/KDM2B regulate H2AK119u1 deposition and gene expression during oogenesis, related to Figure 2.

- **A.** Distribution of genes according to the CpG density within the 500 bp region upstream of their transcriptional start site defined by UCSC. Genes with high and low CpG density are referred to as CGI- and nonCGI-promoter genes and have been further sub-selected for those lacking antisense expression during oogenesis. Sub-selected genes have been analyzed in subsequent figures.
- **B.** Heatmap displaying sequence composition, transcriptional and chromatin variables within nonCGI promoter genes (5kb upstream, TSS, gene body, TES, and 5 kb downstream) grouped into 8 gene clusters by k-means clustering. From left to right: gene numbers per cluster, CpG coverage; GC percentage; oocyte specific sense (green) and antisense (red) transcripts [S5]; absolute RNA (scaled RPKM) in *ctrl*, $Kdm2b^{\Delta CxxC}$, $Kdm2a^{KO}Kdm2b^{\Delta CxxC}$ and $Kdm2a^{KO}Kdm2b^{KO}$ FGOs; log2FC expression in mutant vs *ctrl* FGOs (delta); H2AK119u1 occupancy in FGOs of indicated genotypes; H3K4me3, H3K36me3, H3K27me3 and H2AK119u1 occupancies in *wt* GOs and FGOs [S6-S8]. All chromatin data are shown as Z-scores. Expression correlates with H3K4me3 promoter occupancy and H3K36me3 gene body occupancy while repression with broad H3K27me3 and H2AK119u1 occupancy in GOs.
- **C.** Boxplot presenting RNA expression levels of nonCGI promoter genes (in log2RPKM) per gene cluster in *ctrl* FGOs.
- **D.** Boxplot presenting log2FC in expression of nonCGI promoter genes measured in various mutant FGOs relative to respective *ctrl* FGOs, indicated per gene cluster.
- **E.** Reproducibility between replicates of H2AK119u1 CUT&RUN data of *ctrl*, $Kdm2a^{KO}Kdm2b^{\Delta CxxC}$ and $Kdm2a^{KO}Kdm2b^{KO}$ FGOs produced in this study. Likewise, between CUT&RUN data in *ctrl* FGOs of this study (replicates pooled) and publicly available data [S8]. Depicted are log2 transformed, library normalized counts over all 5kbp genomic tiles.

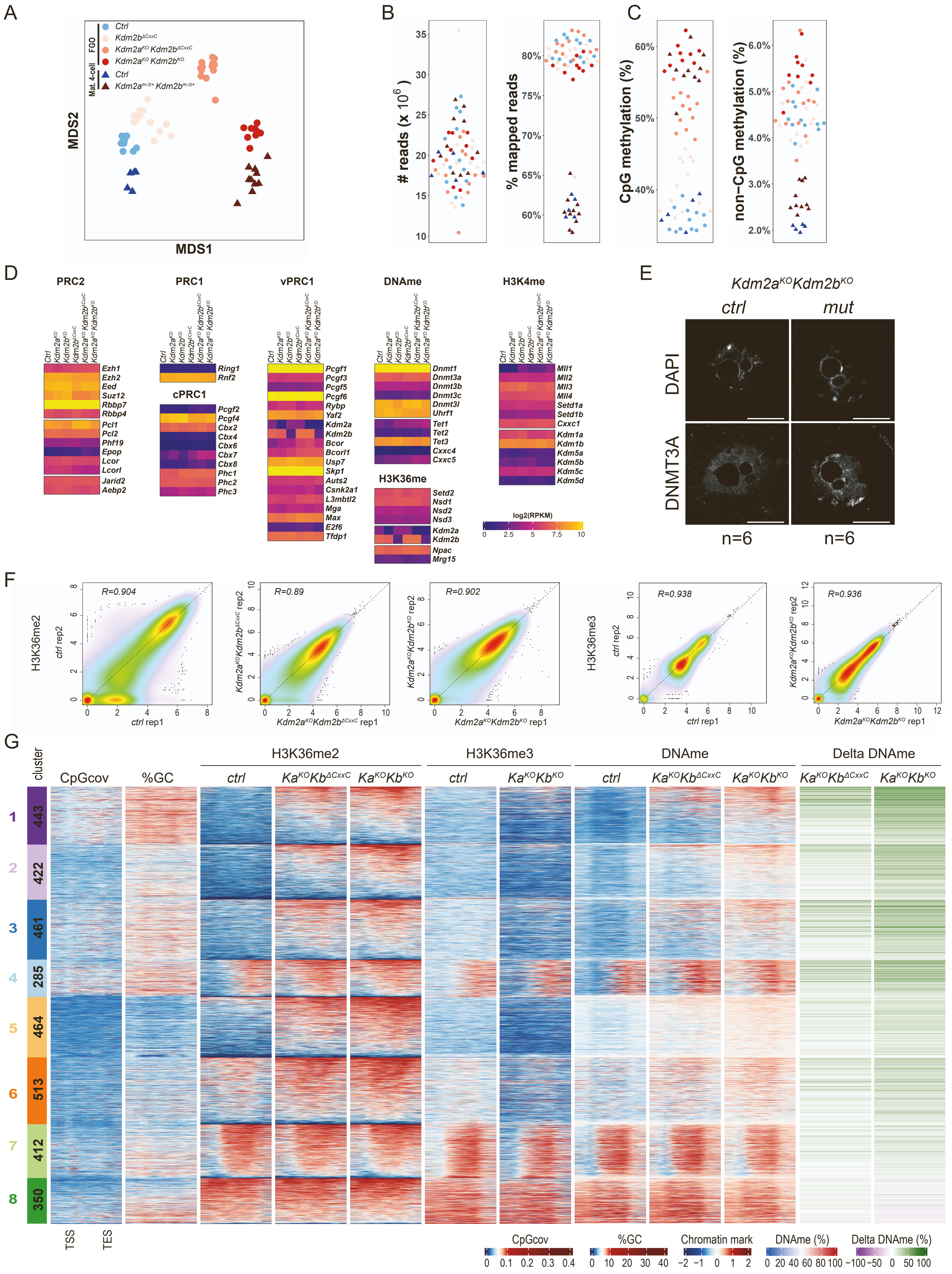

A
B
C
D
E
F
G
Ctrl
Kdm2bΔCxxC
Kdm2aKO Kdm2bΔCxxC
Kdm2aKO Kdm2bKO
Ctrl
Kdm2am-/z+ Kdm2bm-/z+
FGO
Mat. 4-cell
MDS1
MDS2
reads (x 10^6)
% mapped reads
CpG methylation (%)
non-CpG methylation (%)
PRC2
PRC1
cPRC1
vPRC1
DNAme
H3K36me
H3K4me
log2(RPKM)
Kdm2aKOKdm2bKO
ctrl
mut
DAPI
DNMT3A
n=6
n=6
H3K36me2
H3K36me3
R=0.904
R=0.89
R=0.902
R=0.938
R=0.936
ctrl rep1
ctrl rep2
Kdm2aKOKdm2bΔCxxC rep1
Kdm2aKOKdm2bKO rep1
cluster
CpGcov
%GC
H3K36me2
H3K36me3
DNAme
Delta DNAme
KaKOKbΔCxxC
KaKOKbKO
TSS
TES
Chromatin mark
DNAme (%)
Delta DNAme (%)

Figure S4: Quality control analyses of WGBS and CUT&RUN-seq data, related to Figure 3.

A. MDS plot illustrating variance in WGBS data between multiple libraries generated for indicated genotypes (*Kdm2b*$^{\Delta CxxC}$, *Kdm2a*^{KO}*Kdm2b*$^{\Delta CxxC}$, *Kdm2a*^{KO}*Kdm2b*^{KO} and *ctrl* FGOs).

B. Total and mapped WGBS-seq read counts of individual libraries indicated in panel S4A.

C. Percentage CpG and non-CpG (CHG, CHH) methylation of individual libraries indicated in panel S4A.

D. RNA expression of multiple genes in *ctrl* and *Kdm2a*^{KO}*, Kdm2b*^{KO}*, Kdm2b*$^{\Delta CxxC}$, *Kdm2a*^{KO}*Kdm2b*$^{\Delta CxxC}$, *Kdm2a*^{KO}*Kdm2b*^{KO} mutant FGOs. All RNA expression data is based on polyA-primed RNA capture and Smart-seq2 library generation.

E. Representative immunofluorescence images of DNMT3A localization in *ctrl* and *Kdm2a*^{KO}*Kdm2b*^{KO} FGOs. Numbers of analyzed oocytes are indicated. Scale bars, 10 µm.

F. Reproducibility between replicates of H3K36me2 and H3K36me3 CUT&RUN data of *ctrl, Kdm2a*^{KO}*Kdm2b*$^{\Delta CxxC}$ and *Kdm2a*^{KO}*Kdm2b*^{KO} FGOs produced in this study. Depicted are log2 transformed, library normalized counts over all 5kbp genomic tiles.

G. Heatmap displaying sequence composition and chromatin variables within 8 nonCGI promoter gene clusters in FGOs, as described in Figure S3B. From left to right: gene numbers per cluster, CpG coverage; GC percentage; H3K36me2, H3K36me3 and DNAme in FGOs of indicated genotypes; differential (Delta) DNAme at nonCGI-promoters in *Kdm2a*^{KO}*Kdm2b*^{KO} and *Kdm2a*^{KO}*Kdm2b*$^{\Delta CxxC}$ FGOs.

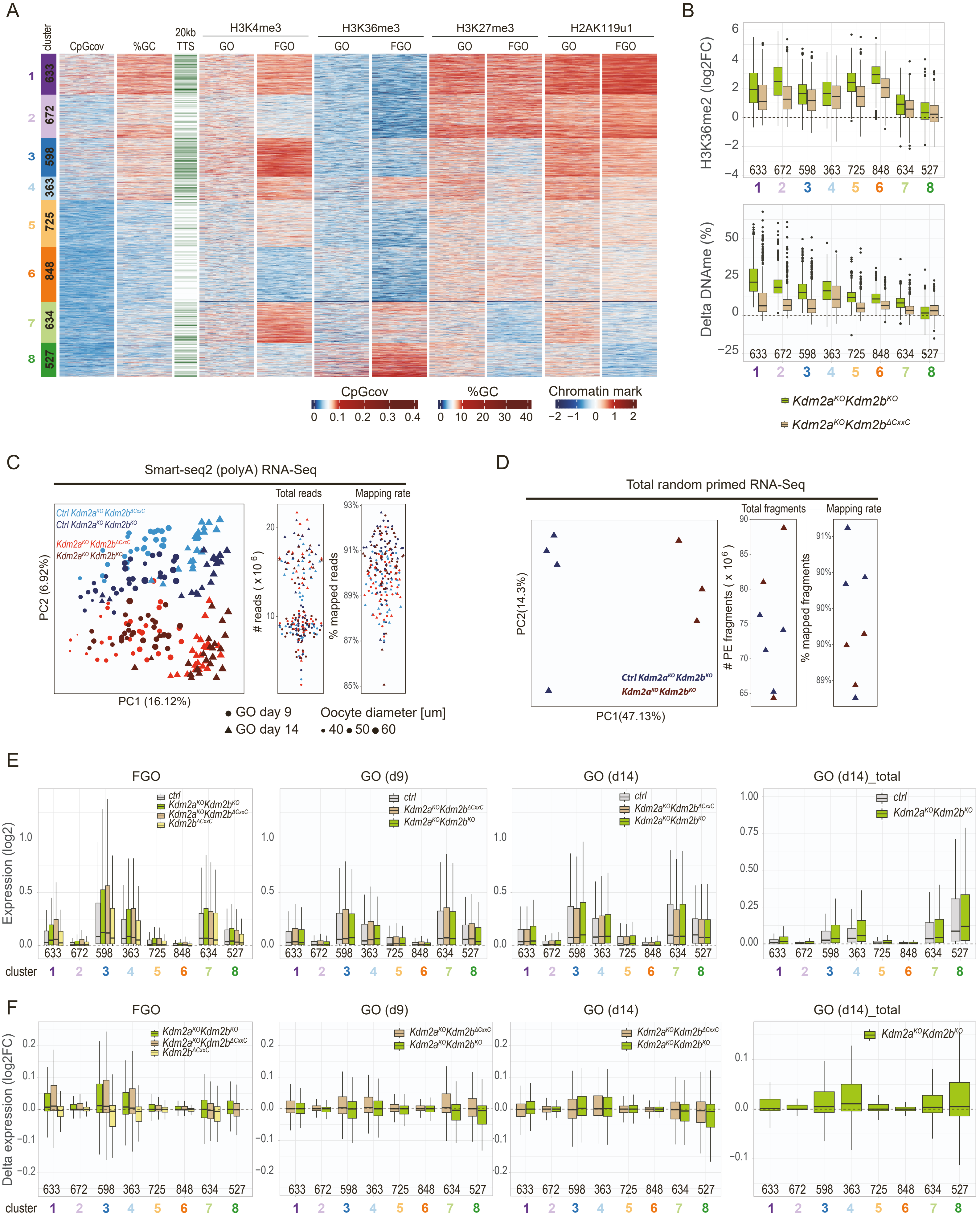
A
cluster
CpGcov
%GC
20kb TTS
H3K4me3
H3K36me3
H3K27me3
H2AK119u1
GO
FGO
1 633
2 672
3 598
4 363
5 725
6 848
7 634
8 527
CpGcov
0 0.1 0.2 0.3 0.4
%GC
0 10 20 30 40
Chromatin mark
−2 −1 0 1 2
B
H3K36me2 (log2FC)
Delta DNAme (%)
633 672 598 363 725 848 634 527
1 2 3 4 5 6 7 8
Kdm2aKOKdm2bKO
Kdm2aKOKdm2bΔCxxC
C
Smart-seq2 (polyA) RNA-Seq
Ctrl Kdm2aKO Kdm2bΔCxxC
Ctrl Kdm2aKO Kdm2bKO
Kdm2aKO Kdm2bΔCxxC
Kdm2aKO Kdm2bKO
PC2 (6.92%)
PC1 (16.12%)
Total reads
reads (x10 6)
Mapping rate
% mapped reads
GO day 9
GO day 14
Oocyte diameter [um]
40 50 60
D
Total random primed RNA-Seq
PC2(14.3%)
PC1(47.13%)
Ctrl Kdm2aKO Kdm2bKO
Kdm2aKO Kdm2bKO
Total fragments
PE fragments (x 10 6)
Mapping rate
% mapped fragments
E
FGO
GO (d9)
GO (d14)
GO (d14)_total
Expression (log2)
ctrl
Kdm2aKOKdm2bKO
Kdm2aKOKdm2bΔCxxC
Kdm2bΔCxxC
cluster
F
Delta expression (log2FC)

Figure S5: H3K36me2 and DNAme accumulate throughout the genome of *Kdm2a*/*Kdm2b* mutant oocytes, independently of transcription, related to Figure 4.

- **A.** Heatmap displaying sequence composition and chromatin variables within 8 clusters of 10 kb intergenic regions (20 neighboring 500 bp bins) in oocytes. From left to right: number of regions per cluster, CpG coverage; GC percentage; presence of annotated TTS in 20 kb flanking regions, that could be compatible with run-through transcription through the window; H3K4me3, H3K36me3, H3K27me3 and H2AK119u1 occupancy in *wt* GOs and FGOs [S6-S9].
- **B.** Boxplots displaying differential H3K36me2 and DNAme for 10 kb intergenic regions in 8 clusters in $Kdm2a^{KO}Kdm2b^{KO}$ or $Kdm2a^{KO}Kdm2b^{\Delta CxxC}$ FGOs relative to *ctrl* FGOs, as indicated. Numbers of regions per cluster are indicated.
- **C.** PCA plot illustrating variance in smart-seq2 polyA-based RNA-seq expression data between single GOs isolated at day 9 and 14 of indicated genotypes ($Kdm2a^{KO}Kdm2b^{\Delta CxxC}$, $Kdm2a^{KO}Kdm2b^{KO}$ and respective *ctrl* FGOs). Oocyte diameter, total and mapped RNA-seq read counts of individual GOs are also indicated.
- **D.** PCA plot illustrating variance in total random-primed RNA-seq expression data between libraries prepared of pooled $Kdm2a^{KO}Kdm2b^{KO}$ and *ctrl* GOs isolated at day 14 of development. Total and mapped RNA-seq fragment counts per library are indicated as well.
- **E.** Boxplots displaying absolute expression for 10 kb intergenic regions in 8 clusters in *ctrl* and $Kdm2b^{\Delta CxxC}$, $Kdm2a^{KO}Kdm2b^{\Delta CxxC}$ and $Kdm2a^{KO}Kdm2b^{KO}$ mutant GOs isolated at day9 and day14, and in FGOs. Numbers of regions per cluster are indicated. RNA expression data are based on polyA-primed RNA capture and Smart-seq2 library generation of single oocytes or on random primed (total) RNA capture of pools of day14 GOs as indicated.
- **F.** Boxplots displaying log2FC expression for 10 kb intergenic regions in 8 clusters in $Kdm2b^{\Delta CxxC}$, $Kdm2a^{KO}Kdm2b^{\Delta CxxC}$ and $Kdm2a^{KO}Kdm2b^{KO}$ samples relative to *ctrl* samples as shown in panel S5E.

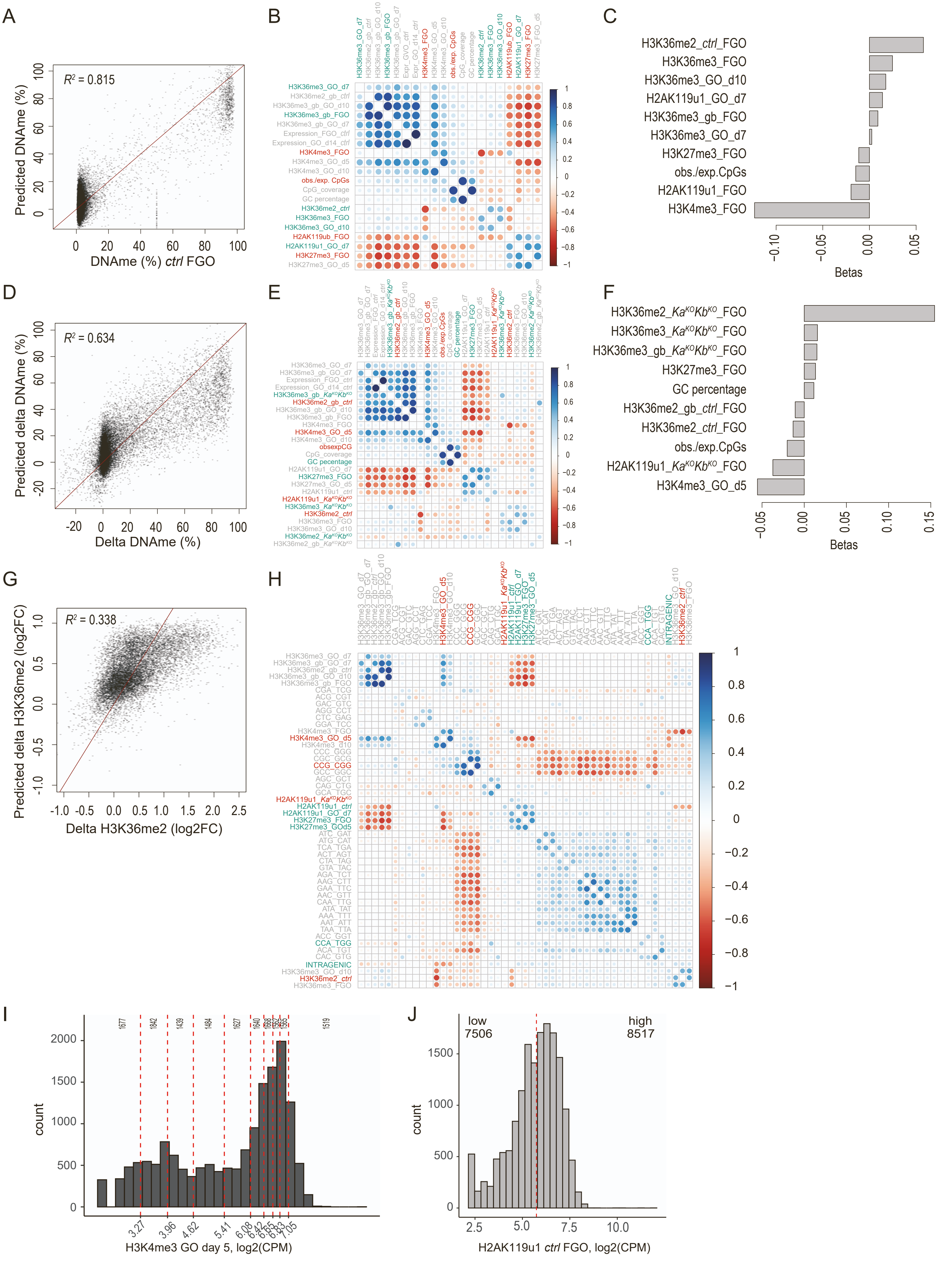

A
B
C
D
E
F
G
H
I
J
R^2 = 0.815
Predicted DNAme (%)
DNAme (%) ctrl FGO
H3K36me2_ctrl_FGO
H3K36me3_FGO
H3K36me3_GO_d10
H2AK119u1_GO_d7
H3K36me3_gb_FGO
H3K36me3_GO_d7
H3K27me3_FGO
obs./exp.CpGs
H2AK119u1_FGO
H3K4me3_FGO
Betas
R^2 = 0.634
Predicted delta DNAme (%)
Delta DNAme (%)
H3K36me2_$Ka^{KO}Kb^{KO}$_FGO
H3K36me3_$Ka^{KO}Kb^{KO}$_FGO
H3K36me3_gb_$Ka^{KO}Kb^{KO}$_FGO
H3K27me3_FGO
GC percentage
H3K36me2_gb_ctrl_FGO
H3K36me2_ctrl_FGO
obs./exp.CpGs
H2AK119u1_$Ka^{KO}Kb^{KO}$_FGO
H3K4me3_GO_d5
Betas
R^2 = 0.338
Predicted delta H3K36me2 (log2FC)
Delta H3K36me2 (log2FC)
count
H3K4me3 GO day 5, log2(CPM)
low
7506
high
8517
count
H2AK119u1 ctrl FGO, log2(CPM)

Figure S6: Identifying chromatin and sequence features underlying aberrant H3K36me2 and DNAme acquisition in *Kdm2a*/*Kdm2b* mutant oocytes by regularized linear regression analysis, related to Figure 5.

A. Scatter plot showing DNAme (%) at promoter and intragenic CGIs measured in *ctrl* FGOs versus DNAme (%) at CGIs as predicted as predicted by regularized linear regression analysis using chromatin states in *ctrl* oocytes. R^2 = 0.815. Line represents diagonal.

B. Dotplot diagram presenting the correlations between sequence, expression and chromatin features at CGIs measured in GOs and FGOs as indicated by the blue to red color gradient. Green and red labeled parameters contribute positively or negatively to the DNAme predictions as described in Figure S6A. Abbreviations: gb = signal in gene body; obs./exp. CpGs = observed over expected CpGs in CGIs; d7, d10: postnatal day 7 or 10.

C. Barplot showing the *beta* coefficients of the top 10 variables contributing to the prediction of DNAme status at CGIs in *ctrl* FGOs as described in Figure S6A.

D. Scatter plot showing the difference in DNAme (%) at promoter and intragenic CGIs in $Kdm2a^{KO}Kdm2b^{KO}$ over *ctrl* FGOs versus the difference in DNAme (%) between genotypes as predicted by regularized linear regression analysis using chromatin states in *ctrl* and $Kdm2a^{KO}Kdm2b^{KO}$ oocytes. R^2 = 0.634. Line represents diagonal.

E. Dotplot diagram presenting the correlations between sequence, expression and chromatin features at CGIs measured in GOs and FGOs as indicated by the blue to red color gradient. Green and red labeled parameters contribute positively or negatively to the DNAme predictions as described in Figure S6D. Abbreviations: gb = signal in gene body; obs./expt. CpGs = observed over expected CpGs in CGIs; d5: postnatal day 5.

F. Barplot showing the *beta* coefficients of the top 10 variables contributing to the prediction of differential DNAme at CGIs in $Kdm2a^{KO}Kdm2b^{KO}$ over *ctrl* FGOs as described in Figure S6D.

G. Scatter plot showing the difference in H3K36me2 occupancy at promoter and intragenic CGIs in $Kdm2a^{KO}Kdm2b^{KO}$ FGOs over *ctrl* FGOs versus the difference in H3K36me2 occupancy between genotypes as predicted by regularized linear regression analysis using chromatin states in *ctrl* and $Kdm2a^{KO}Kdm2b^{KO}$ oocytes and trinucleotide sequences. R^2 = 0.338. Line represents diagonal.

H. Dotplot diagram presenting the correlations between sequence, expression and chromatin features at CGIs measured in growing and FGOs as indicated by the blue to red color gradient. Green and red labeled parameters contribute positively or negatively to the H3K36me2 predictions as described in Figure S6G. Abbreviations: gb = signal in gene body; obs./expt. CpGs = observed over expected CpGs in CGIs; Trinucleotide frequencies variables are encoded as FFF_RRR pairs where RRR is the reverse complement of FFF.

I. Histogram displaying distribution of H3K4me3 occupancy levels at 533 bp-regions surrounding 16'023 CGIs in GOs. Classification of CGIs into 10 bins with approximately equal numbers of CGIs is indicated.

J. Histogram displaying distribution of H2AK119u1 occupancy levels at 533 bp-regions surrounding 16'023 CGIs in GOs. CGIs classified as having low or high H2K119u1 levels are indicated.

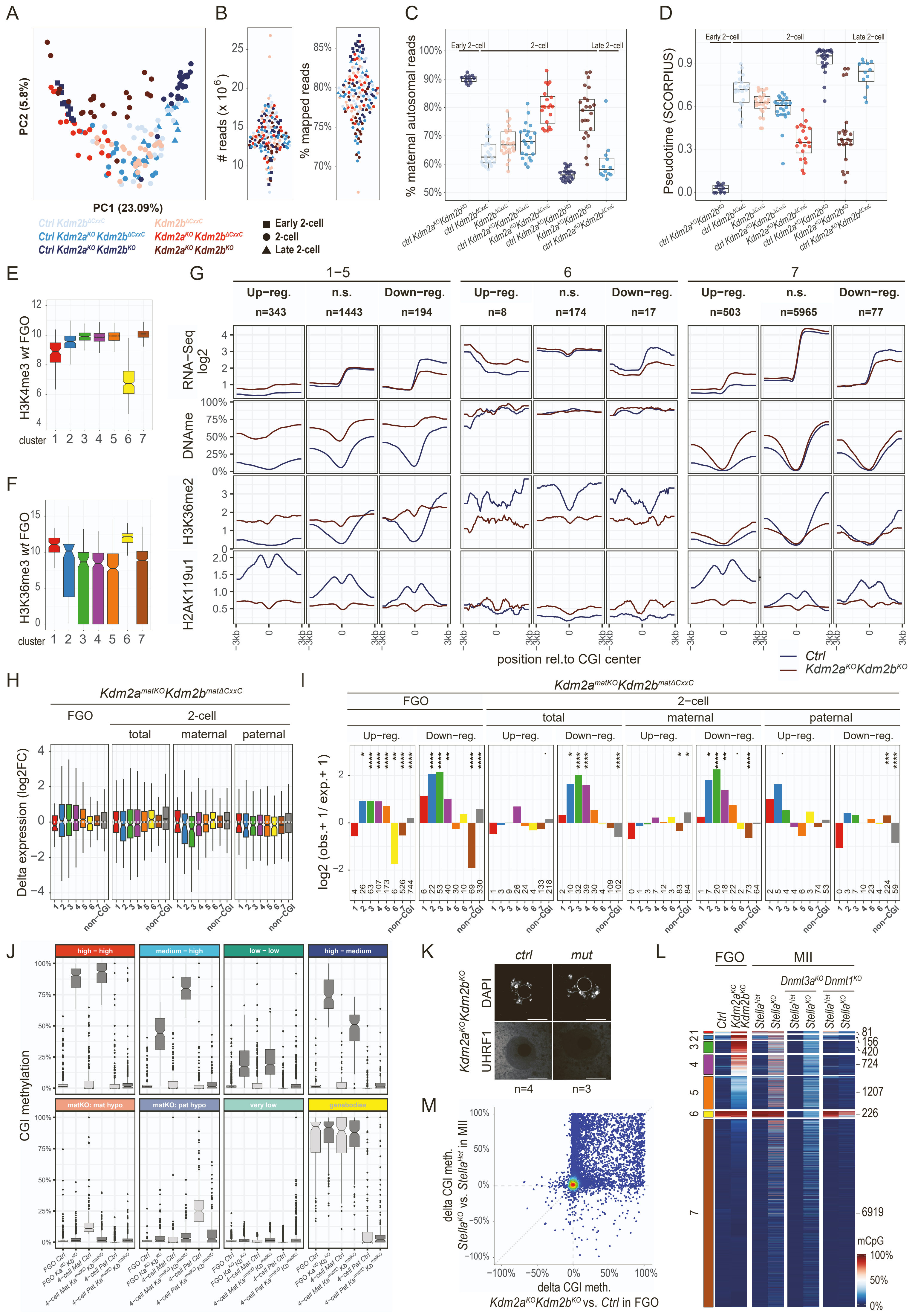
A
PC2 (5.8%)
PC1 (23.09%)
Ctrl Kdm2bΔCxxC
Kdm2bΔCxxC
Ctrl Kdm2aKO Kdm2bΔCxxC
Kdm2aKO Kdm2bΔCxxC
Ctrl Kdm2aKO Kdm2bKO
Kdm2aKO Kdm2bKO
Early 2-cell
2-cell
Late 2-cell
B
reads (x 10^6)
% mapped reads
C
% maternal autosomal reads
Early 2-cell
2-cell
Late 2-cell
D
Pseudotime (SCORPIUS)
E
H3K4me3 wt FGO
cluster
F
H3K36me3 wt FGO
cluster
G
1–5
6
7
Up-reg.
n.s.
Down-reg.
n=343
n=1443
n=194
n=8
n=174
n=17
n=503
n=5965
n=77
RNA-Seq log2
DNAme
H3K36me2
H2AK119u1
position rel.to CGI center
Ctrl
Kdm2aKOKdm2bKO
H
Kdm2amatKOKdm2bmatΔCxxC
FGO
2-cell
total
maternal
paternal
Delta expression (log2FC)
non-CGI
I
Kdm2amatKOKdm2bmatΔCxxC
FGO
2-cell
total
maternal
paternal
Up-reg.
Down-reg.
log2 (obs.+ 1/ exp.+ 1)
non-CGI
J
high – high
medium – high
low – low
high – medium
matKO: mat hypo
matKO: pat hypo
very low
genebodies
CGI methylation
FGO Ctrl
FGO KaKO KbKO
4-cell Mat Ctrl
4-cell Mat KamatKO KbmatKO
4-cell Pat Ctrl
4-cell Pat KamatKO KbmatKO
K
ctrl
mut
Kdm2aKOKdm2bKO
DAPI
UHRF1
n=4
n=3
L
FGO
MII
Dnmt3aKO Dnmt1KO
Ctrl
Kdm2aKO Kdm2bKO
StellaHet
StellaKO
StellaHet
StellaKO
StellaHet
StellaKO
81
156
420
724
1207
226
6919
mCpG
100%
50%
0%
M
delta CGI meth. StellaKO vs. StellaHet in MII
delta CGI meth. Kdm2aKOKdm2bKO vs. Ctrl in FGO

Figure S7: RNA and chromatin dynamics in *Kdm2a*/*Kdm2b* mutant versus *ctrl* FGOs at UCSC-defined CGIs associated with genes being up- or down-regulated in 2-cell embryos, related to Figure 7.

A. PCA plot illustrating variance in RNA-seq expression data between single 2-cell embryos of indicated genotypes (*Kdm2b*$^{\Delta CxxC}$, *Kdm2a*^{KO}*Kdm2b*$^{\Delta CxxC}$, *Kdm2a*^{KO}*Kdm2b*^{KO}, and respective *ctrl*) and stage (early 2-cell, 2-cell and late 2-cell stage).

B. RNA-seq read counts and mapping rates for individual 2-cell embryos in Figure S7A.

C. Percentage of SNP based allelic reads attributed as transcribed from the maternal genome for single embryos grouped by stage and genotype.

D. Inferred pseudotime for single embryos grouped by stage and genotype.

E. **F.** Boxplots displaying log2 enrichment for H3K4me3 [S7] (**E**) and H3K36me3 [S6] (**F**) in *wt* FGOs at promoter CGIs according to UCSC for genes belonging to the different DNAme clusters, as defined in Figure 7A.

G. Metaprofiles for RNA-seq read coverage (including coverage from exon-exon junctions in spliced reads), mCpG methylation, enrichments for H3K36me2 and H2AK119ub1 in *Ctrl* and *Kdm2a*^{KO}*Kdm2b*^{KO} FGOs around promoter CGIs belonging to clusters 1-5, 6 and 7 in Figure 7A nearby up-regulated, not significantly changed (n.s.) and down-regulated genes in *Kdm2a*^{KO}*Kdm2b*^{KO} FGOs. Direction from negative to positive positions around CGI centers coincide with direction from promoters to gene bodies of nearby genes.

H. Boxplot showing log2FC expression of different clusters of CGI-promoter and all nonCGI promoter genes in *Kdm2a*^{KO}*Kdm2b*$^{\Delta CxxC}$ over *ctrl* FGOs and in *Kdm2a*^{matKO}*Kdm2b*$^{mat\Delta CxxC}$ over *ctrl* 2-cell embryos according to all, mat and pat specific sequencing reads.

I. Barplot showing over-/under-representation and statistical significance of CGI-promoter genes belonging to the different DNAme clusters and being either significantly up- or down-regulated in *Kdm2a*^{KO}*Kdm2b*$^{\Delta CxxC}$ relative to *ctrl* FGOs or in *Kdm2a*^{matKO}*Kdm2b*$^{mat\Delta CxxC}$ relative to *ctrl* 2-cell embryos for all, mat and pat specific sequencing reads. Numbers of affected genes are indicated below the bars. Statistical significance is coded as follows: **** ≤ 0.001%; *** ≤ 0.01%; ** ≤ 0.1%; * ≤ 1%; · ≤ 5%.

J. Box plots showing DNAme levels at CGI-promoters, classified in 8 groups as shown in Figure 7F, in *ctrl* and *Kdm2a*^{KO}*Kdm2b*^{KO} FGOs, and in *ctrl* and *Kdm2a*^{matKO}*Kdm2b*^{matKO} 4-cell embryos for maternal and paternal genomes.

K. Representative immunofluorescence images of UHRF1 localization in *ctrl* and *Kdm2a*^{KO}*Kdm2b*^{KO} FGOs. Numbers of analyzed oocytes are indicated. Scale bars, 10 µm.

L. Heatmap showing absolute DNAme levels at CGI-gene promoters in *ctrl* and *Kdm2a*^{KO}*Kdm2b*^{KO} FGOs that had been clustered as described in panel 7A. DNAme levels at corresponding CGIs in MII-oocytes being heterozygous or deficient for *Stella* and wt or deficient for *Dnmt3a* or *Dnmt1*, as measured by RRBS, are shown as well [S10].

M. Scatter plot showing differences in DNAme at CGI-promoters in *Kdm2a*^{KO}*Kdm2b*^{KO} versus *ctrl* FGOs over *Stella*^{KO} versus *Stella*^{Het} MII oocytes.

Supplemental references

[S1]. Wang, C., Liu, X., Gao, Y., Yang, L., Li, C., Liu, W., Chen, C., Kou, X., Zhao, Y., Chen, J., et al. (2018). Reprogramming of H3K9me3-dependent heterochromatin during mammalian embryo development. Nature Cell Biology *20*. 10.1038/s41556-018-0093-4.

[S2]. De Vries, W.N., Binns, L.T., Fancher, K.S., Dean, J., Moore, R., Kemler, R., and Knowles, B.B. (2000). Expression of Cre recombinase in mouse oocytes: A means to study maternal effect genes. Genesis *26*. 10.1002/(SICI)1526-968X(200002)26:2<110::AID-GENE2>3.0.CO;2-8.

[S3]. Turberfield, A.H., Kondo, T., Nakayama, M., Koseki, Y., King, H.W., Koseki, sH., and Klose, R.J. (2019). KDM2 proteins constrain transcription from CpG island gene promoters independently of their histone demethylase activity. Nucleic Acids Research *47*. 10.1093/NAR/GKZ607.

[S4]. Blackledge, N.P., Farcas, A.M., Kondo, T., King, H.W., McGouran, J.F., Hanssen, L.L.P., Ito, S., Cooper, S., Kondo, K., Koseki, Y., et al. (2014). Variant PRC1 complex-dependent H2A ubiquitylation drives PRC2 recruitment and polycomb domain formation. Cell *157*. 10.1016/j.cell.2014.05.004.

[S5]. Veselovska, L., Smallwood, S.A., Saadeh, H., Stewart, K.R., Krueger, F., Maupetit-Méhouas, S., Arnaud, P., Tomizawa, S.i., Andrews, S., and Kelsey, G. (2015). Erratum to: Deep sequencing and de novo assembly of the mouse occyte transcriptome define the contribution of transcription to the DNA methylation landscape[Genome Biol., 16, (2015), 209]. Genome Biology. 10.1186/s13059-015-0809-8.

[S6]. Xu, Q., Xiang, Y., Wang, Q., Wang, L., Brind'Amour, J., Bogutz, A.B., Zhang, Y., Zhang, B., Yu, G., Xia, W., et al. (2019). SETD2 regulates the maternal epigenome, genomic imprinting and embryonic development. Nature Genetics *51*. 10.1038/s41588-019-0398-7.

[S7]. Hanna, C.W., Taudt, A., Huang, J., Gahurova, L., Kranz, A., Andrews, S., Dean, W., Stewart, A.F., Colomé-Tatché, M., and Kelsey, G. (2018). MLL2 conveys transcription-independent H3K4 trimethylation in oocytes. Nature Structural and Molecular Biology *25*. 10.1038/s41594-017-0013-5.

[S8]. Mei, H., Kozuka, C., Hayashi, R., Kumon, M., Koseki, H., and Inoue, A. (2021). H2AK119ub1 guides maternal inheritance and zygotic deposition of H3K27me3 in mouse embryos. Nature Genetics *53*. 10.1038/s41588-021-00820-3.

[S9]. Bourc'his, D., and Bestor, T.H. (2004). Meiotic catastrophe and retrotransposon reactivation in male germ cells lacking Dnmt3L. Nature. 10.1038/nature02886.

[S10]. Li, Y., Zhang, Z., Chen, J., Liu, W., Lai, W., Liu, B., Li, X., Liu, L., Xu, S., Dong, Q., et al. (2018). Stella safeguards the oocyte methylome by preventing de novo methylation mediated by DNMT1. Nature *564*. 10.1038/s41586-018-0751-5.
